# Comparative efficacy of probiotic, prebiotic, and synbiotic interventions in children with functional abdominal pain disorders: a systematic review and network meta-analysis

**DOI:** 10.3389/fnut.2026.1764837

**Published:** 2026-03-10

**Authors:** Yongrui Yang, Yunsong Li, Xiyu Yan, Wenrui Huang, Chunlan Cao, Haonan Yan, Li Leng

**Affiliations:** 1 The Second Affiliated Hospital of Guizhou University of Traditional Chinese Medicine, Guiyang, China; 2 Guizhou University of Traditional Chinese Medicine, Guiyang, China; 3 Guangzhou University of Traditional Chinese Medicine, Guangzhou, China

**Keywords:** children, functional abdominal pain disorders, network meta-analysis, prebiotics, probiotics

## Abstract

**Background:**

Functional abdominal pain disorders (FAPDs) are common pediatric gut–brain interaction disorders that substantially impair quality of life and lack effective pharmacologic or behavioral treatments. Given their biological plausibility, probiotics have emerged as a promising therapy, yet evidence remains inconsistent due to strain-specific effects and heterogeneous study designs.

**Methods:**

Eight databases (PubMed, Embase, Web of Science, CENTRAL, CNKI, VIP, Wanfang, and CBM) were searched up to October 2025 for randomized controlled trials (RCTs) evaluating probiotic interventions in children (4–18 years) with FAPDs. Eligible comparators included placebo, no treatment, or other probiotics. Primary outcomes were global improvement, complete pain resolution, pain severity, and pain frequency. Data were synthesized using a frequentist random-effects network meta-analysis (Stata 17.0, mvmeta command), and treatment efficacy was ranked by SUCRA. Risk of bias was assessed with the Cochrane RoB 2.0 tool, and evidence certainty with CINeMA. Sensitivity and meta-regression analyses evaluated the robustness of findings and the influence of dosage, duration, strain composition, age, and country.

**Results:**

21 RCTs (*n* = 1,807; published 2003–2023) were included, involving children aged 4–18 years with FAPDs diagnosed mainly by Rome III criteria. Interventions included various probiotic strains (e.g., *L. reuteri* DSM 17938, *L. rhamnosus* GG, *B. longum* DM8504, *Bacillus clausii*), synbiotics, and prebiotics. In the primary network meta-analysis, probiotics significantly improved all major outcomes versus placebo—global improvement (RR = 1.33, 95% CI 1.03–1.73), complete pain resolution (RR = 1.85, 1.07–3.21), pain severity (MD = −0.72, −1.16 to −0.28), and pain frequency (MD = −1.04, −1.98 to −0.11)—while prebiotics and synbiotics showed no significant benefit. Strain-level analyses identified *L. reuteri* DSM 17938, *L. rhamnosus* GG, Bifidobacterium mix, and *B. longum* (DM8504) as the most effective, with *B. clausii* consistently ineffective. Sensitivity and meta-regression analyses confirmed result robustness and found no effect modification by dose, duration, strain composition, or demographic factors.

**Conclusion:**

This network meta-analysis shows that probiotics significantly improve symptoms in children with functional abdominal pain disorders. Specific strains—*Lactobacillus reuteri* DSM 17938, *Lactobacillus rhamnosus* GG, Bifidobacterium mixtures, and *B. longum*—demonstrated the most consistent efficacy, supporting a shift toward strain-specific, mechanism-based probiotic therapy in pediatric FAPD.

**Systematic review registration:**

https://www.crd.york.ac.uk/PROSPERO/view/CRD420251218779, identifier CRD420251218779.

## Introduction

Functional abdominal pain disorders (FAPDs) are among the most common pediatric syndromes, characterized by chronic or recurrent abdominal pain without identifiable organic causes (1). According to the Rome IV criteria (2016), FAPDs have been redefined as “disorders of gut-brain interaction” (DGBIs), reflecting a deeper understanding of the bidirectional communication dysfunction between the central and enteric nervous systems (2). Globally, FAPDs affect approximately 13.5% of children (95% CI: 11.8%–15.3%), meaning more than one in nine children experience this condition (3). The chronic nature of DGBIs imposes a heavy burden, including school absenteeism, academic decline, and increased risk of anxiety and depression, all of which significantly impair quality of life (QoL) for both children and their families (1). Diagnosis often relies on exclusion, leading to extensive and costly investigations that strain healthcare resources (4, 5). Hence, there is an urgent need for safe, affordable, and effective therapeutic options.

Despite the significant disease burden, current standard treatments for pediatric FAPDs show a marked efficacy gap (6). The 2025 joint guidelines by the European and North American Societies for Pediatric Gastroenterology, Hepatology, and Nutrition (ESPGHAN/NASPGHAN) reviewed 86 randomized controlled trials (RCTs) and highlighted significant evidence gaps in this field (7). Pharmacologic therapies such as tricyclic antidepressants (TCAs) are used to modulate visceral hypersensitivity but show inconsistent efficacy in children and raise safety and off-label use concerns (8–10). Cognitive-behavioral therapy (CBT) is the most evidence-based nonpharmacologic option (11), yet its accessibility is limited by cost and the shortage of trained specialists (12). Dietary interventions, such as low-FODMAP diets, lack robust evidence in pediatrics and carry risks, including poor adherence, nutritional deficiencies, and disordered eating behaviors (13, 14). Consequently, many families turn to complementary and alternative medicine (CAM) in search of relief (6).

Given that FAPDs stem from gut-brain axis dysfunction, probiotics—targeting the gut microbiota—have emerged as a biologically plausible and promising therapeutic strategy (2, 15). Their potential mechanisms include modulation of the gut microbiome, anti-inflammatory effects, and reduction of visceral hypersensitivity (15). However, despite a sound biological rationale, clinical consensus remains elusive, and major guidelines offer conflicting recommendations (16, 17). The inconsistency arises from the high-strain and subtype-specific nature of probiotic effects (18). For instance, meta-analyses on *Limosilactobacillus reuteri* DSM 17938 have reached opposing conclusions, reporting both significant pain reduction (19) and lack of supportive evidence (20). Similarly, *Lactobacillus rhamnosus* GG (LGG) demonstrates benefit primarily in irritable bowel syndrome (IBS) subgroups but not in functional abdominal pain–not otherwise specified (FAP-NOS) or functional dyspepsia (FD) (18).

Traditional pairwise meta-analyses can only compare each probiotic strain with placebo, yet pediatric FAPD research lacks head-to-head RCTs comparing different active strains (21). As a result, clinicians and guideline developers remain unable to determine, for example, whether LGG or *L. reuteri* performs better in children with IBS (16). While some network meta-analyses (NMAs) exist for dietary interventions (22), they typically treat all probiotics as a single homogeneous category, overlooking strain-level distinctions and thereby obscuring the actual clinical effects (23). Therefore, there is a pressing need for the first strain-specific NMA comparing the relative efficacy and safety of various probiotic strains (e.g., *L. reuteri* DSM 17938, LGG) in children with FAPDs, ideally distinguishing between IBS and FAP-NOS subtypes. This study aims to integrate all available direct and indirect evidence to clarify current inconsistencies, rank probiotic interventions based on clinical outcomes such as pain relief and QoL improvement, and provide clear, actionable, evidence-based guidance for pediatric practice and future guideline development.

## Materials and methods

This systematic review and network meta-analysis was conducted in accordance with the PRISMA 2020 statement and the PRISMA extension for Network Meta-Analyses (PRISMA-NMA) guidelines. The study protocol was prospectively registered in the PROSPERO international database (registration number: CRD420251218779) to ensure methodological transparency and to minimize the risk of bias (24, 25).

### Search strategy

We systematically searched PubMed, Embase, Web of Science, CNKI, WanFang, CBM and the Cochrane Central Register of Controlled Trials (CENTRAL) for RCTs evaluating probiotic interventions in children with FAPDs, from database inception to October 30, 2025. Two reviewers independently conducted the literature search and screened studies according to predefined eligibility criteria. Any discrepancies were resolved through discussion or consultation with a third reviewer.

In addition, we manually searched the proceedings of major pediatric gastroenterology conferences, including those of the European Society for Paediatric Gastroenterology, Hepatology and Nutrition (ESPGHAN) and the North American Society for Pediatric Gastroenterology, Hepatology and Nutrition (NASPGHAN), as well as ClinicalTrials.gov and the World Health Organization International Clinical Trials Registry Platform (ICTRP) for ongoing or unpublished trials. References of included studies and relevant systematic reviews were also screened to identify additional eligible trials. The complete search strategies for each database are provided in Supplementary Appendix 1.

### Eligibility criteria

Eligible studies were RCTs that investigated the efficacy or safety of probiotic interventions in children with FAPDs. Participants were children aged 4 to 18 years, consistent with the Rome IV criteria, which define functional gastrointestinal disorders in this age group (26). Eligible diagnoses included irritable bowel syndrome (IBS), functional abdominal pain—not otherwise specified (FAP-NOS), and abdominal migraine, as defined by either Rome IV or earlier Rome criteria. Studies involving participants with organic gastrointestinal diseases (e.g., Hirschsprung’s disease), previous bowel surgery, or complex congenital disorders were excluded.

Interventions included probiotic preparations administered in any form (capsule, powder, liquid) and via any route (oral or rectal), whether as single strains or multi-strain combinations (including symbiotics). Trials in which probiotics were used as adjuncts to standard therapy were also eligible. The comparator could be placebo, no treatment, or another probiotic preparation. Studies evaluating prebiotics alone or other dietary interventions without a probiotic component were excluded.

The primary outcomes were four key clinical endpoints: (1) treatment success or global symptom improvement, (2) complete resolution of pain, (3) pain severity, and (4) pain frequency. Each outcome was defined according to the criteria used in individual trials. Studies that did not report at least one of these four outcomes were excluded from the analysis.

We included only peer-reviewed, full-text articles published in English. Conference abstracts, non-randomized studies, crossover trials, and trials with insufficient data were excluded.

### Screening process

Records retrieved from all databases were imported into EndNote 20.4.1 (Clarivate Analytics, Philadelphia, PA, USA), and duplicates were removed. The screening proceeded in three stages. First, two reviewers independently screened titles and abstracts, retaining uncertain records. Second, potentially eligible studies were evaluated against predefined criteria, with disagreements resolved by discussion and consultation with a third reviewer. Third, the full texts of shortlisted articles were reviewed to confirm eligibility.

### Data extraction

Predesigned extraction forms were developed *a priori* to record all relevant study features and results. Two reviewers independently extracted data, and discrepancies were resolved by discussion or consultation with a third reviewer. Extracted information included study characteristics (year, country, design, inclusion and exclusion criteria, and sample size), participant details (age, sex, disease subtype, and disease duration), and intervention features (type, strain, dosage, formulation, duration, and concurrent treatments). The comparators included placebo, no treatment, or other probiotic preparations. Extracted outcomes covered assessment timepoints, follow-up duration, and symptom scoring systems. The four primary outcomes were: (1) treatment success or global improvement, (2) complete resolution of pain, (3) pain severity, and (4) pain frequency. Trials that did not report at least one of these outcomes were excluded.

### Quality assessment of evidence

The methodological quality of all included trials was evaluated using the Cochrane Risk of Bias 2.0 tool, which examines random sequence generation, allocation concealment, blinding, completeness of outcome data, and selective reporting (27). Two reviewers independently performed the assessments, with discrepancies resolved by consensus. The CINeMA (Confidence in Network Meta-Analysis) framework was applied to grade the certainty of evidence across six domains: within-study bias, reporting bias, indirectness, imprecision, heterogeneity, and inconsistency (28, 29). Intransitivity was further assessed by comparing key effect modifiers—such as age, FAPD subtype, and baseline symptom severity—between studies contributing direct and indirect evidence.

### Statistical analysis

NMAs were conducted using Stata 17.0 with the mvmeta command, applying a multivariate random-effects model within a frequentist framework (30). Four primary outcomes were analyzed: global improvement or treatment success, complete resolution of pain, pain severity, and pain frequency. Relative risks (RRs) with corresponding 95% confidence intervals (CIs) were calculated for binary outcomes (global improvement and complete pain resolution), while standardized mean differences (SMDs) were used for continuous outcomes (pain severity and frequency). Random-effects models were estimated using the restricted maximum likelihood (REML) approach to account for between-study heterogeneity (31). The heterogeneity variance (τ^2^) was assumed to be common across treatment contrasts, with a between-study correlation coefficient fixed at 0.5 (11, 32, 33). Heterogeneity was categorized as low (< 0.04), low–moderate (0.04–0.16), moderate–high (0.16–0.36), or high (> 0.36).

The NMA was performed in two hierarchical stages. First, comparisons were made among placebo, prebiotic, probiotic, and synbiotic interventions. Second, a strain-level analysis was performed to evaluate individual probiotic agents. Consistency between direct and indirect evidence was assessed using the node-splitting method, while global inconsistency was examined via the design-by-treatment interaction model (34). Network geometry plots were constructed to visualize the evidence structure, and treatment ranking probabilities were estimated using the surface under the cumulative ranking curve (SUCRA), where higher values indicated greater efficacy (35).

To evaluate the robustness of the results, sensitivity analyses were conducted by excluding studies with high risk of bias or non–Rome diagnostic criteria. Meta-regression analyses were further performed to explore potential sources of heterogeneity, examining the influence of dosage, treatment duration, strain composition (single vs. multi-strain), country, and mean participant age. When standard deviations were missing, they were imputed from comparable studies reporting similar populations and outcomes.

## Results

### Literature selection

**FIGURE 1:**
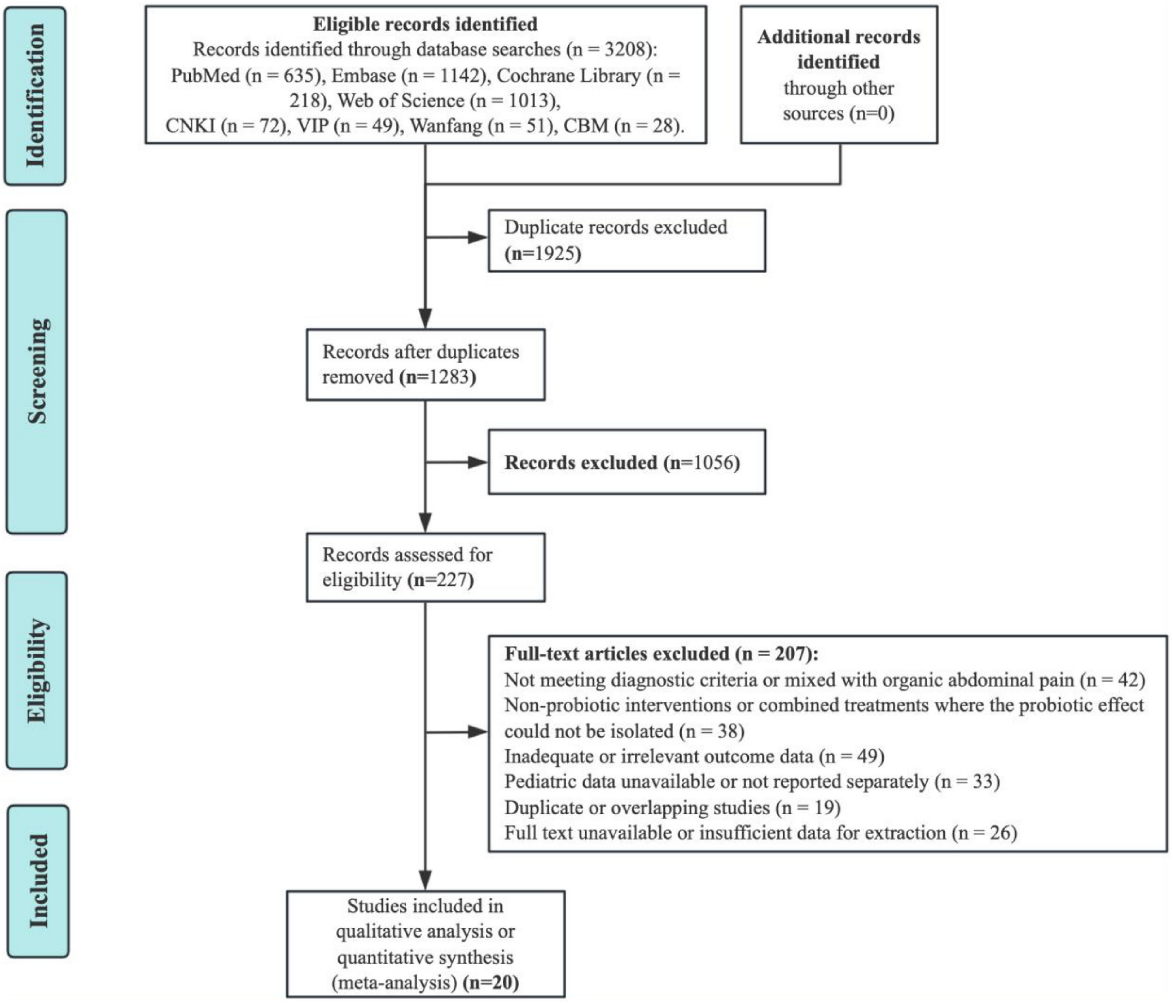
PRISMA flow diagram of study identification and selection process.

A total of 3,208 records were retrieved from eight databases, including PubMed (*n* = 635), Embase (*n* = 1,142), the Cochrane Library (*n* = 218), Web of Science (*n* = 1,013), CNKI (*n* = 72), VIP (*n* = 49), Wanfang (*n* = 51), and CBM (*n* = 28). After removing 1,925 duplicates, 1,283 records remained for title and abstract screening. Of these, 1,056 were excluded for being irrelevant to the study topic. Subsequently, 227 full-text articles were assessed for eligibility, and 207 were excluded due to: not meeting diagnostic criteria or inclusion of organic abdominal pain (*n* = 42); non-probiotic or combined interventions (*n* = 38); inadequate or irrelevant outcome data (*n* = 49); absence of pediatric-specific data (*n* = 33); duplicate or overlapping reports (*n* = 19); or unavailable/insufficient full texts (*n* = 26). Finally, 20 RCTs (36–55) met the inclusion criteria and were included in the qualitative and quantitative synthesis (Figure 1).

### Study characteristics

A total of 20 RCTs published between 2003 and 2023 were included, involving children with FAPDs diagnosed mainly according to Rome III (*n* = 15) and Rome IV (*n* = 1) criteria. The studies were conducted across 10 countries, predominantly Iran (5), Italy (4), and China (3), with sample sizes ranging from 40 to 259 participants. The mean participant age varied from 6 to 13 years. Interventions included synbiotics (*n* = 4), probiotics (*n* = 16), and various control conditions (placebo, prebiotic, or probiotic comparators). The most frequently studied strains were *Lactobacillus reuteri* DSM 17938 (*n* = 7), *Lactobacillus rhamnosus* GG (*n* = 3), Bacillus coagulans (*n* = 2), *B. subtilis* plus *E. faecium* (*n* = 2), and multi-strain combinations, such as the De Simone formulation (commercially marketed as VSL#3^®^ prior to 2016), which contains eight bacterial species (*Bifidobacterium breve*, *B. longum*, *B. infantis*, *Lactobacillus acidophilus*, *L. plantarum*, *L. casei*, *L. bulgaricus*, and *Streptococcus thermophilus*). It should be noted that post-2016 commercial VSL#3 formulations differ from the original De Simone formulation, which was available under the VSL#3^^®^^ trademark only until 2016 (56). Intervention durations ranged from 4 to 12 weeks, with most trials lasting 4 to 8 weeks. Overall, all trials compared probiotic or synbiotic preparations with placebo or inactive controls, and most reported outcomes related to pain improvement, frequency, severity, and treatment success (Table 1).

**TABLE 1:** Baseline characteristics of included studies.

Study ID	Country	Methods of diagnosis	FAPD diagnosis	Mean age	Intervention	Interventional agent	Control	Sample size	Duration
Asgarshirazi et al. (36)	Iran	Rome III	FAP/IBS/FD	7.44 ± 2.44/7.42 ± 2.49	Synbiotic	*B. coagulans* + FOS, 150 million spores + FOS BID	Placebo, 1 mg QD	29/25	4 wk
Baştürk et al. (37)	Turkey	Rome III	IBS	10.08 ± 4.49/10.20 ± 3.78/ 12.33 ± 4.65	Synbiotic	*B. lactis* B94 + inulin, 5 × 10^9^ CFU + 900 mg BID	Probiotic 5 × 10^9^ CFU BID/Prebiotic 900 mg BID	23/24/24	4 wk
Bauserman and Michail (38)	USA	Rome II	IBS	12.0 ± 3.1	Synbiotic	*L. rhamnosus* GG + inulin, 1 × 10^10^ CFU BID	Prebiotic, 1 capsule BID (dose unstated)	25/25	6 wk
Eftekhari et al. (39)	Iran	Rome III	FAP	6.26 ± 2.10/6.26 ± 2.61	Probiotic	*L. reuteri* (BioGaia^®^), 1 × 10^8^ CFU (5 drops/day)	Placebo, 5 drops/day	40/40	4 wk
Francavilla et al. (40)	Italy	Rome II	FAP/IBS	6.5 ± 2.1/6.3 ± 2.0	Probiotic	*L. rhamnosus* GG, 3 × 10^9^ CFU BID	Placebo, twice daily	67/69	8 wk
Gawrońska et al. (41)	Poland	Rome II	FAP/IBS/FD	11.9 ± 3/11.2 ± 2.7	Probiotic	*L. rhamnosus* GG, 3 × 10^9^ CFU BID	Placebo, twice daily	52/52	4 wk
Giannetti et al. (42)	Italy	Rome III	IBS/FD	IBS: 11.2 (range 8–17.9); FD: 11.6 (range 8–16.6)	Probiotic	*B. infantis* M-63 + *B. breve* M-16V + *B. longum* BB536, total 5 × 10^9^ CFU/day	Placebo, 1 sachet/day	24/24	6 wk
Guandalini et al. (43)	Italy/India	Rome II	IBS	12.5 (range 5–18)	Probiotic	Multi-strain (B. breve, B. longum, B. infantis, *L. acidophilus*, *L. plantarum*, *L. casei*, *L. bulgaricus*, *S. thermophilus*), 450B CFU/sachet, 1–2 sachets/day	Placebo, 1–2 sachets/day	59/59	6 wk
Jadrešin et al. (44)	Croatia	Rome III	FAP/IBS	Median 10.5 (range 5.4–17) vs. 9.5 (range 5.5–16.5)	Probiotic	*L. reuteri* DSM 17938 (BioGaia^®^), 1 × 10^8^ CFU/day (450 mg tablet)	Placebo, 1 tablet/day (450 mg)	26/29	12 wk
Jadrešin et al. (45)	Croatia	Rome III	FAP/IBS	Median 10.1/10.6	Probiotic	*L. reuteri* DSM 17938, 1 × 10^8^ CFU/day (450 mg tablet)	Placebo, 1 tablet/day (450 mg)	24/22	12 wk
Kianifar et al. (46)	Iran	Rome III	IBS	7.1 ± 0.3	Probiotic	*L. rhamnosus* GG, 1 × 10^10^ CFU BID	Prebiotic, 1 capsule BID	26/26	4 wk
Maragkoudaki et al. (47)	Athens/Ljubljana/ Poland	Rome III	FAP	9.2 ± 4.3/9.0 ± 3.2	Probiotic	*L. reuteri* DSM 17938, 2 × 10^8^ CFU/day (2 chewable tablets)	Placebo, 2 chewable tablets/day	27/27	4 wk
Rahmani et al. (48)	Iran	Rome III	FAP/IBS/ FD/AM	7.3 ± 1.7/7.7 ± 2.1	Probiotic	*L. reuteri*, 1 × 10^8^ CFU BID	Placebo, twice daily	65/60	4 wk
Romano et al. (49)	Italy	Rome III	FAP	10.2 ± 2.5/9.6 ± 0.4	Probiotic	*L. reuteri* DSM 17938, 1 × 10^8^ CFU BID (10 mL bottle)	Placebo, 10 mL twice daily	30/26	4 wk
Saneian et al. (50)	Iran	Rome III	FAP	9.0 ± 2.2/8.5 ± 2.2	Synbiotic	*B. coagulans* (Unique IS-2) + FOS, 150 million spores + 100 mg BID	Placebo, 1 tablet BID	59/58	4 wk
Weizman et al. (51)	Israel	Rome III	FAP	12.2 ± 2.8/11.7 ± 3.2	Probiotic	*L. reuteri* DSM 17938, 1 × 10^8^ CFU/day (chewable tablet)	Placebo, once daily	47/46	4 wk
Vázquez-Frias et al. (52)	Mexico	Rome IV	IBS	10.93 ± 3.30/11.19 ± 3.12	Probiotic	*B. clausii* (Enterogermina^®^, strains O/C, N/R, SIN, T), 4 × 10^9^ CFU/day (2 vials)	Placebo, 2 vials/day (5 mL each)	129/130	8 wk
Chen (53)	China	NA	FAP	6.92 ± 2.36/7.62 ± 2.57	Probiotic	*B. subtilis* + E. faecium (Peifeikang^®^), 3.3 × 10^8^–6.6 × 10^8^ CFU/day, 8 weeks	Placebo, not available	54/35	8 wk
Chen (54)	China	NA	FAP	7.6 ± 0.5	Probiotic	*B. longum* DM8504 (Lizhu Chang Le^®^), 2 × 10^9^ CFU/day (1 capsule BID)	Placebo, not available	40/40	6 wk
Wu (55)	China	NA	FAP	7.67 ± 1.65/7.60 ± 1.51	Probiotic	*B. subtilis* + E. faecium, 250 mg BID (≈5 × 10^8^ CFU/day, 8 weeks)	Placebo, not available	60/60	8 wk

FAPD, functional abdominal pain disorders; FAP, functional abdominal pain; IBS, irritable bowel syndrome; FD, functional dyspepsia; AM, abdominal migraine; CFU, colony-forming units; FOS, fructo-oligosaccharides; BID, twice daily; QD, once daily; wk, week; NA, not available; DSM, Deutsche Sammlung von Mikroorganismen (German Collection of Microorganisms); Rome II/III/IV, diagnostic criteria for functional gastrointestinal disorders. All probiotic dosages are expressed as daily intake unless otherwise stated.

### Risk of bias, certainty of evidence, and consistency

**FIGURE 2:**
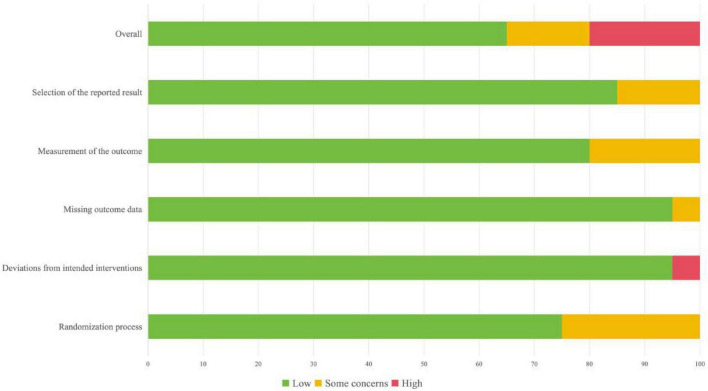
Risk of bias assessment of included studies. Risk of bias was evaluated using the Cochrane Risk of Bias 2 (RoB 2) tool. The figure presents the proportion of studies classified as low risk of bias, some concerns, or high risk of bias across each methodological domain. Green indicates low risk, yellow indicates some concerns, and red indicates high risk of bias.

The risk of bias was assessed using the Cochrane Risk of Bias 2.0 tool across five domains. Overall, the methodological quality of the included studies was moderate. Most trials (15/20) clearly stated randomization but lacked sufficient details on sequence generation and allocation concealment, raising some concerns in the randomization domain. Blinding of participants and outcome assessors was adequately described in approximately half of the studies. In contrast, several single-center or open-label trials provided insufficient information, raising concerns about deviations from intended interventions and outcome measurement. Missing outcome data were minimal across studies, with complete follow-up reported in the majority of cases. Selective reporting bias was suspected in a few studies that did not provide full statistical results or predefined outcomes. Overall, 13 trials were rated as having low risk of bias, 3 as having some concerns, and 4 as having high risk of bias (Figure 2 and Supplementary Appendix 2). For the consistency assessment, no significant inconsistency was detected between direct and indirect comparisons across the network. Node-splitting analysis showed no evidence of local inconsistency for any outcomes. The estimated τ^2^ values indicated low to moderate heterogeneity within the network, suggesting overall robust model consistency (Supplementary Appendix 3). After assessing the certainty of evidence using CINeMA, most pairwise and network comparisons demonstrated low to moderate confidence (Supplementary Appendix 5). All networks satisfied the assumption of transitivity, supporting the validity of indirect comparisons. Additionally, funnel plots showed no evident asymmetry, suggesting a low likelihood of small-study or publication bias (Supplementary Appendix 4).

### Primary network analysis

**FIGURE 3:**
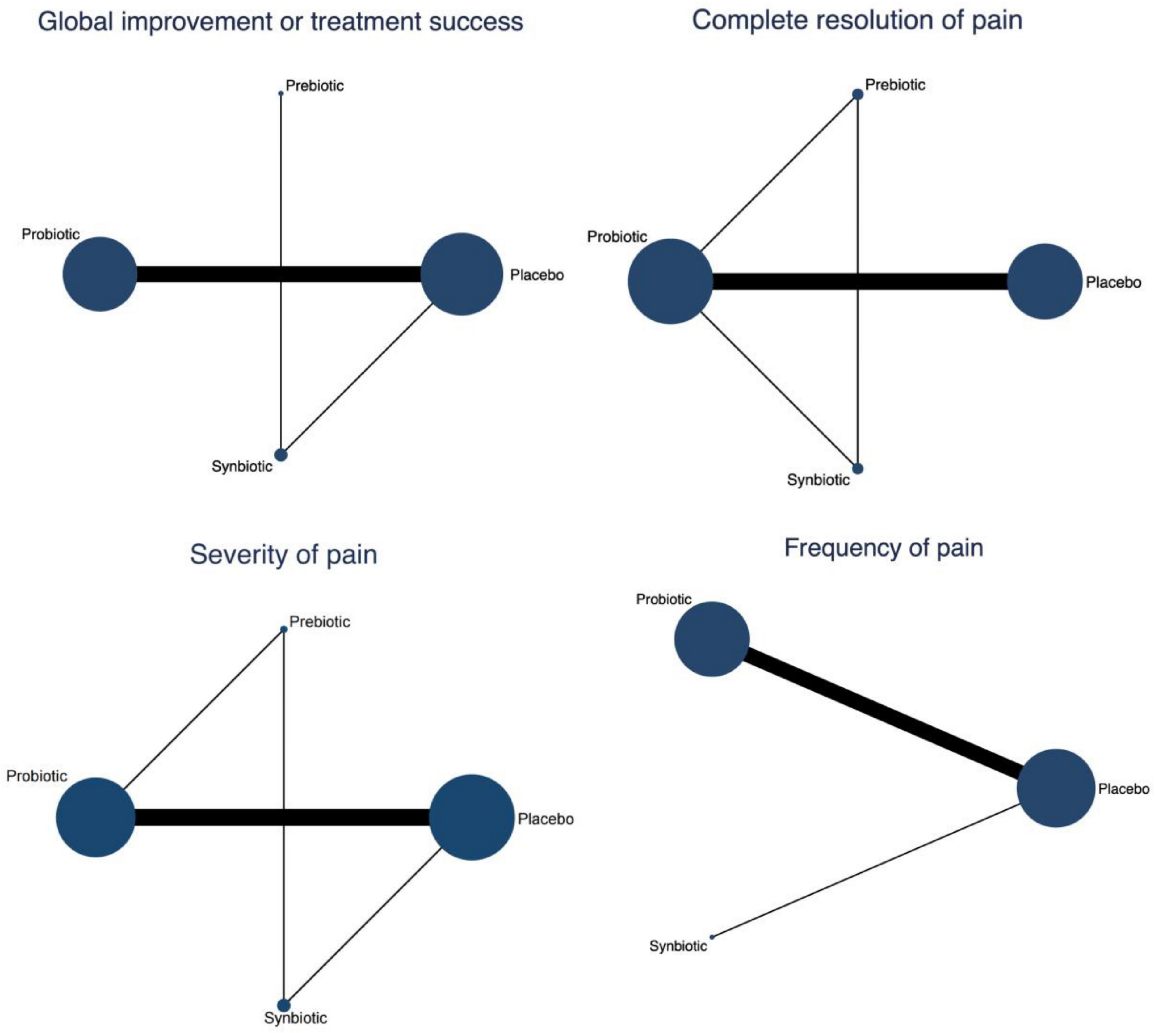
Network geometry of comparisons among placebo, prebiotic, probiotic, and synbiotic interventions across four outcomes.

**FIGURE 4:**
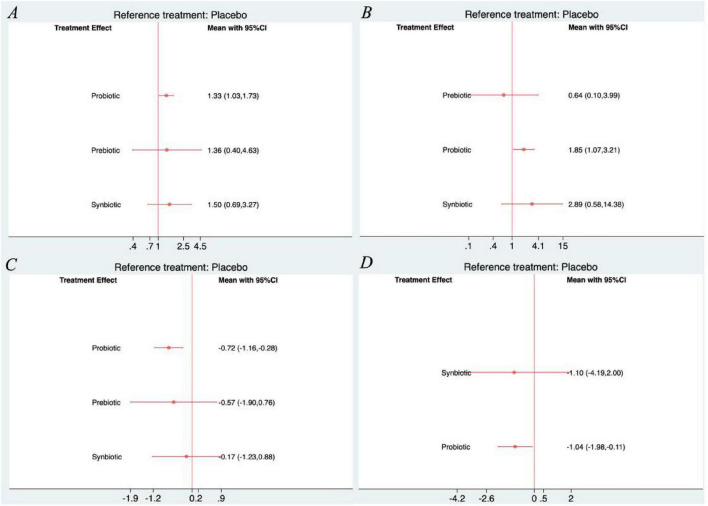
Forest plots of network meta-analysis for primary outcomes. **(A)** Global improvement or treatment success; **(B)** complete resolution of pain; **(C)** severity of pain; **(D)** frequency of pain.

All networks were well connected, with placebo serving as the most common comparator. The Probiotic–Placebo comparison was the most frequently studied across all outcomes, indicated by the thickest connecting lines. Both Synbiotic and Prebiotic interventions were involved in fewer trials but maintained indirect connections through placebo (Figure 3). As shown in Figure 4, the forest plots demonstrated that among all interventions, probiotics were the only treatment that significantly improved outcomes compared with placebo across all four primary endpoints. For global improvement or treatment success, probiotics showed a significant benefit over placebo (RR = 1.33, 95% CI: 1.03–1.73) with a SUCRA value of 62.4%. In terms of complete resolution of pain, probiotics again exhibited superiority to placebo (RR = 1.85, 95% CI: 1.07–3.21), achieving a SUCRA value of 71.5%. Regarding pain severity, probiotics were associated with a significant reduction compared with placebo (MD = −0.72, 95% CI: −1.16 to −0.28), with a SUCRA value of 80.6%. Similarly, for pain frequency, probiotics led to a significant decrease relative to placebo (MD = −1.04, 95% CI: −1.98 to −0.11), with a SUCRA of 73.5% (Supplementary Figures 6.1–6.4). No significant effects were observed for prebiotics or synbiotics across these outcomes. Furthermore, the league tables revealed no statistically significant differences among the active interventions (Supplementary Tables 7.1–7.4).

### Subgroup analysis

**FIGURE 5:**
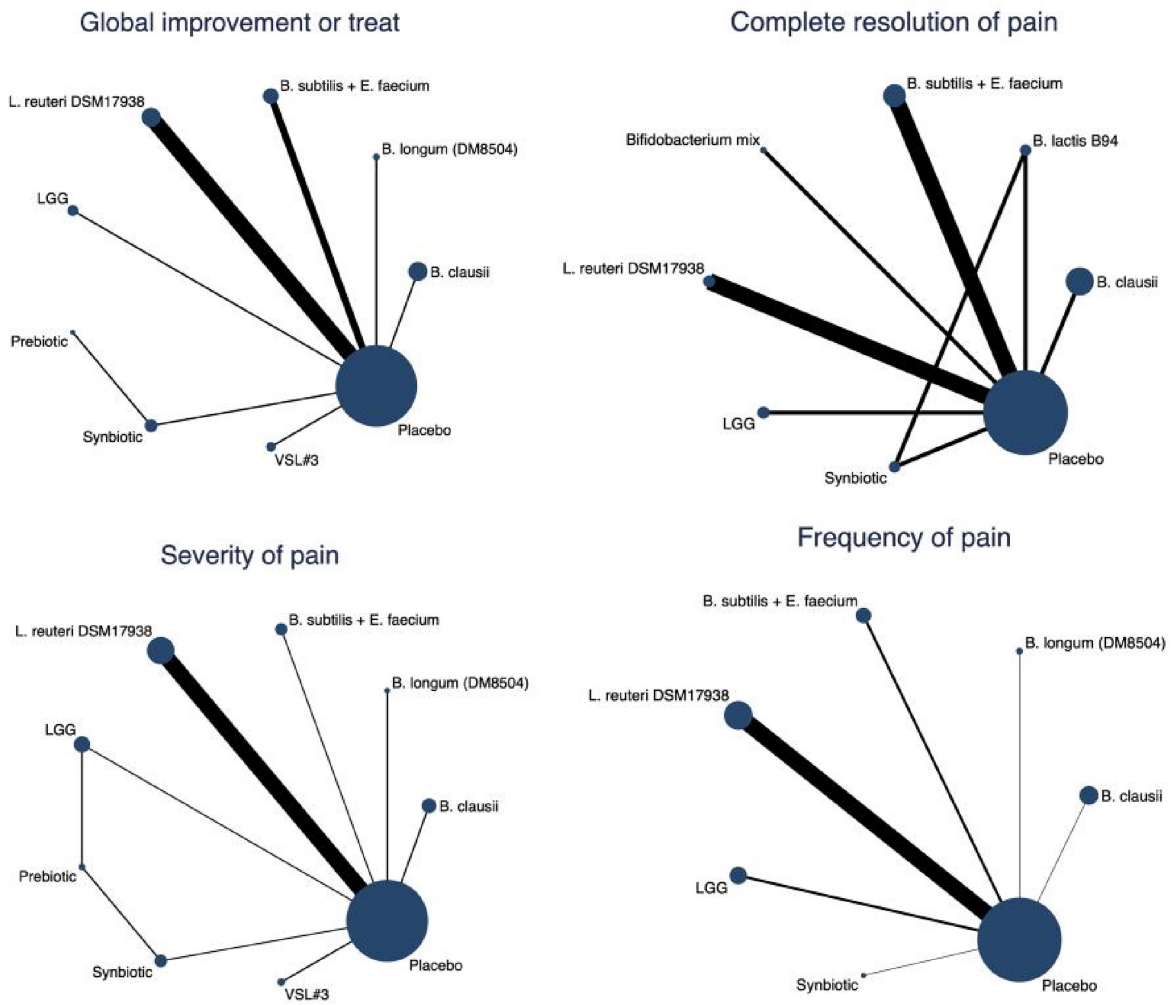
Network geometry of comparisons among individual probiotic and synbiotic strains across four outcomes.

The network plots in Figure 5 illustrate the comparative evidence across individual probiotic strains and synbiotic formulations for the four primary outcomes. Placebo served as the common comparator across all networks, with the largest number of studies evaluating *Lactobacillus reuteri* DSM 17938, LGG, and *Bacillus clausii*.

### Global improvement or treatment success

**FIGURE 6:**
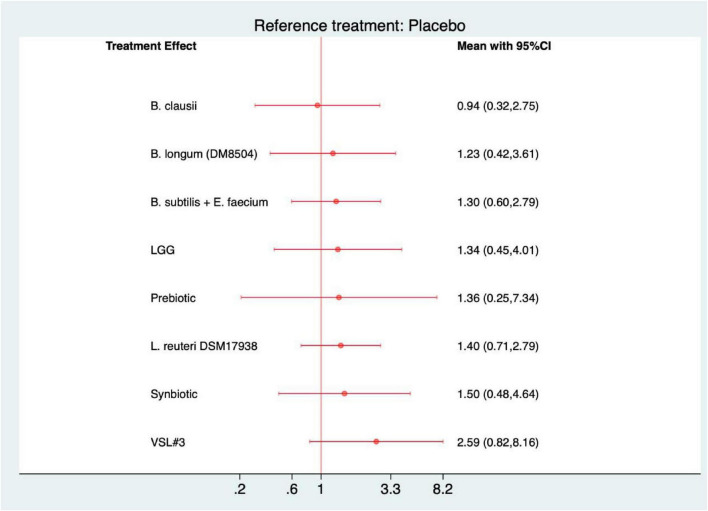
Forest plot of global improvement or treatment success (strain-level analysis).

In the network meta-analysis for global improvement or treatment success, none of the active interventions demonstrated a statistically significant advantage over placebo (Figure 6). Although most agents—including *L. reuteri* DSM 17938, VSL#3, and synbiotic formulations—showed a favorable trend toward improvement, the 95% credible intervals of all comparisons crossed the null line. Among tested interventions, VSL#3 ranked highest according to SUCRA values (81.3), followed by synbiotics (57.6) and *L. reuteri* DSM 17938 (55.4), suggesting potential clinical benefit that warrants confirmation in future trials (Supplementary Figure 6.5). The league table indicated no statistically significant differences between active interventions (Supplementary Table 7.5).

### Complete resolution of pain

**FIGURE 7:**
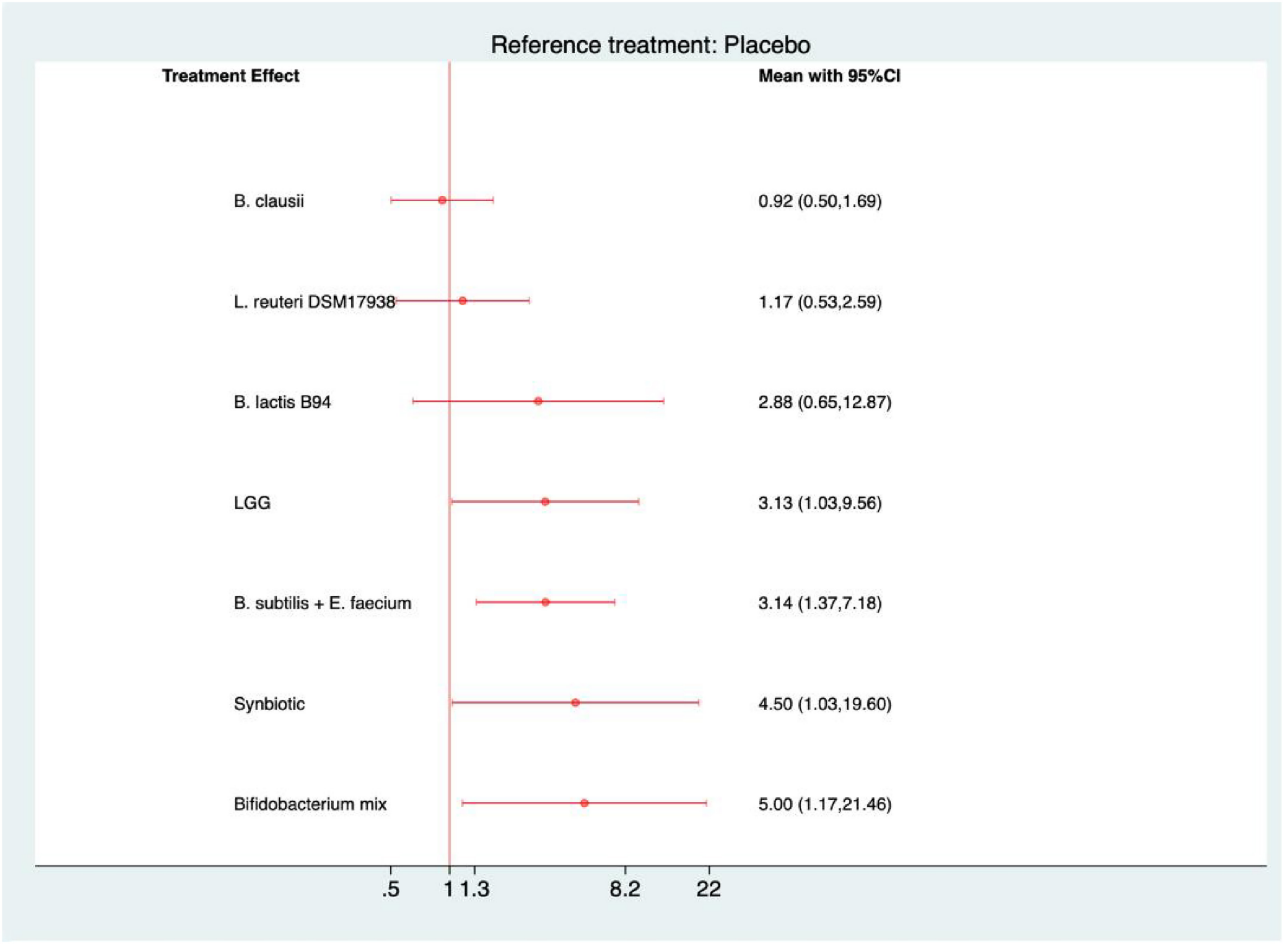
Forest plot of complete resolution of pain (strain-level analysis).

In the analysis of complete resolution of pain, all interventions except *B. clausii* showed greater efficacy compared with placebo (Figure 7). Four agents demonstrated statistically significant benefits: Bifidobacterium mix (RR = 5.00, 95% CI 1.17–21.46; SUCRA = 79.4), synbiotics (4.50, 1.03–19.60; 78.0), *B. subtilis* + *E. faecium* (3.14, 1.37–7.18; 65.9), and *L. rhamnosus* GG (3.13, 1.03–9.56; 65.1). According to the SUCRA ranking, Bifidobacterium mix and synbiotic formulations were the most promising interventions (Supplementary Figure 6.6). The league table further revealed that *B. clausii* was significantly less effective than *B. subtilis* + *E. faecium* (0.29, 0.10–0.81) and Bifidobacterium mix (0.18, 0.04–0.89). At the same time, no other pairwise comparisons showed significant differences (Supplementary Table 7.6).

### Severity of pain

**FIGURE 8:**
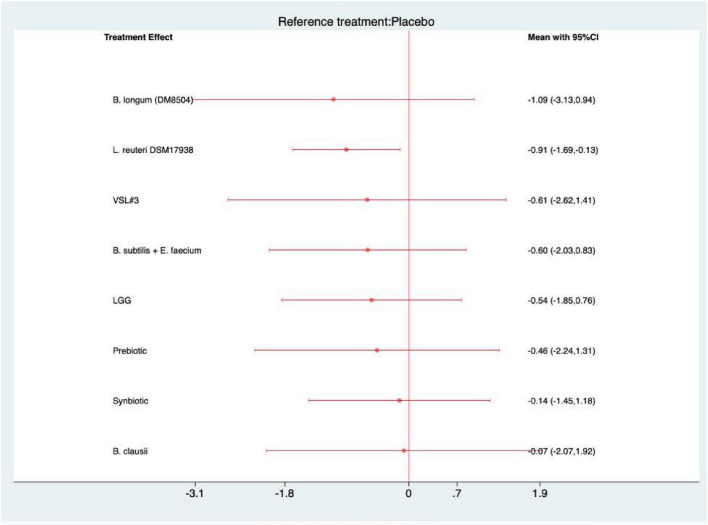
Forest plot of severity of pain (strain-level analysis).

For the outcome of severity of pain, all interventions demonstrated a trend toward symptom reduction compared with placebo (Figure 8). However, statistical significance was achieved only for *L. reuteri* DSM 17938 (MD = −0.91, 95% CI −1.69 to −0.13; SUCRA = 70.6), suggesting a modest yet reliable effect in alleviating pain intensity (Supplementary Figure 6.7). No statistically significant differences were detected among active interventions in the league table, indicating comparable efficacy across probiotic and synbiotic formulations for this endpoint (Supplementary Table 7.7).

### Frequency of pain

**FIGURE 9:**
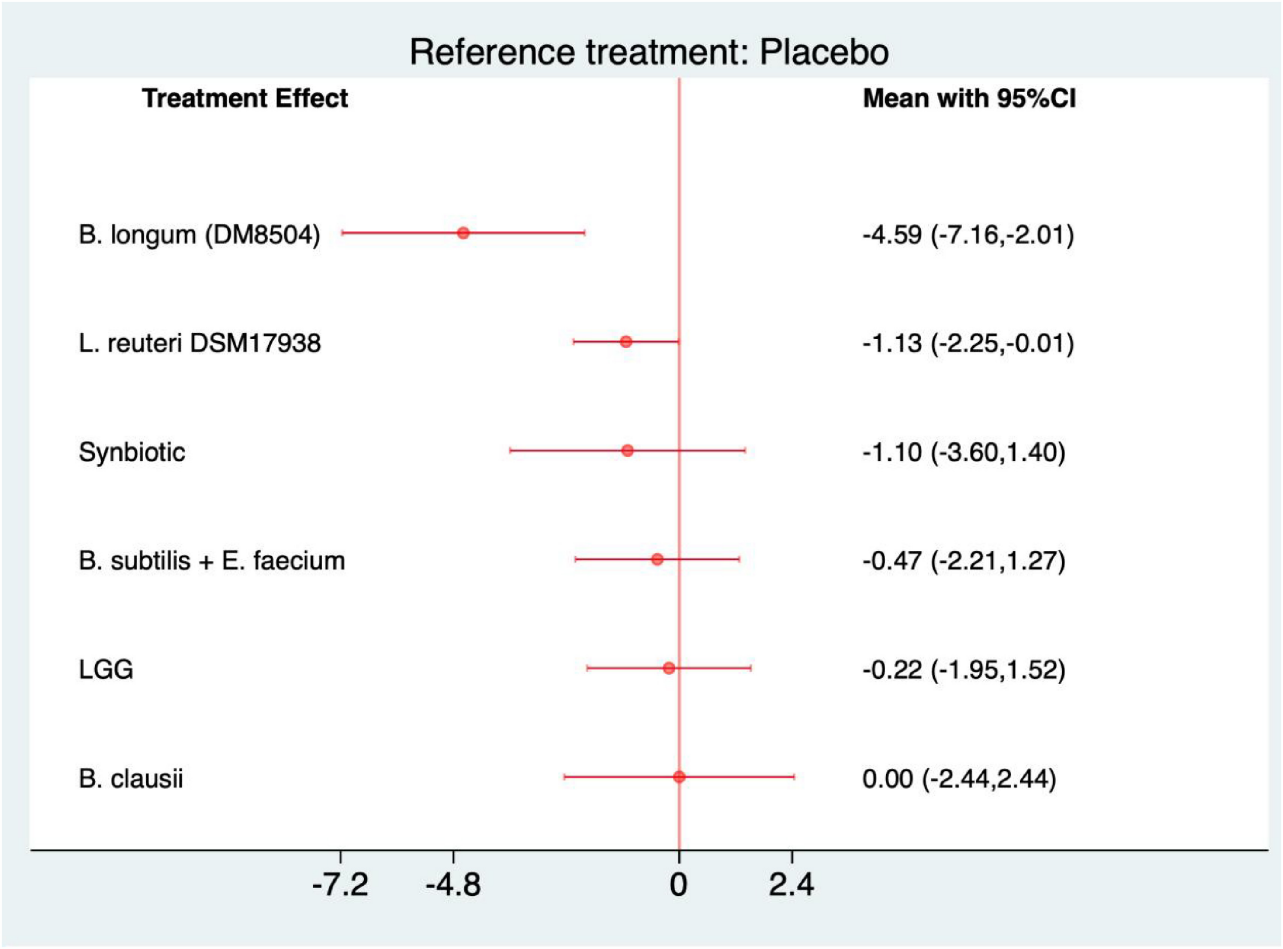
Forest plot of frequency of pain (strain-level analysis).

For frequency of pain, all interventions exhibited a decreasing trend compared with placebo (Figure 9). The most pronounced reduction was observed with Bifidobacterium longum (DM8504) (MD = −4.59, 95% CI −7.16 to −2.01; SUCRA = 99.3), indicating a robust and clinically meaningful improvement. *L. reuteri* DSM 17938 also achieved statistical significance (−1.13, −2.25 to −0.01; 63.7) (Supplementary Figure 6.8). Consistent with these findings, the league table revealed that *B. longum* (DM8504) outperformed *B. clausii*, *B. subtilis* + *E. faecium*, *L. reuteri* DSM 17938, and *L. rhamnosus* GG, underscoring its superior efficacy in reducing pain frequency among the tested interventions (Supplementary Table 7.8).

### Sensitivity analyses and meta-regressions

After excluding trials with high risk of bias or non–Rome diagnostic criteria, all network estimates remained directionally consistent with the primary analysis, confirming the robustness of the results. Probiotic interventions continued to show significant benefits. Overall, the pooled effects for probiotics, synbiotics, and other agents exhibited similar magnitudes to the full model, indicating stable and reliable findings across all outcomes (Supplementary Appendix 8).

No significant associations were observed between dosage, treatment duration, strain composition (single vs. multi-strain), country, or mean age and any of the four main outcomes. These findings suggest that the overall network estimates were robust and not substantially influenced by these potential covariates (Supplementary Appendix 9).

## Discussion

### Primary findings

The primary network meta-analysis revealed that probiotics consistently outperformed placebo across all major outcomes, demonstrating superior efficacy in improving global symptoms and reducing pain-related parameters. In contrast, prebiotics and synbiotics showed no statistically significant effects in any endpoint. When individual strains were examined, *L. reuteri* DSM 17938, *L. rhamnosus* GG, Bifidobacterium mixtures, and *B. longum* (DM8504) emerged as the most promising candidates for specific symptom domains, while *B. clausii* showed limited benefit. Sensitivity analyses excluding high-risk or non–Rome criteria trials confirmed the robustness of these findings, and meta-regression indicated that factors such as dose, treatment duration, strain composition, country, and age did not materially influence treatment effects. Overall, probiotics demonstrated a stable and clinically meaningful advantage for pediatric functional abdominal pain disorders.

Although the treatment effects were statistically reliable, their magnitudes were modest. In pediatric FAPDs, where symptoms often fluctuate, placebo responses are common, and treatment options remain limited, even small improvements may still lead to meaningful benefits in daily functioning and quality of life. However, these findings should not be interpreted as indicating large clinical effects. Treatment decisions should therefore consider efficacy alongside safety, tolerability, cost, and patient preference, with emphasis on strains supported by the most consistent evidence.

### Comparison with previous research

Our NMA demonstrated that probiotics, as a class effect, were superior to placebo in improving functional abdominal pain disorders in children. This aligns with earlier meta-analyses that observed similar beneficial trends for probiotics when data were pooled across trials. However, our findings contrast with several influential systematic reviews—such as Wegh et al. (20)—that concluded the current evidence was insufficient to recommend probiotics for clinical use. Reconciling this apparent discrepancy is essential to understanding the contribution of our study. The divergent conclusions likely stem from differences in statistical power and the handling of heterogeneity. Previous systematic reviews, including Wegh et al. (20), have performed strain-specific subgroup analyses (e.g., *L. reuteri*, LGG) that included only a few trials per strain (often fewer than five), resulting in low-quality and underpowered evidence (57). Consequently, these reviews could not detect significant strain-level effects and thus remained cautious about endorsing probiotics as a group. In contrast, our NMA aggregated all probiotic strains—such as *L. reuteri*, LGG, and Bifidobacterium—in comparison with placebo. This broader synthesis substantially increased sample size and statistical precision, allowing detection of a consistent and stable therapeutic signal at the class level. By integrating both direct and indirect evidence across multiple strains, the NMA design reduces the noise and heterogeneity inherent in small, isolated RCTs (17). Therefore, rather than contradicting prior reviews (5), our results extend their findings by confirming, through a more powerful and comprehensive analytical framework, the overall positive effect of probiotics that earlier analyses were likely unable to demonstrate due to limited statistical power.

### Strain-specific efficacy

This NMA advances beyond the broad question of whether probiotics work, addressing instead which specific strains provide real benefit (19). Our findings identified *Lactobacillus reuteri* DSM 17938, LGG, Bifidobacterium mixtures, and *B. longum* (DM8504) as effective, while *Bacillus clausii* showed no benefit—offering practical insight for clinical decision-making.

Both *L. reuteri* and LGG emerged as validated interventions for pediatric FAPDs, helping to reconcile long-standing inconsistencies in prior reviews. Earlier analyses diverged sharply: some [e.g., Trivić et al. (19)] supported *L. reuteri* for reducing pain intensity and increasing pain-free days, while others [e.g., Horvath et al. (18); Wegh et al. (20)] favored LGG, particularly in IBS subtypes. By integrating all available trial data, our NMA confirmed both strains as genuinely effective across the broader FAPD population, suggesting that prior discrepancies likely reflected small-sample limitations. Nonetheless, evidence indicates possible subtype specificity: LGG may perform better in IBS, whereas *L. reuteri* could be more beneficial for FAP-NOS (58). Although many original trials mixed FAPD subtypes or used outdated Rome criteria (59), our sensitivity analyses excluding non-Rome studies reinforced the robustness of these findings. Future head-to-head, subtype-stratified RCTs are warranted (23).

Importantly, our results highlight new therapeutic prospects. The efficacy of Bifidobacterium mixtures and *B. longum* (DM8504) extends current evidence beyond the traditional Lactobacillus-focused paradigm. Previous reports showed that Bifidobacterium combinations (*B. infantis*, *B. breve*, *B. longum*) may improve symptoms in pediatric IBS (57). The positive signal from *B. longum* (DM8504)—though based on limited pediatric data—is biologically plausible and supported by emerging findings in related strains. For instance, *B. longum* 35624 improved IBS symptoms in adolescents (60, 61), and *B. adolescentis* PRL2019 showed high response rates in a recent pediatric IBS trial (62). These results suggest that Bifidobacterium species may represent a new generation of microbiota-targeted interventions acting through neuromodulatory pathways rather than antimicrobial mechanisms.

Conversely, *B. clausii* showed no therapeutic benefit for FAPD—a clinically important negative finding. Prior studies similarly found *B. clausii* ineffective for functional constipation (63), IBS (52), and post-infectious abdominal pain (64), with its utility limited to acute infectious diarrhea (65, 66). This likely reflects a mechanistic mismatch: FAPD is not an infectious disorder but one driven by gut–brain axis dysregulation and visceral hypersensitivity (15, 67). In contrast, effective strains such as *L. reuteri* and Bifidobacterium species exhibit neuromodulatory properties that directly address FAPD pathophysiology (62, 67). Collectively, these findings emphasize that clinicians should move away from empirically using anti-diarrheal probiotics like *B. clausii* for abdominal pain management.

### Reassessing prebiotics and synbiotics

This NMA provided clear evidence of no significant benefit from prebiotics or synbiotics in managing pediatric FAPDs. The lack of efficacy for prebiotics aligns with existing literature. Evidence supporting their use—typically involving fermentable fibers such as inulin or fructooligosaccharides (FOS)—is sparse and of very low quality (68). Moreover, high doses of prebiotics may actually exacerbate symptoms in patients with FAPD or IBS, as these substrates are highly fermentable FODMAPs that increase intestinal gas and distension, thereby triggering discomfort and pain (68).

For synbiotics, our findings challenge earlier meta-analyses such as the Cochrane review (69), which suggested a possible benefit (RR 1.34, 95% CI 1.03–1.74). However, that review graded the certainty of evidence as “low” or “very low,” citing high heterogeneity across just four trials—each testing a distinct synbiotic formulation (69). By pooling a broader set of studies within a network meta-analysis framework, our results likely offer a more stable and reliable estimate, indicating no consistent clinical effect.

Mechanistically, the concept of synbiotics may be inherently flawed in FAPD. The disorder’s hallmark is visceral hypersensitivity—an exaggerated response to gut distension and fermentation. Prebiotics, by definition, are substrates selectively fermented by gut microbiota to produce gas and short-chain fatty acids (70); in sensitive patients, this process may worsen symptoms rather than relieve them (68). Thus, combining probiotics (which aim to modulate neural and immune signaling) with prebiotics (which enhance fermentation) could represent a mechanistic contradiction. The present findings—showing probiotics effective but synbiotics ineffective—support this “mechanism antagonism” hypothesis, underscoring the need for a more nuanced approach to microbiota-targeted therapies in FAPD.

### Biological basis of probiotic analgesia

This NMA confirmed a strain-specific therapeutic effect, implying that efficacy stems from mechanism-specific actions. FAPDs are characterized by gut–brain axis dysregulation and visceral hypersensitivity (VHS) (15, 67). The probiotic strains showing benefit in our analysis—*Lactobacillus reuteri*, LGG, and Bifidobacterium species—share a distinct ability to influence these neural and immune pathways.

*L. reuteri* DSM 17938 has been shown to modulate pain perception and gut motility (48, 71). Mechanistically, it enhances the expression of peripheral opioid receptors in the colon, thereby dampening visceral hypersensitivity to distension (67). Animal models further demonstrate that *L. reuteri* reduces dorsal root ganglion excitability, acting as both a neural modulator and a direct analgesic (72). LGG appears to operate through barrier protection and anti-inflammatory pathways. It secretes bioactive proteins (p40, p75) that prevent epithelial apoptosis and strengthen mucosal integrity (73). Recent work has shown that LGG can metabolize dietary tryptophan into methylnicotinamide (MNA), which stabilizes the epithelial and blood–brain barriers and limits inflammation (74, 75). Thus, LGG functions primarily as a barrier-restoring and immunoregulatory agent. Bifidobacterium species—particularly *B. longum* (DM8504) and *B. adolescentis*—may act through neurotransmitter-mediated analgesia. Emerging evidence indicates that certain strains (e.g., *B. adolescentis* PRL2019) produce γ-aminobutyric acid (GABA), the brain’s primary inhibitory neurotransmitter (62). By elevating luminal GABA levels, Bifidobacterium may increase the visceral pain threshold, functioning as a microbial GABA factory.

Collectively, these mechanisms converge on a unified concept: effective probiotic strains alleviate pain not by altering microbiota composition alone, but by modulating neuroimmune and neurochemical signaling along the gut–brain axis—a pathway central to FAPD pathophysiology.

### Strengths, robustness, and methodological limitations

#### Strengths and robustness

This NMA offers a major methodological advantage by allowing simultaneous comparison of multiple interventions—including probiotics, prebiotics, and synbiotics—as well as individual probiotic strains, even in the absence of direct head-to-head trials (59, 76). Sensitivity analyses, which excluded studies at high risk of bias and those using non-Rome diagnostic criteria, confirmed the stability of the primary findings. This is particularly important because earlier definitions of “recurrent abdominal pain” were broad, whereas the Rome III and especially Rome IV criteria (77) provide a more rigorous diagnostic framework. Our results remained consistent even under these stricter definitions, reinforcing both the clinical validity and internal reliability of the findings.

Meta-regression further supported the robustness of our conclusions by showing that dose, treatment duration, strain composition (single vs. multi-strain), country, and age did not significantly modify treatment effects. This contrasts with prior evidence in other conditions such as antibiotic-associated diarrhea (AAD), where higher doses (> 5 × 10^9^ CFU/day) were considered essential (78), or irritable bowel syndrome (IBS), where longer treatment duration appeared beneficial (79). Our findings suggest that, for FAPDs, probiotic efficacy may follow a threshold rather than dose-dependent model—once a minimal biological activation threshold [e.g., ≥ 10^8^ CFU/day sustained for several weeks (1)] is reached, further increases may not enhance benefit. Similarly, the lack of difference between single- and multi-strain formulations underscores that mechanism matters more than quantity: a single strain with the right neuroimmune mechanism (e.g., *L. reuteri*) may outperform a non-synergistic combination.

### Limitations

Despite its strengths, this NMA has inherent limitations. The evidence network was sparse (80), as most interventions were compared only with placebo, with few direct head-to-head studies (59). In addition, several strain-specific estimates were informed by single or few trials, resulting in wide confidence intervals and limited precision of effect estimates. This imprecision reflects the inherent evidence fragmentation characteristic of strain-level analyses rather than inconsistency of treatment effects. Consequently, findings related to sparsely studied strains should be interpreted cautiously and considered hypothesis-generating. Substantial clinical heterogeneity remained across trials in strain selection, dose, duration, and outcome definitions (57). Although meta-regression analyses did not detect significant effect modification by dose or treatment duration, these findings should be interpreted cautiously given the limited number of studies available for each covariate. The absence of statistically detectable interaction effects may therefore reflect limited analytical power rather than true clinical equivalence. The inclusion of studies using both Rome III and Rome IV diagnostic criteria (77) may have introduced variability in patient populations (81). Importantly, the network incorporated trials enrolling different FAPD subtypes (IBS, FAP-NOS, FD, and AM), which may represent distinct underlying pathophysiological mechanisms and treatment response profiles. Such population mixing may challenge the transitivity assumption underlying network meta-analysis, as differential treatment effects across subtypes cannot be fully excluded. Although sensitivity analyses were conducted, the limited number of studies within individual subtypes precluded robust subtype-restricted network evaluations. Consequently, strain-specific effects may have been attenuated or obscured. Finally, publication bias remains a recognized issue in pediatric probiotic research (82), as studies with positive results are more likely to be published, potentially inflating pooled estimates. In sum, while the findings are methodologically sound and clinically meaningful, they should be interpreted in light of these limitations and further validated through well-designed, subtype-specific head-to-head RCTs.

### Clinical implications and future research directions

This NMA provides the strongest evidence to date supporting specific probiotics as effective treatments for pediatric FAPDs. It shifts the focus from whether probiotics should be used to which strains to select. Among all options, *Lactobacillus reuteri* DSM 17938 and *L. rhamnosus* GG show the most consistent efficacy and should be prioritized. Bifidobacterium mixtures and *B. longum* strains (e.g., DM8504 or 35624) appear promising for children with IBS-like symptoms, while *Bacillus clausii* should be avoided Prebiotics and synbiotics remain unsupported and may even worsen symptoms through gas production. Meta-regression further indicates that efficacy depends more on selecting the right strain than on dose or formulation.

Future work should refine these insights through targeted, high-quality RCTs. Large, double-blind head-to-head trials are needed to compare *L. reuteri* DSM 17938, *L. rhamnosus* GG, and *B. longum*. Studies must also apply strict Rome IV definitions and investigate subtype-specific effects, distinguishing FAP-NOS from IBS populations. Validation of emerging strains such as *B. longum* DM8504 is essential. Finally, research should integrate mechanistic endpoints, linking clinical outcomes to gut–brain axis modulation through biomarkers like fecal GABA, MNA, and barrier integrity measures. Such designs will bridge mechanism and efficacy, advancing toward truly personalized probiotic therapy for children with FAPDs.

## Conclusion

This network meta-analysis provides the most comprehensive synthesis of current evidence suggesting that probiotics, as a class, may offer clinical benefits for children with functional abdominal pain disorders. Among individual strains, *Lactobacillus reuteri* DSM 17938, *Lactobacillus rhamnosus* GG, Bifidobacterium mixtures, and *B. longum* demonstrated favorable efficacy profiles, while prebiotics and synbiotics showed no consistent therapeutic advantage. However, the certainty of evidence for most comparisons ranged from low to moderate, warranting cautious interpretation of strain-specific effects and treatment rankings. These findings support the targeted evaluation of specific probiotic strains rather than generic supplementation, marking a shift toward mechanism-driven, evidence-based microbiota therapy in pediatric gastroenterology. Future adequately powered, subtype-specific head-to-head trials are required to confirm these findings and strengthen the evidence base for clinical decision-making.

## Data Availability

The original contributions presented in this study are included in this article/Supplementary material, further inquiries can be directed to the corresponding author.
