# Supplementary material for: Comparative efficacy of probiotic, prebiotic, and synbiotic interventions in children with functional abdominal pain disorders: a systematic review and network meta-analysis
Source: Front Nutr. 2026 Mar 10;13:1764837. doi: 10.3389/fnut.2026.1764837 (PMC13008698; doi:10.3389/fnut.2026.1764837)
Supplement: Supplementary file 1 [file Supplementary_file_1.docx]

**Appendix 1: Search strategy**

**Table S1. Search strategy of PubMed**

| **#** | **Searches** |
| --- | --- |
| 1 | ("Probiotics"[Mesh] OR probiotic*[tiab] OR "Saccharomyces"[Mesh] OR (Saccaromyce*[tiab] OR boulardii[tiab]) OR "Lactobacillus"[Mesh] OR (lactobacil*[tiab] OR Betabacterium[tiab] OR Lactobacteria[tiab] OR "lactic acid bacteria"[tiab] OR casei[tiab] OR paracasei[tiab] OR rhamnosus[tiab] OR helveticus[tiab] OR acidophilus[tiab]) OR "Bifidobacterium"[Mesh] OR Bifidobacter*[tiab] OR "Escherichia coli"[Mesh] OR ("Escherichia coli"[tiab] OR E.Coli[tiab] OR "E. Coli"[tiab] OR Mutaflor[tiab] OR Colinfant[tiab]) OR "Streptococcus"[Mesh] OR (Streptococcus[tiab] OR Streptococceae[tiab] OR "VSL#3"[tiab] OR "VSL #3"[tiab]) OR "Bacillus"[Mesh] OR Bacillus[tiab] OR "Clostridium butyricum"[Mesh] OR "clostridium butyricum"[tiab] OR "Enterococcus"[Mesh] OR (enterococcus[tiab] OR faecalis[tiab]) OR ("Biok+"[tiab] OR Lacidofil[tiab] OR Lactogermine[tiab] OR "Pb Probinul"[tiab] OR "BIfido Triple"[tiab]) OR (Commensal*[tiab] OR yeast[tiab] OR Fung*[tiab])) |
| 2 | ("Functional Gastrointestinal Disorders"[Mesh] OR "Irritable Bowel Syndrome"[Mesh] OR "Dyspepsia"[Mesh] OR "Abdominal Pain"[Mesh] OR (functional gastrointestinal disorder*[tiab] OR FGIDs[tiab] OR (irritable bowel[tiab] OR irritable colon*[tiab] OR IBS[tiab]) OR (dyspepsia[tiab] OR dyspeptic[tiab] OR indigestion[tiab] OR NUD[tiab] OR FD[tiab]) OR ((abdominal[tiab] OR abdomen[tiab] OR bowel[tiab] OR stomach[tiab] OR epigastric[tiab]) AND (pain*[tiab] OR migraine*[tiab] OR colic*[tiab] OR discomfort*[tiab] OR ache*[tiab] OR aching[tiab] OR soreness[tiab])) OR ((abdominal[tiab] OR abdomen[tiab]) AND migraine*[tiab]) OR (functional abdominal[tiab] OR FAP[tiab] OR CFAP[tiab]))) |
| 3 | ("Child"[Mesh] OR "Adolescent"[Mesh] OR "Infant"[Mesh] OR "Minors"[Mesh] OR "Pediatrics"[Mesh] OR "Puberty"[Mesh] OR "Schools"[Mesh] OR (baby[tiab] OR babies[tiab] OR child*[tiab] OR pediatric*[tiab] OR paediatric*[tiab] OR peadiatric*[tiab] OR infan*[tiab] OR neonat*[tiab] OR newborn*[tiab] OR kid*[tiab] OR adolescen*[tiab] OR preschool[tiab] OR pre-school[tiab] OR toddler*[tiab] OR boy*[tiab] OR girl*[tiab] OR teen*[tiab] OR minors[tiab] OR prepubescen*[tiab] OR postpubescen*[tiab] OR pubescen*[tiab] OR youth*[tiab] OR young[tiab] OR student*[tiab] OR schoolchild*[tiab] OR "school age"[tiab] OR underage*[tiab] OR "under 18"[tiab] OR "under 16"[tiab])) |
| 4 | ("Randomized Controlled Trial"[Publication Type] OR "Controlled Clinical Trial"[Publication Type] OR (randomized[tiab] OR randomly[tiab] OR placebo[tiab] OR trial[tiab] OR groups[tiab] OR "drug therapy"[sh])) |
| 5 | #1 AND #2 AND #3 AND #4 |

**Appendix 2: Risk of bias of randomized clinical trials**

**Table S2: Study level risk of bias assessment using Cochrane risk of bias tool 2.0 for assessing risk of bias of randomized clinical trials**

| **Study ID** | **Randomization process** | **Deviations from intended interventions** | **Missing outcome data** | **Measurement of the outcome** | **Selection of the reported result** | **Overall** |
| --- | --- | --- | --- | --- | --- | --- |
| Asgarshirazi 2015 | Some concerns | High | Low | Some concerns | Low | High |
| Baştürk 2016 | Low | Low | Some concerns | Low | Low | Some concerns |
| Bauserman 2005 | Low | Low | Low | Low | Low | Low |
| Eftekhari 2015 | Low | Low | Low | Low | Low | Low |
| Francavilla 2010 | Low | Low | Low | Low | Low | Low |
| Gawrońska 2007 | Low | Low | Low | Low | Low | Low |
| Giannetti 2017 | Low | Low | Low | Low | Low | Low |
| Guandalini 2010 | Low | Low | Low | Low | Low | Low |
| Jadrešin 2017 | Low | Low | Low | Low | Low | Low |
| Jadrešin 2020 | Low | Low | Low | Low | Low | Low |
| Kianifar 2015 | Some concerns | Low | Low | Low | Low | Some concerns |
| Maragkoudaki 2017 | Low | Low | Low | Low | Low | Low |
| Rahmani 2020 | Some concerns | Low | Low | Low | Low | Some concerns |
| Romano 2014 | Low | Low | Low | Low | Low | Low |
| Saneian 2015 | Low | Low | Low | Low | Low | Low |
| Weizman 2016 | Low | Low | Low | Low | Low | Low |
| Vázquez 2023 | Low | Low | Low | Low | Low | Low |
| Chen 2008 | Some concerns | Low | Low | Some concerns | Some concerns | High |
| Chen 2017 | Low | Low | Low | Some concerns | Some concerns | High |
| Wu 2013 | Some concerns | Low | Low | Some concerns | Some concerns | High |

**Appendix 3: Evaluation of inconsistency and heterogeneity**

**Table S3.1: Global consistency**

| **NMA** | **Clinical outcome** | **Chi square** | **P value** | **Tau²** |
| --- | --- | --- | --- | --- |
| **Primary** | Global improvement or treatment success | 0.04 | 0.843 | 0.120788 |
|  | Complete resolution of pain | 0.29 | 0.5906 | 0.129662 |
|  | Severity of pain | 0.62 | 0.4326 | 0.658787 |
|  | Frequency of pain | 0.48 | 0.4871 | 0.409678 |
| **Subgroup** | Global improvement or treatment success | 2.63 | 0.1046 | 0.295766 |
|  | Complete resolution of pain | 4.03 | 0.0448 | ＜0.000001 |
|  | Severity of pain | 0.65 | 0.4213 | 0.422085 |
|  | Frequency of pain | 0.74 | 0.3892 | 0.537644 |

**Table S3.2: Node-splitting results for the primary analysis of complete resolution of pain**

| Side | | Direct | | Indirect | | Difference | | P>\|z\| | tau |
| --- | --- | --- | --- | --- | --- | --- | --- | --- | --- |
|  |  | Coef. | Std.Err. | Coef. | Std.Err. | Coef. | Std.Err. |  |  |
| Placebo | Probiotic | 0.4105049 | 0.2245084 | 0.0754729 | 412.1173 | 0.335032 | 412.1174 | 0.999 | 0.360085 |
| Prebiotic | Probiotic | 0.8472979 | 0.7228552 | 1.48795 | 1045.47 | -0.6406518 | 1045.47 | 1 | 0.3600867 |
| Probiotic | Synbiotic | 0.293874 | 0.5463373 | -0.3536773 | 1028.233 | 0.6475514 | 1028.233 | 0.999 | 0.3600849 |

**Table S3.3: Node-splitting results for the subgroup analysis of complete resolution of pain**

| Side | | Direct | | Indirect | | Difference | | P>\|z\| | tau |
| --- | --- | --- | --- | --- | --- | --- | --- | --- | --- |
|  |  | Coef. | Std.Err. | Coef. | Std.Err. | Coef. | Std.Err. |  |  |
| B. clausii | Placebo | 0.086623 | 0.3104209 | -0.9161588 | 119.1794 | 1.002782 | 119.1798 | 0.993 | 1.11E-08 |
| B. lactis B94 | Placebo | -1.058607 | 0.763304 | 1.231789 | 631.0266 | -2.290396 | 631.028 | 0.997 | 4.03E-08 |
| B. subtilis + E. faecium | Placebo | -1.144806 | 0.421643 | 1.318049 | 444.284 | -2.462855 | 444.2846 | 0.996 | 9.24E-11 |
| Bifidobacterium mix | Placebo | -1.609438 | 0.7432234 | 1.782674 | 635.4116 | -3.392112 | 635.4129 | 0.996 | 1.86E-08 |
| L. reuteri DSM17938 | Placebo | -0.1568616 | 0.4050864 | 0.3300914 | 450.0321 | -0.486953 | 450.0326 | 0.999 | 3.07E-08 |
| LGG | Placebo | -1.142097 | 0.56907 | 1.315327 | 631.7545 | -2.457425 | 631.7553 | 0.997 | 5.61E-08 |
| Synbiotic | Placebo | -1.504077 | 0.7506611 | 0.7863719 | 630.9274 | -2.290449 | 630.9281 | 0.997 | 2.59E-08 |

**Table S3.4: Node-splitting results for the subgroup analysis of severity of pain**

| Side | | Direct | | Indirect | | Difference | | P>\|z\| | tau |
| --- | --- | --- | --- | --- | --- | --- | --- | --- | --- |
|  |  | Coef. | Std.Err. | Coef. | Std.Err. | Coef. | Std.Err. |  |  |
| B. clausii | Placebo | 0.0718987 | 1.018606 | 0.7089375 | 76.65512 | -0.6370389 | 76.66189 | 0.993 | 1.010991 |
| B. longum (DM8504) | Placebo | 1.092987 | 1.039239 | -0.9489699 | 632.2986 | 2.041957 | 632.2996 | 0.997 | 1.01098 |
| B. subtilis + E. faecium | Placebo | 0.5970127 | 0.729459 | -0.4530038 | 447.1807 | 1.050017 | 447.1813 | 0.998 | 1.010981 |
| L. reuteri DSM17938 | Placebo | 0.9050747 | 0.3979374 | -0.7611663 | 239.1559 | 1.666241 | 239.1563 | 0.994 | 1.010984 |
| LGG | Prebiotic | 0.5781576 | 1.072346 | -0.9094454 | 1.507311 | 1.487603 | 1.849841 | 0.421 | 1.03415 |
| LGG | Placebo | 0.3013923 | 0.7429488 | 1.788932 | 1.694056 | -1.48754 | 1.849811 | 0.421 | 1.034147 |
| Prebiotic | Synbiotic | 0.828852 | 1.075664 | -0.6587751 | 1.504942 | 1.487627 | 1.849839 | 0.421 | 1.034148 |
| Synbiotic | Placebo | 0.3820055 | 0.7503117 | -1.105601 | 1.69081 | 1.487607 | 1.849813 | 0.421 | 1.034147 |
| VSL#3 | Placebo | 0.6057693 | 1.028396 | -0.4617459 | 631.9622 | 1.067515 | 631.9631 | 0.999 | 1.01098 |

**Appendix 4: Funnel plots**

**Figure S4.1: Funnel plot of global improvement or treatment success (primary analysis)**


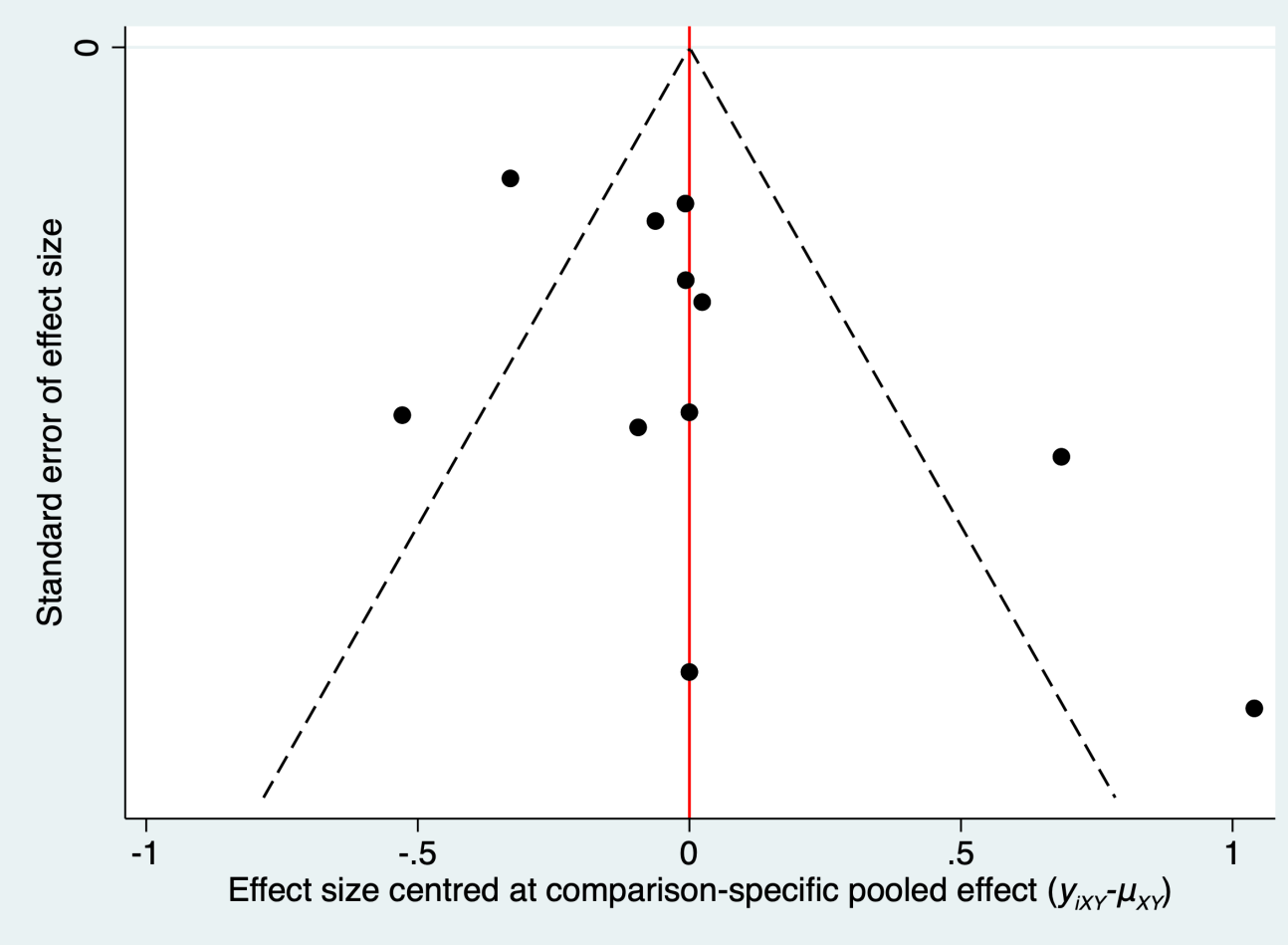


**Figure S4.2: Funnel plot of complete resolution of pain (primary analysis)**


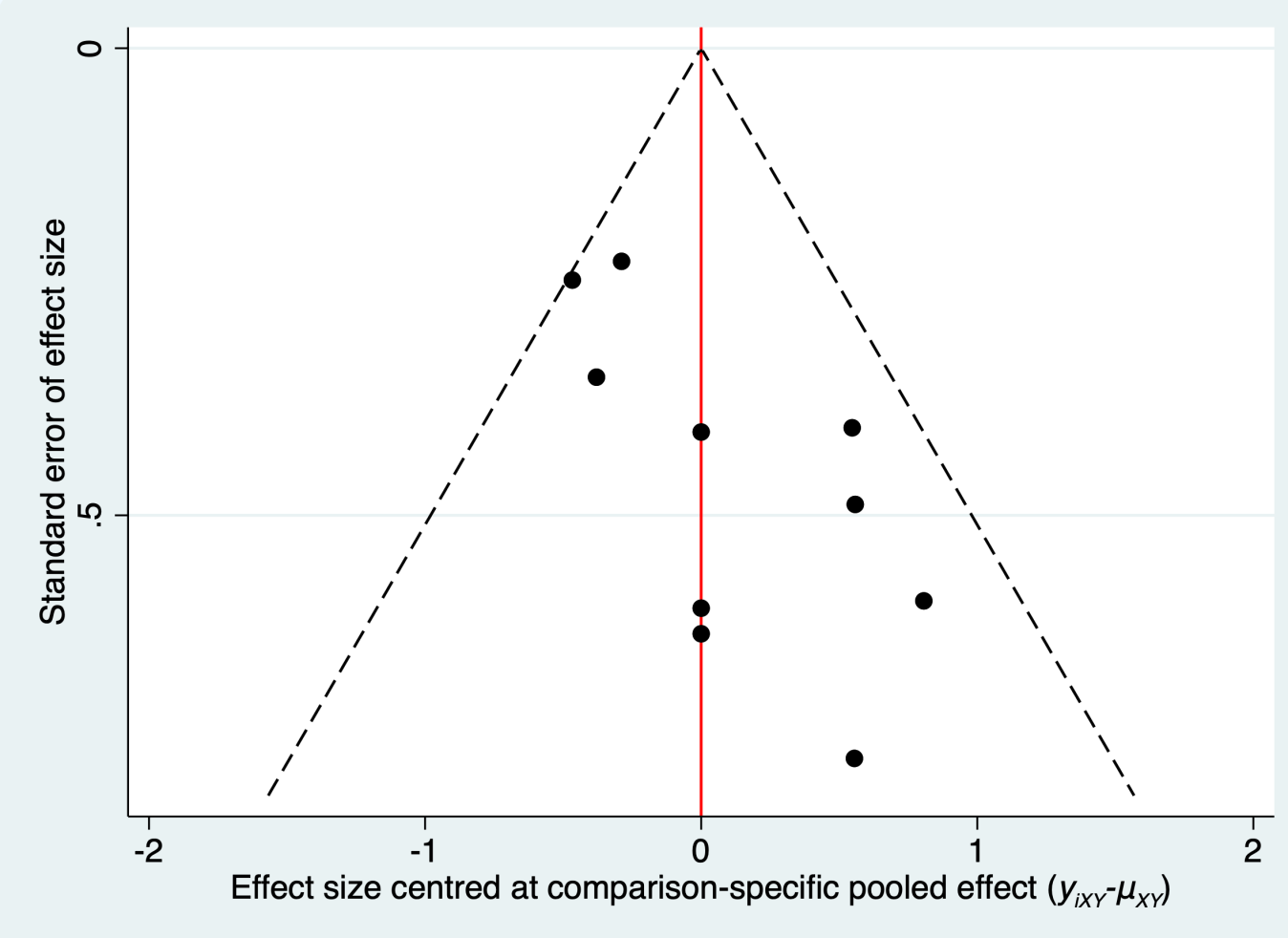


**Figure S4.3: Funnel plot of severity of pain (primary analysis)**


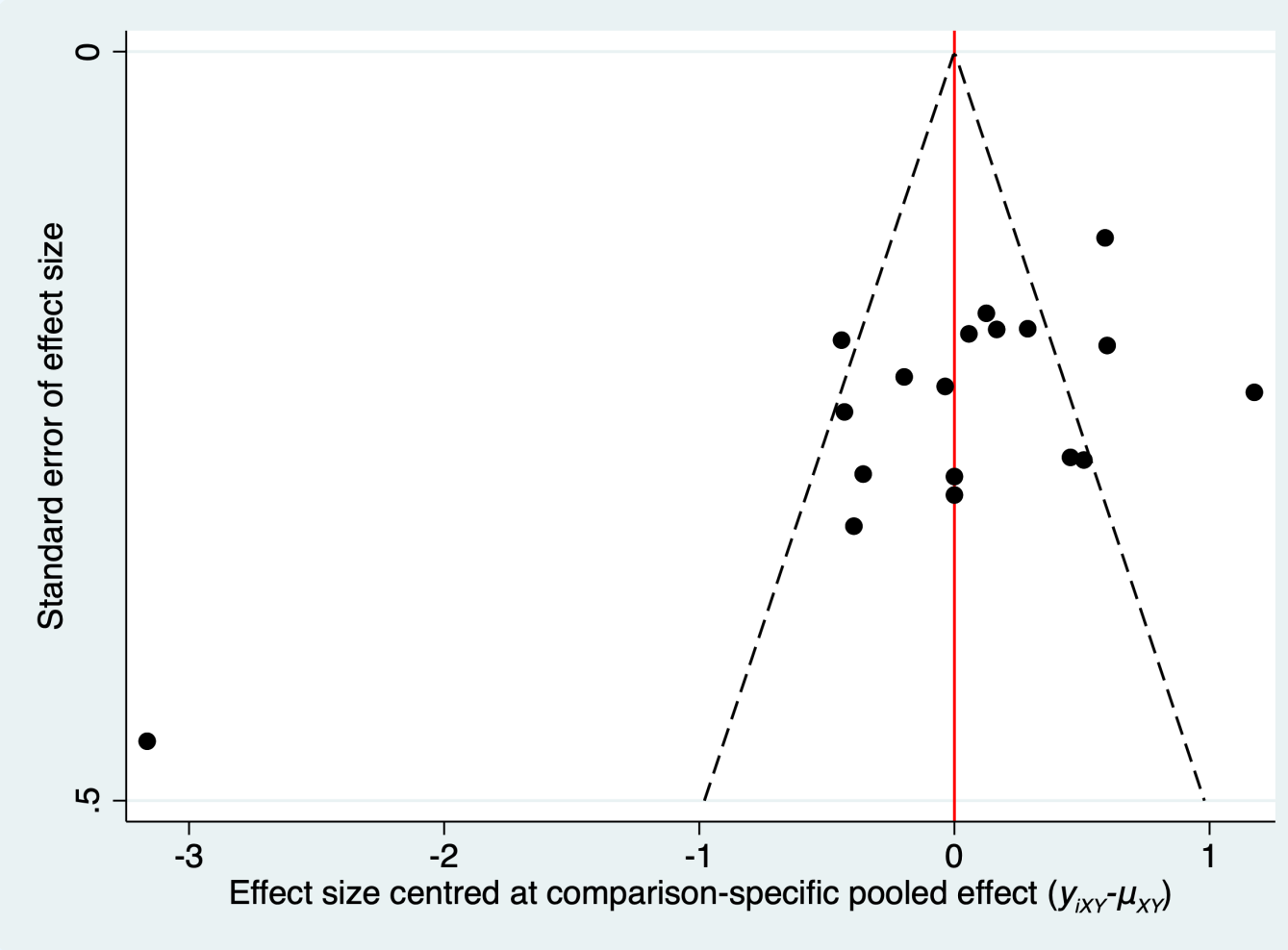


**Figure S4.4: Funnel plot of frequency of pain (primary analysis)**


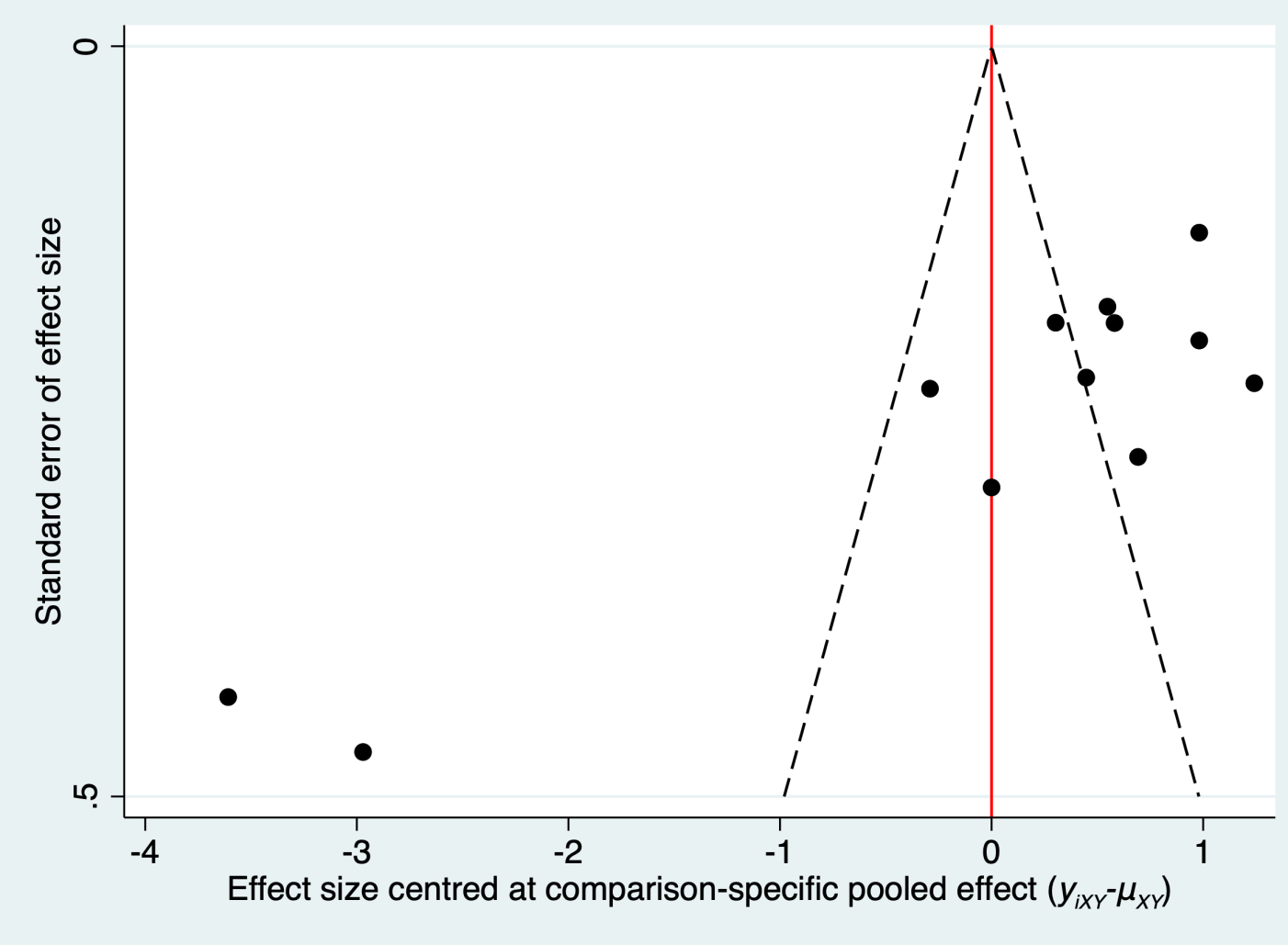


**Figure S4.5: Funnel plot of global improvement or treatment success (subgroup analysis)**


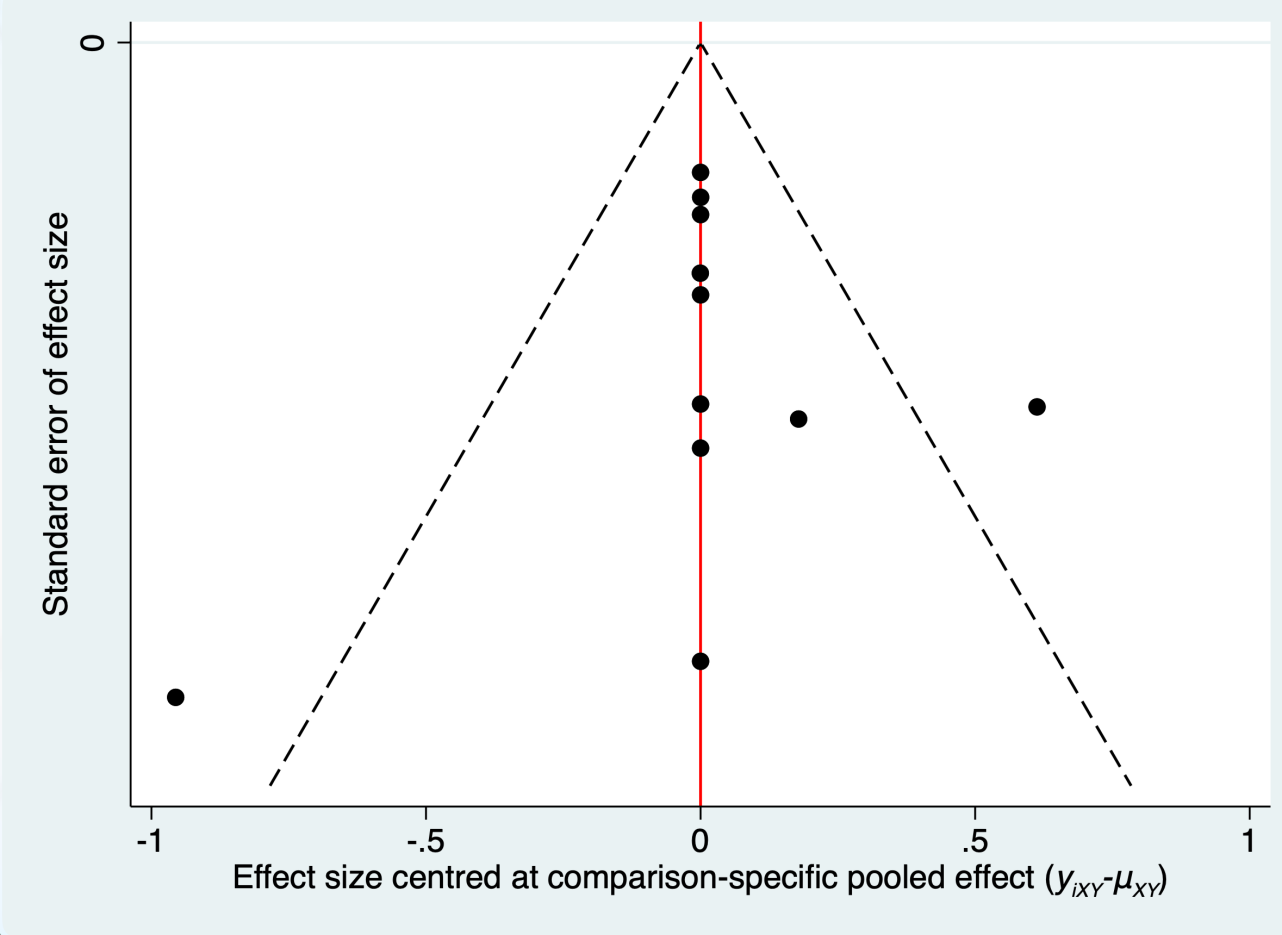


**Figure S4.6: Funnel plot of complete resolution of pain (subgroup analysis)**


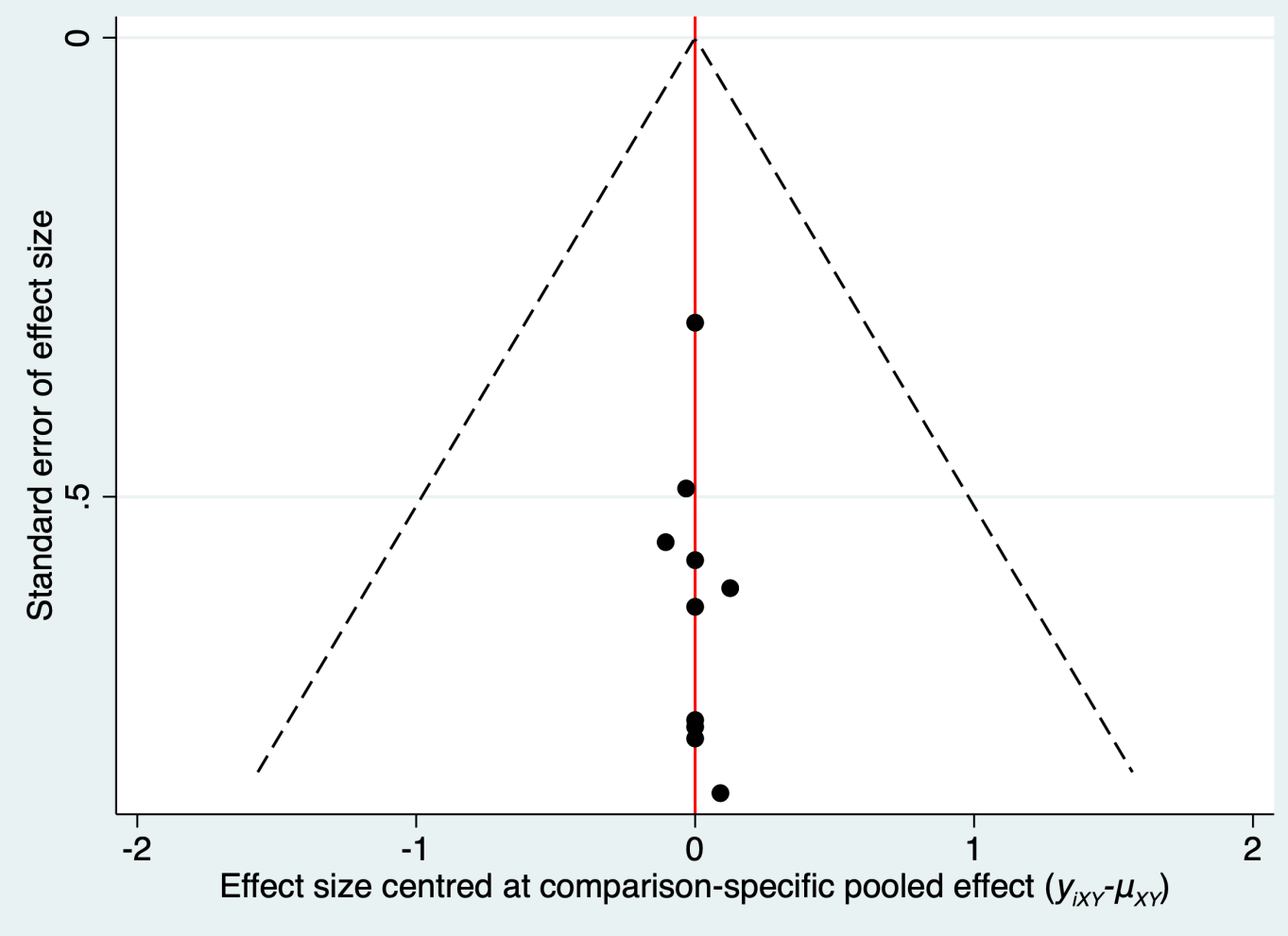


**Figure S4.7: Funnel plot of severity of pain (subgroup analysis)**


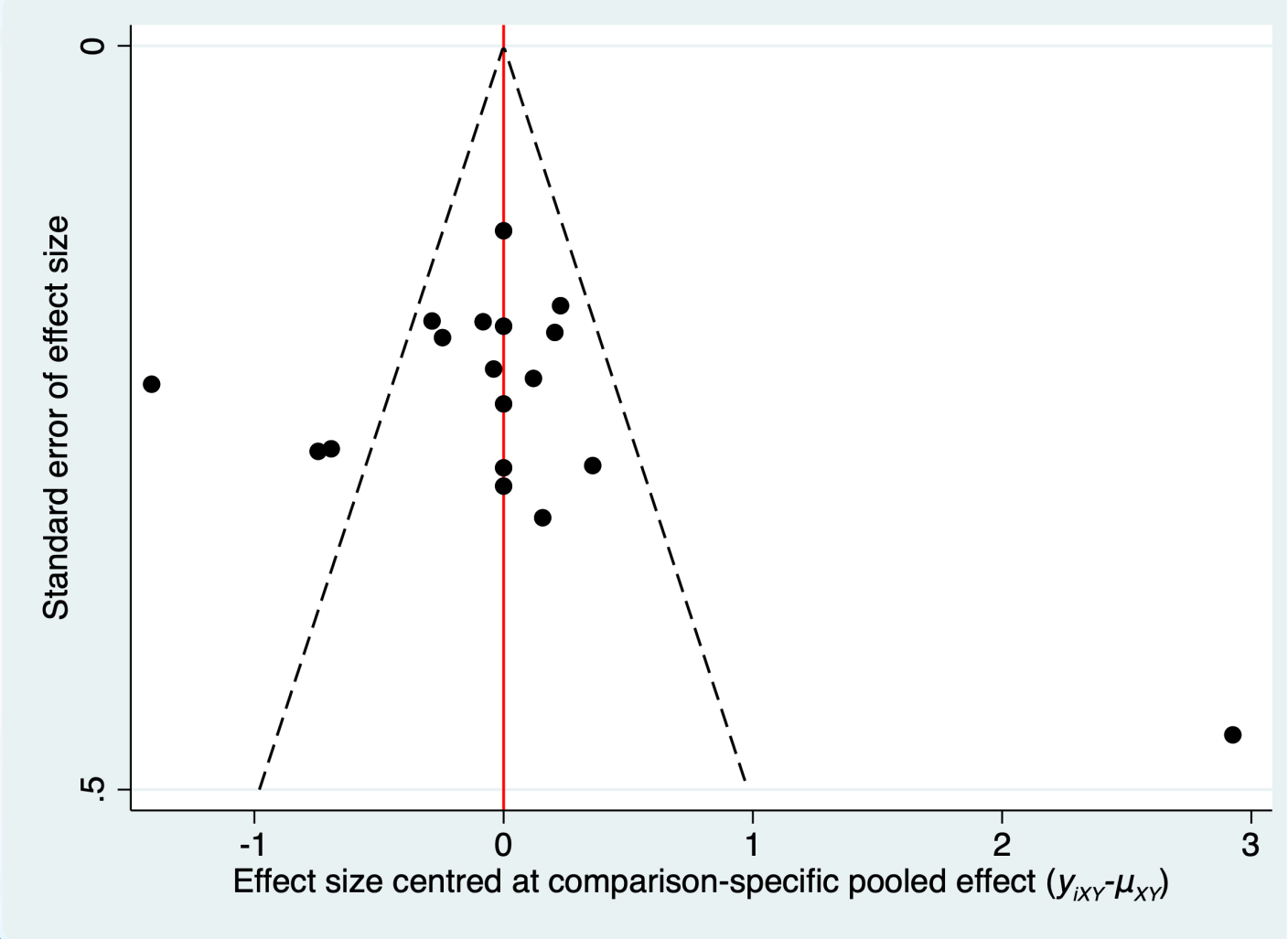


**Figure S4.8: Funnel plot of frequency of pain (subgroup analysis)**


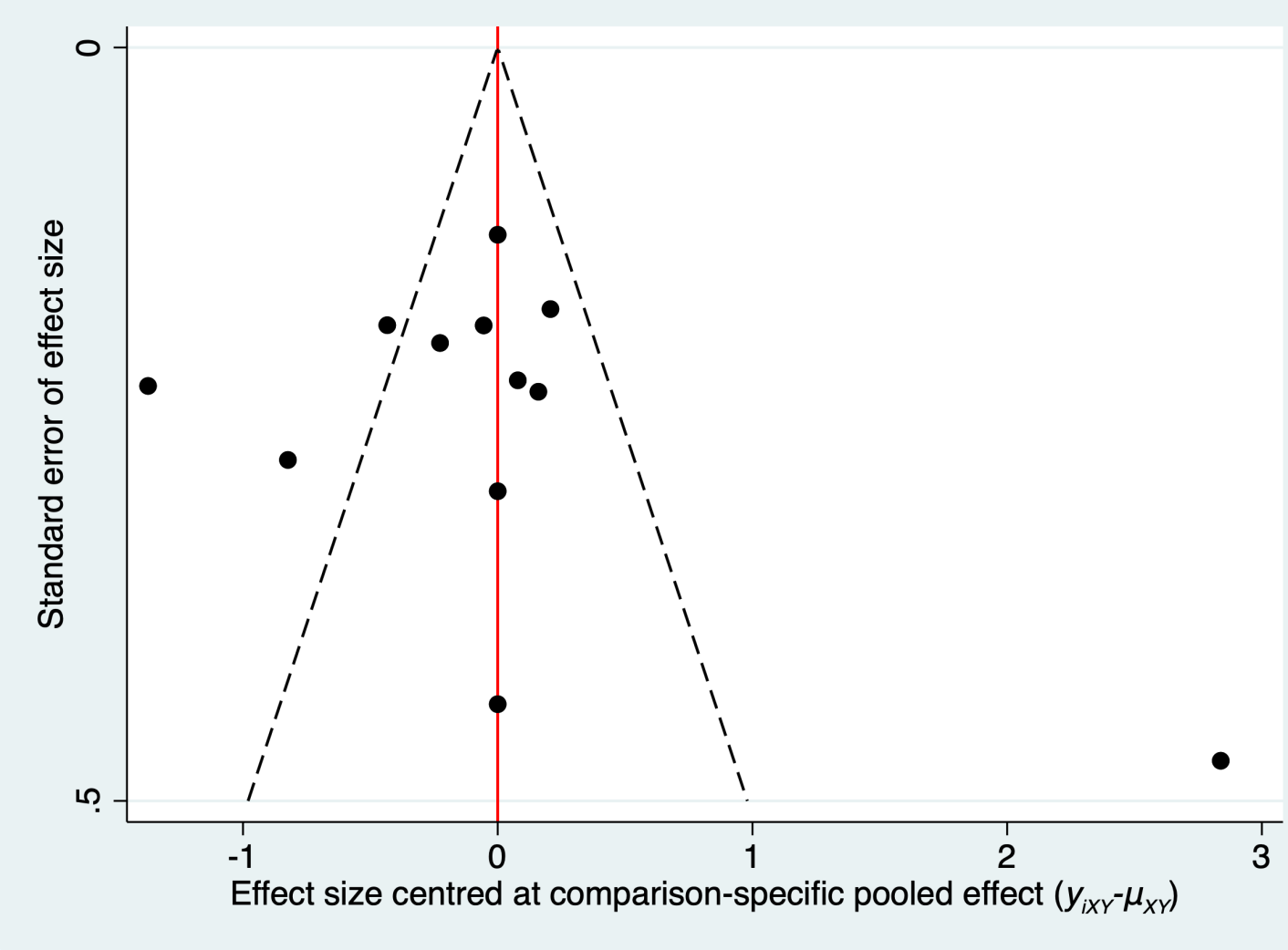


**Appendix 5: CINeMA Assessment**

**We use the CINeMA framework to evidence certainty, assessing it for each network estimate based on the following criteria:**

**A: Within study bias:** We classified the overall risk of bias for each study as low risk of bias, the risk of bias as moderate when none of the four assessed risk of bias items were rated as high risk, and the risk of bias as high when one or both items were rated as high risk. See Appendix 4 for the bias assessment. The risk of bias for a pairwise comparison of each drug is shown in figure S5.1-5.16.

**Global improvement or treatment success**

**Figure S5.1. Risk of bias contribution by intervention group (main analysis)**

**
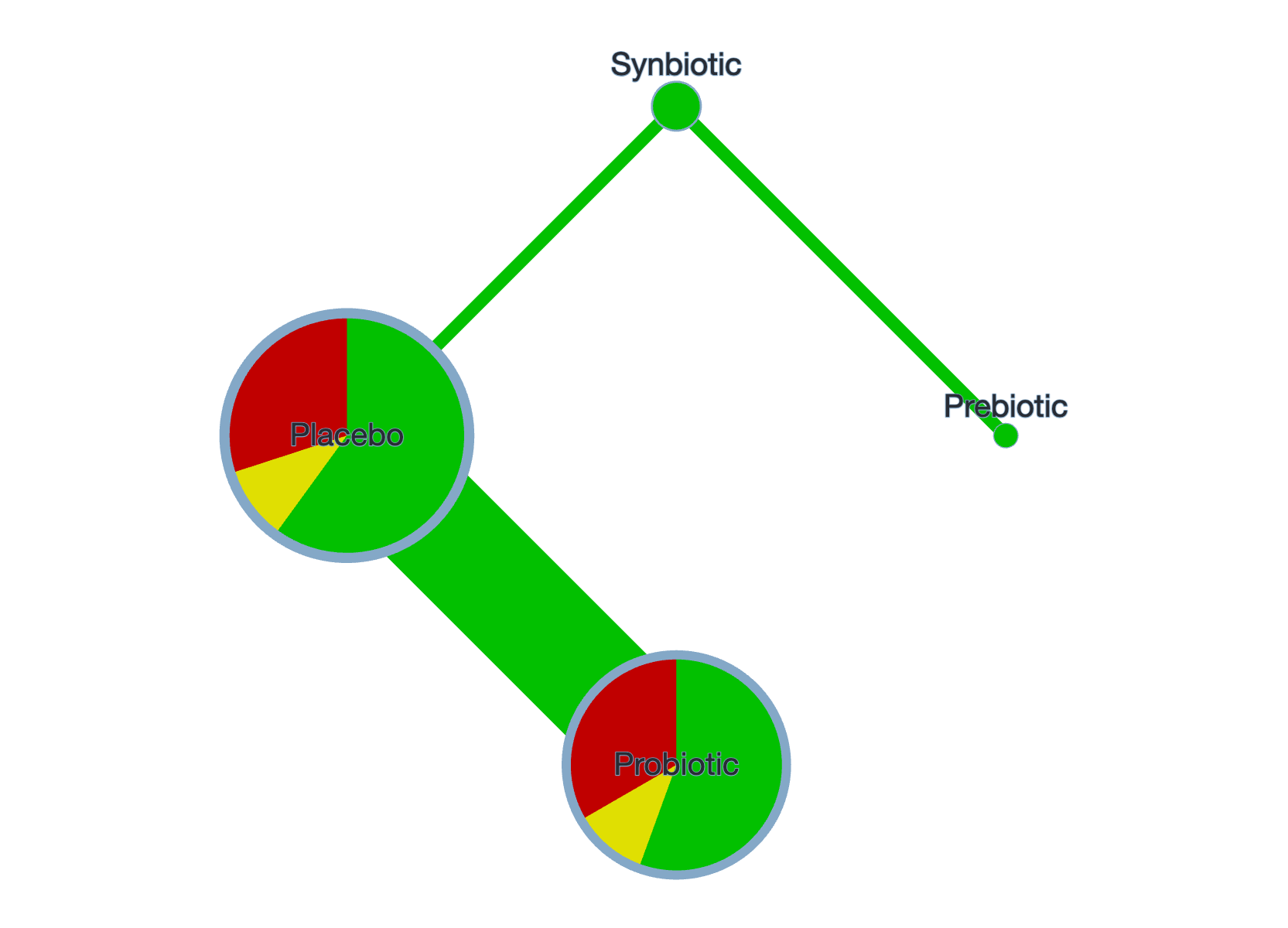
**

**Figure S5.2. Overall risk of bias by treatment comparison (main analysis)**

**
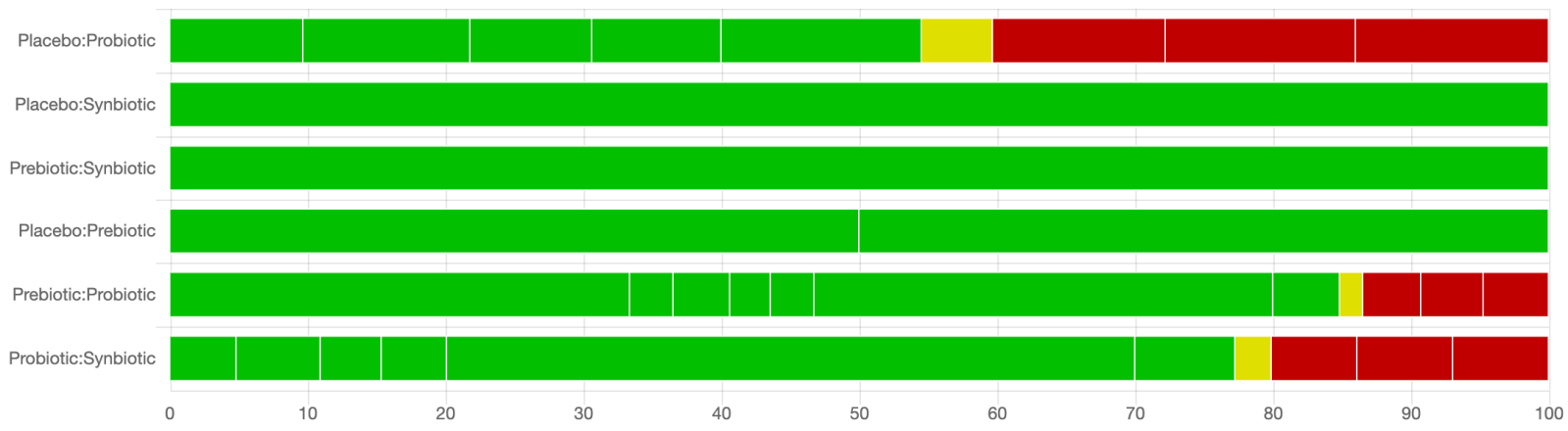
**

**Figure S5.3. Risk of bias contribution by intervention group (subgroup analysis)**

**
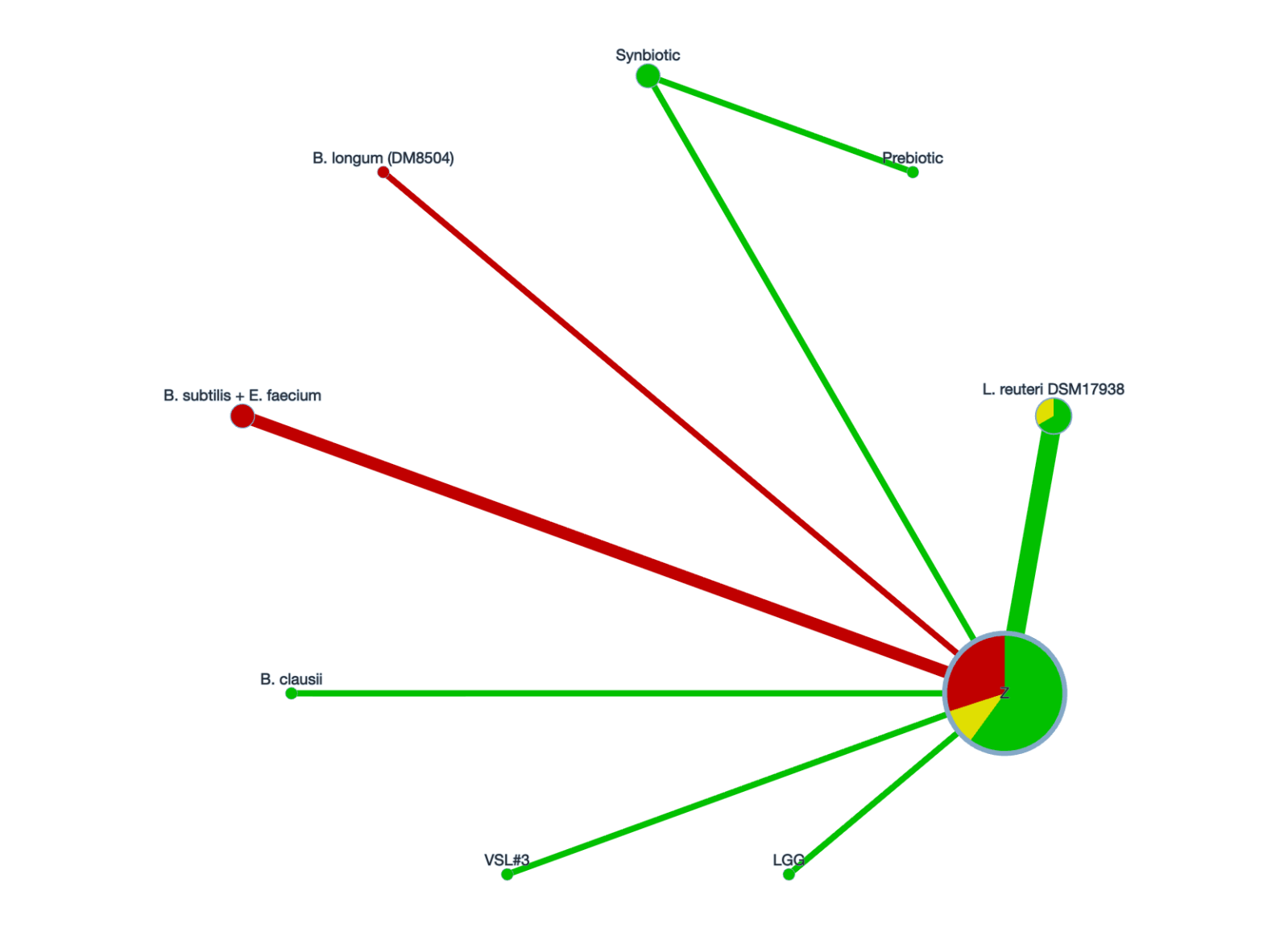
**

**Figure S5.4. Overall risk of bias by treatment comparison (subgroup analysis)**

**
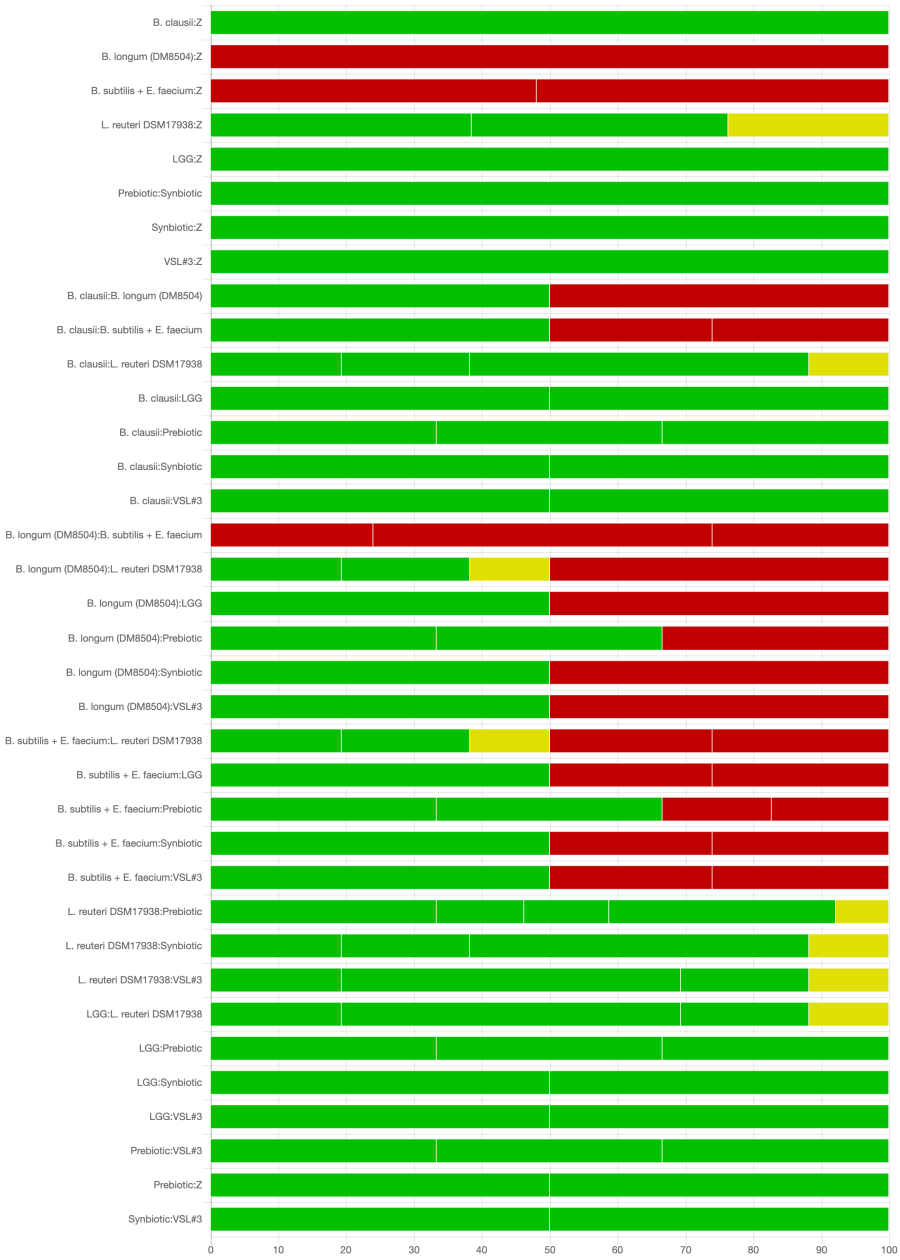
**

**Complete resolution of pain**

**Figure S5.5. Risk of bias contribution by intervention group (main analysis)**

**
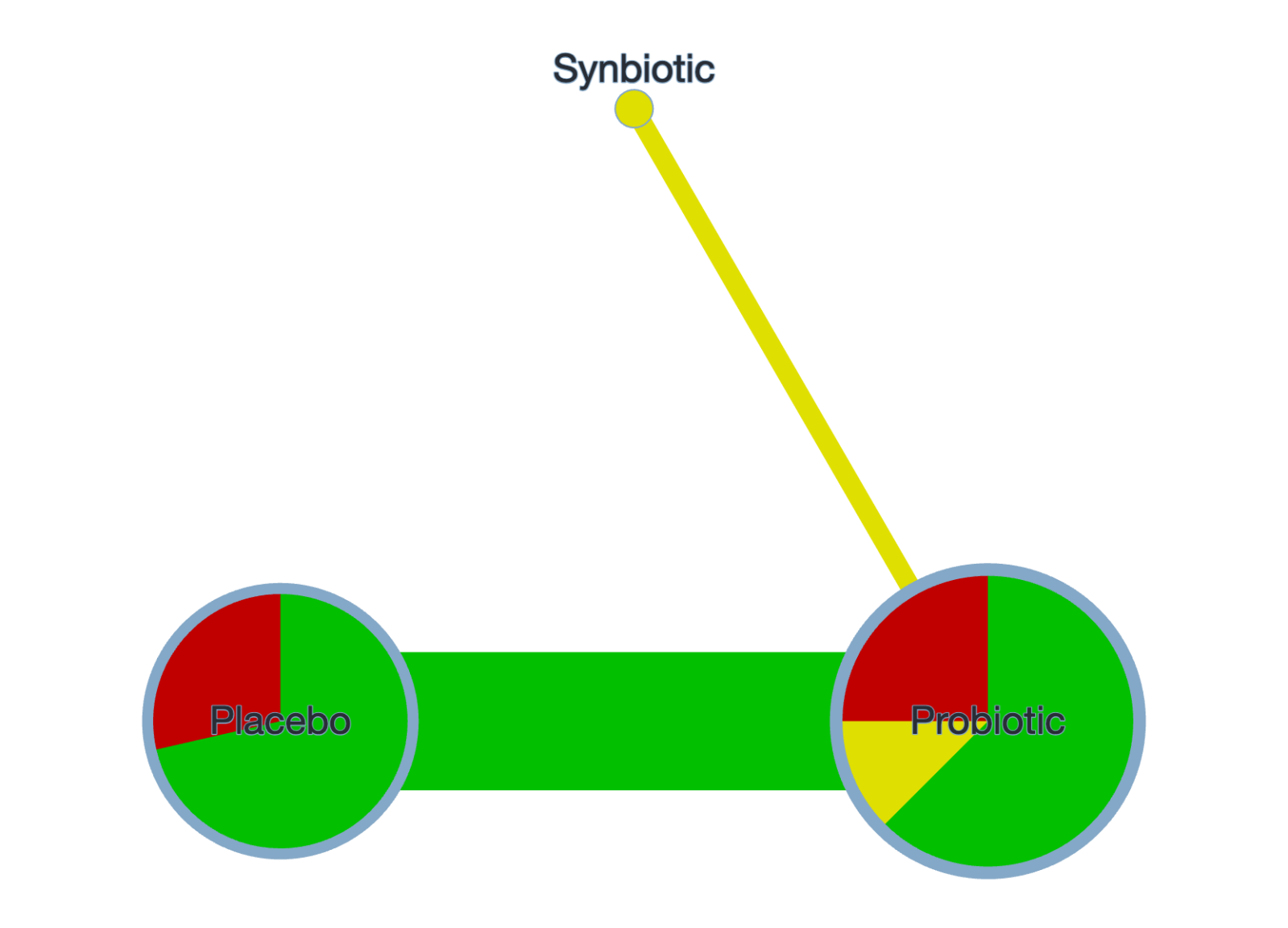
**

**Figure S5.6. Overall risk of bias by treatment comparison (main analysis)**

**
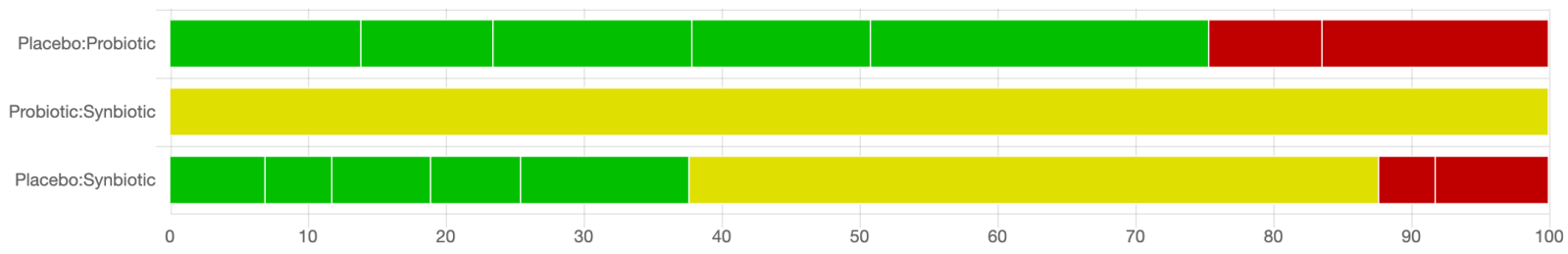
**

**Figure S5.7. Risk of bias contribution by intervention group (subgroup analysis)**

**
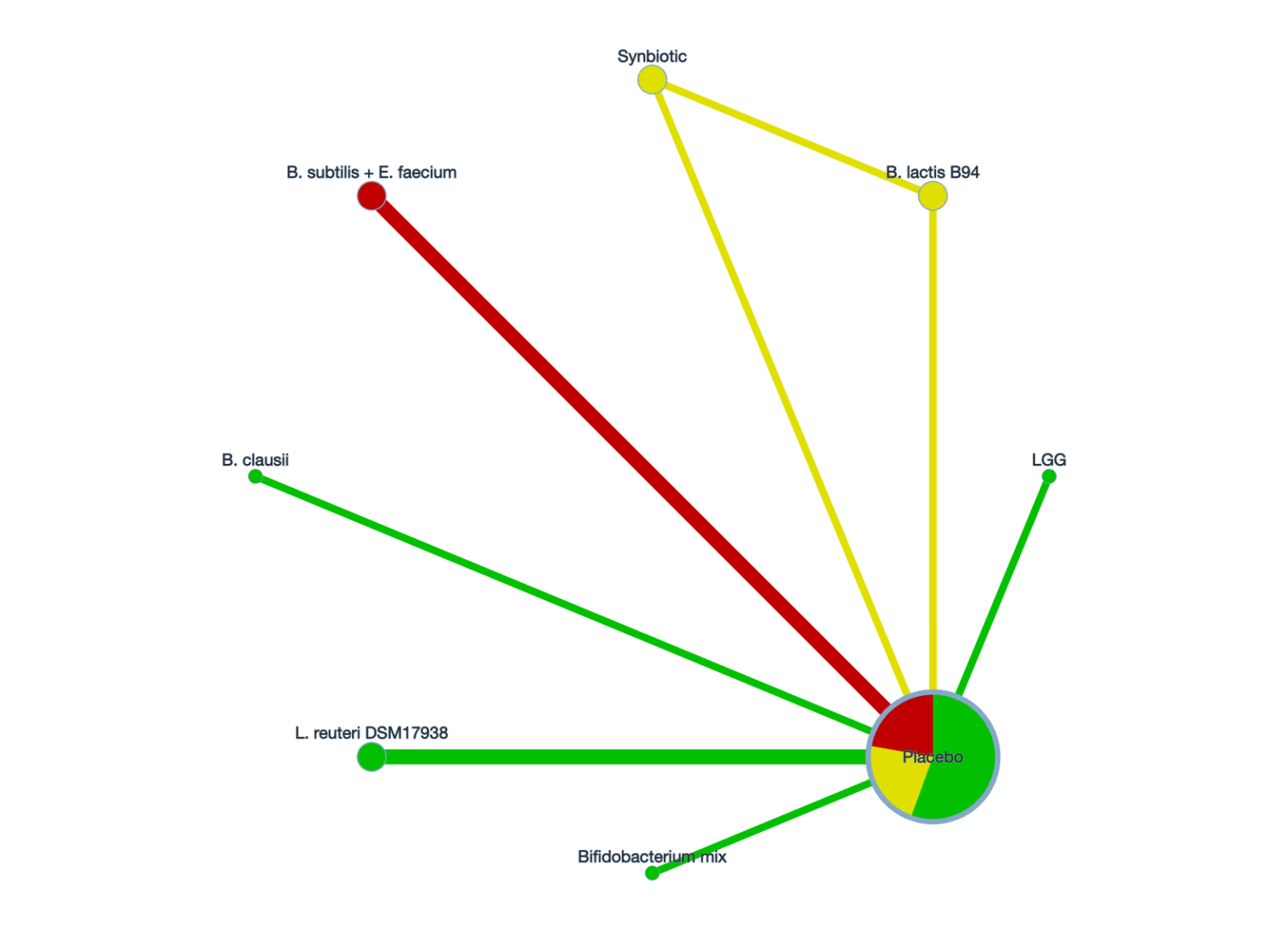
**

**Figure S5.8. Overall risk of bias by treatment comparison (subgroup analysis)**

**
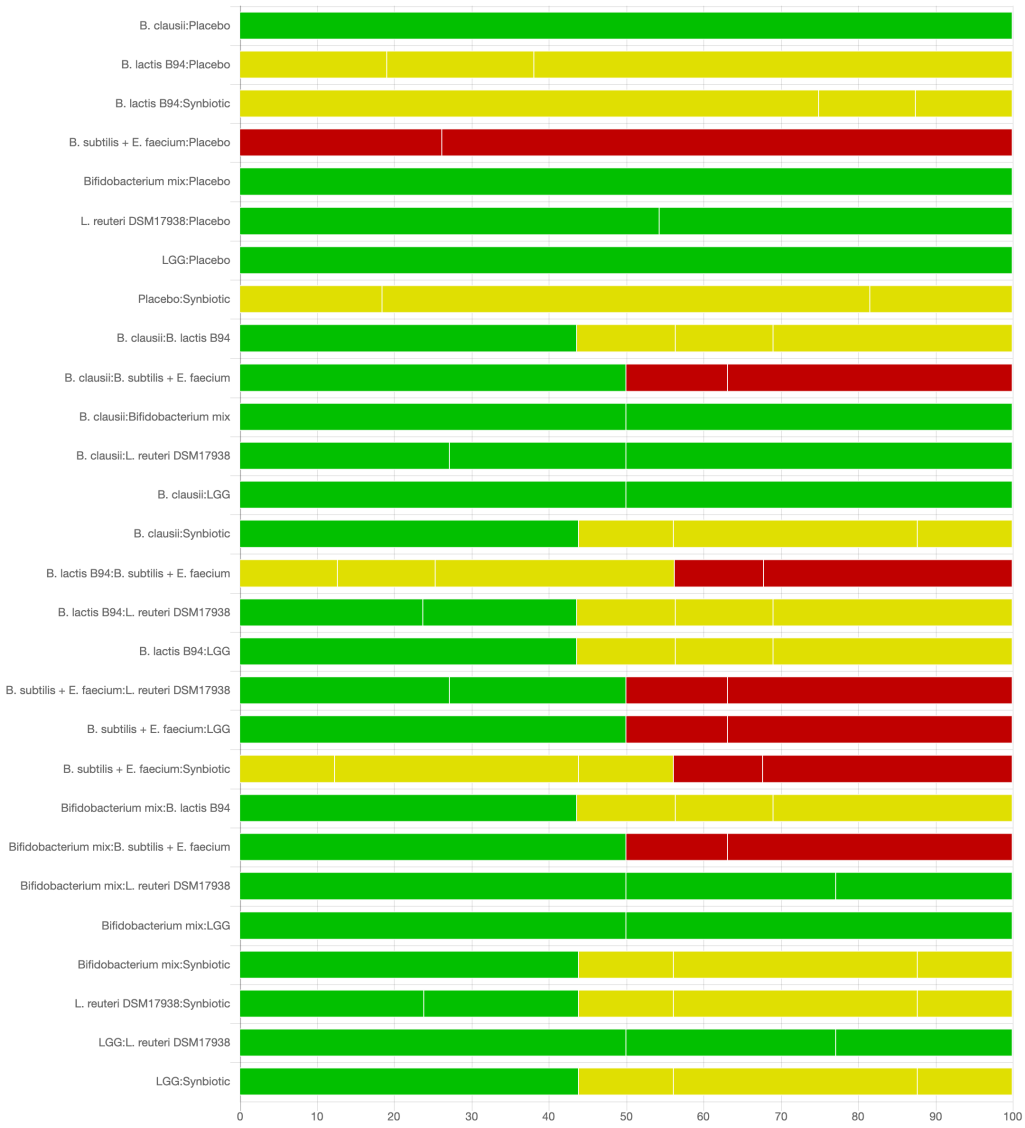
**

**Severity of pain**

**Figure S5.9. Risk of bias contribution by intervention group (main analysis)**

**
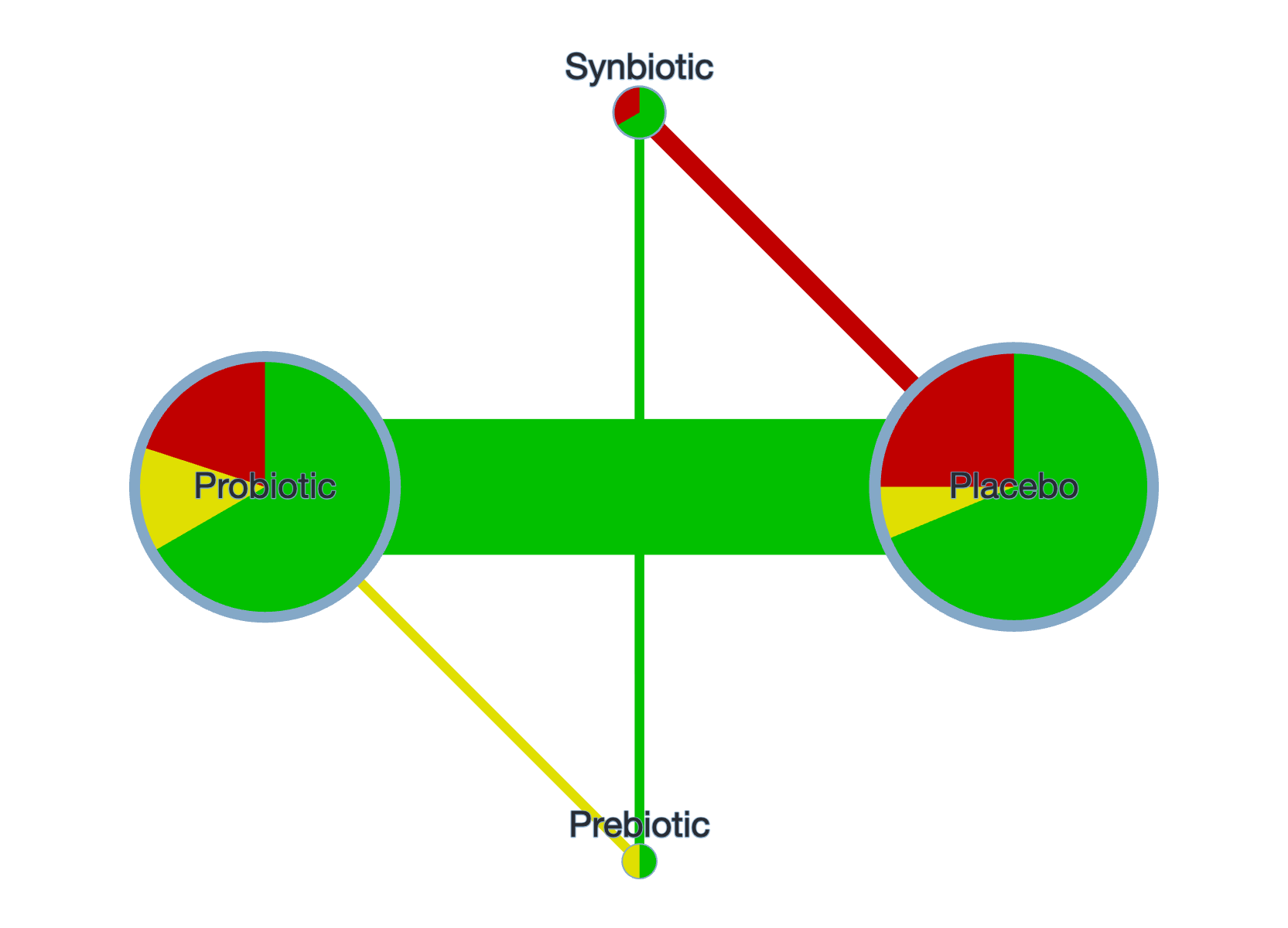
**

**Figure S5.10. Overall risk of bias by treatment comparison (main analysis)**

**
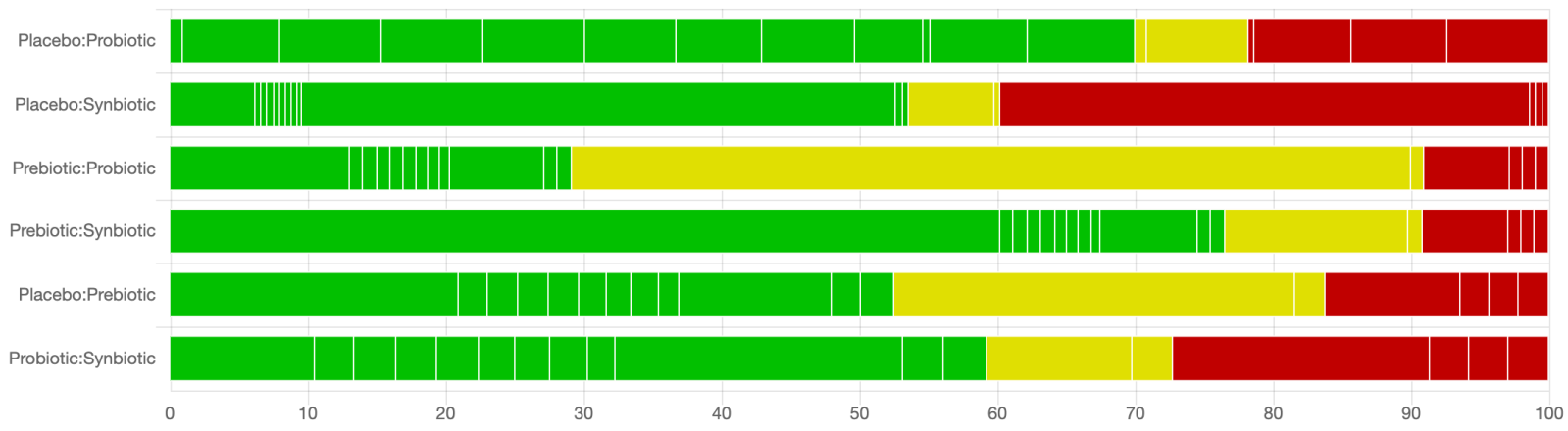
**

**Figure S5.11. Risk of bias contribution by intervention group (subgroup analysis)**

**
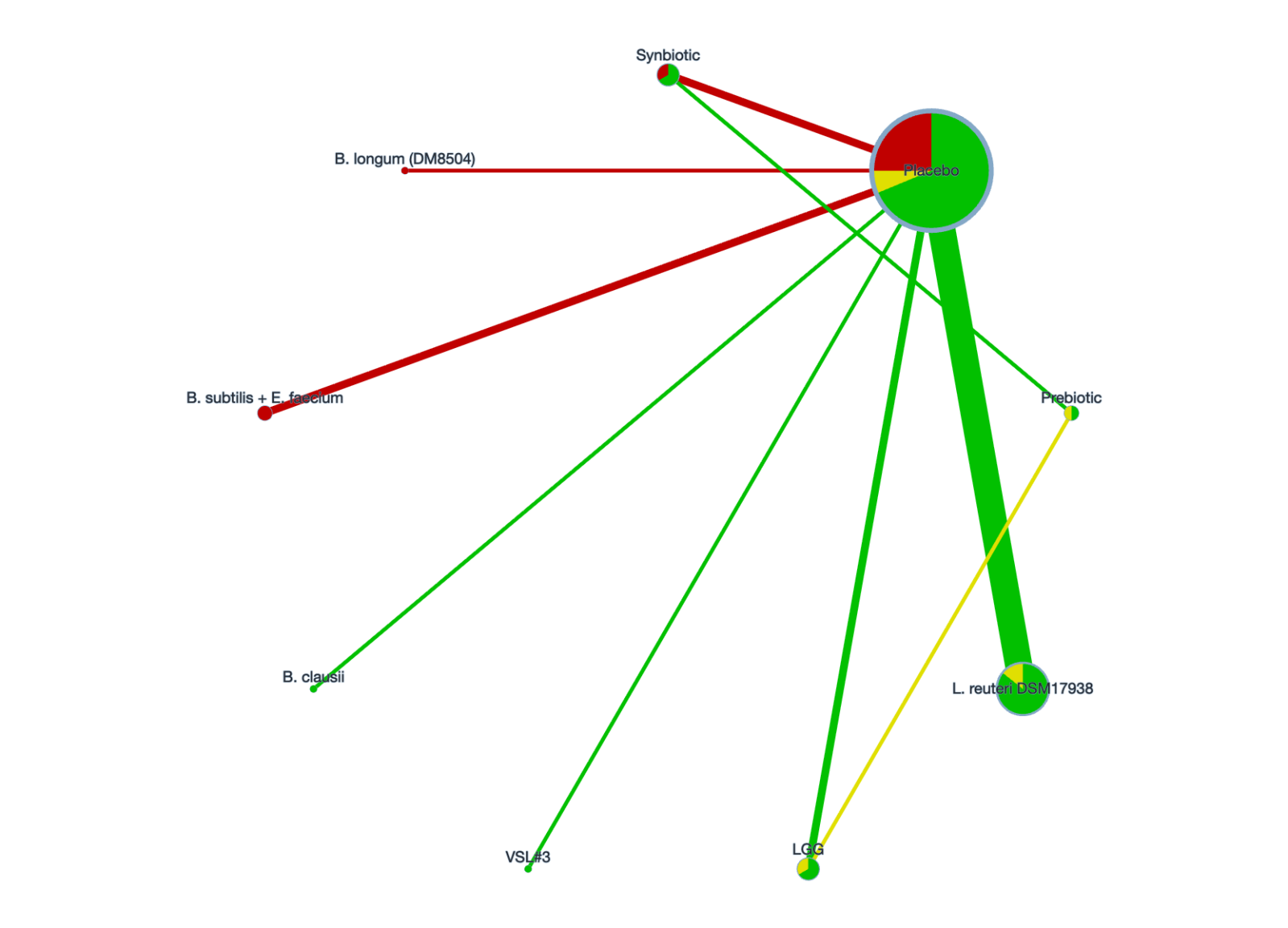
**

**Figure S5.12. Overall risk of bias by treatment comparison (subgroup analysis)**

**
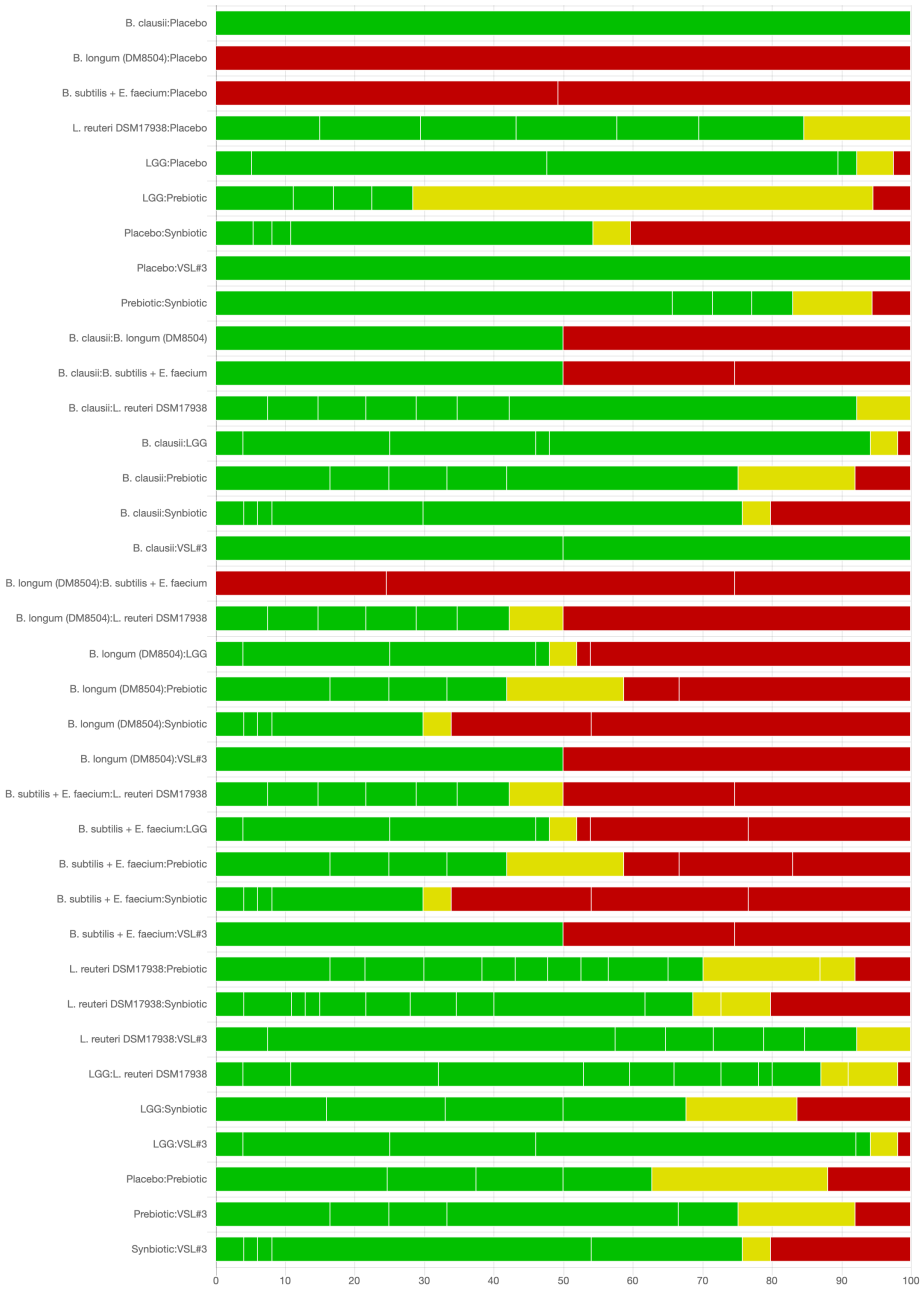
**

**Frequency of pain**

**Figure S5.13. Risk of bias contribution by intervention group (main analysis)**

**
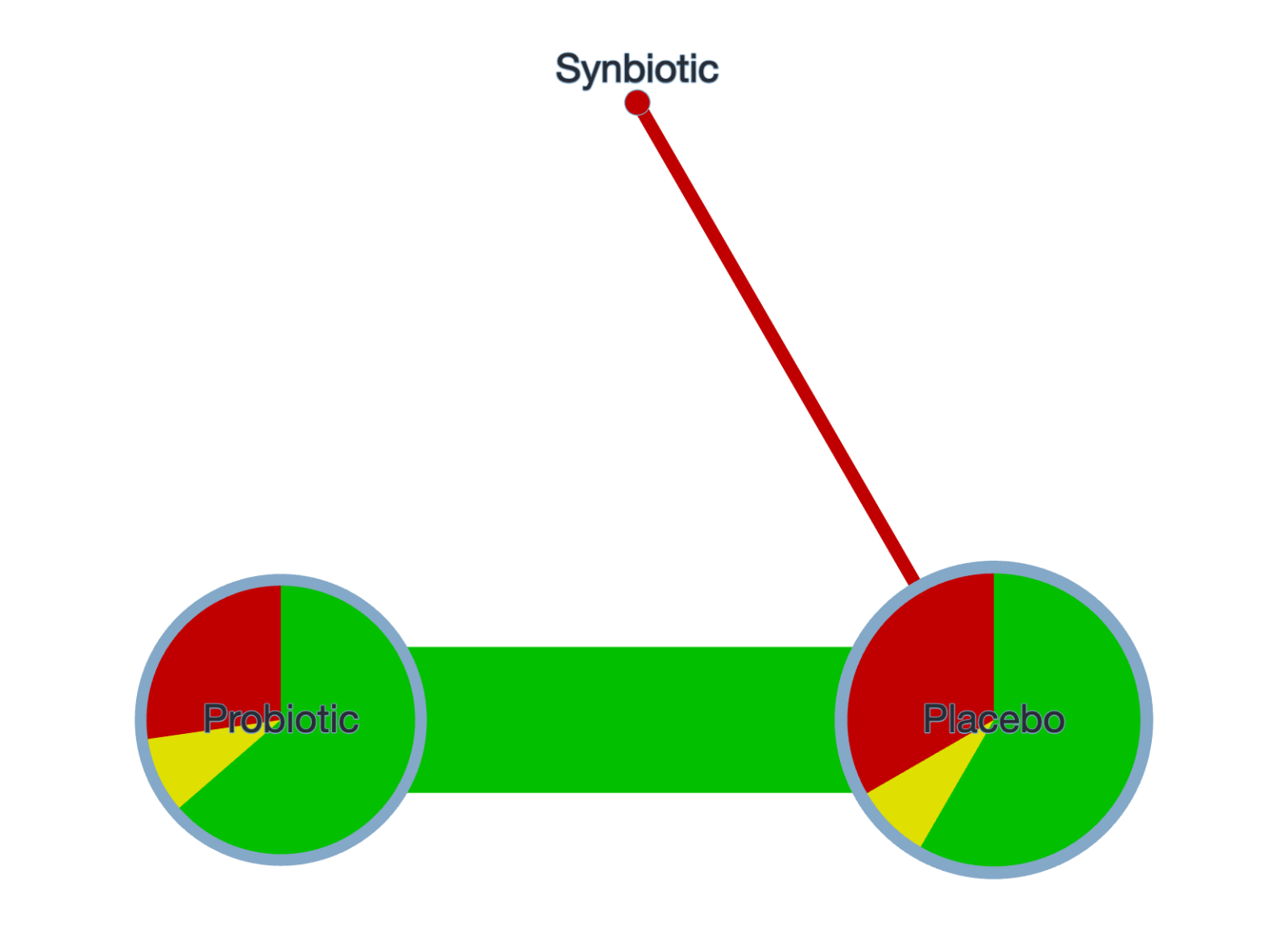
**

**Figure S5.14. Overall risk of bias by treatment comparison (main analysis)**

**
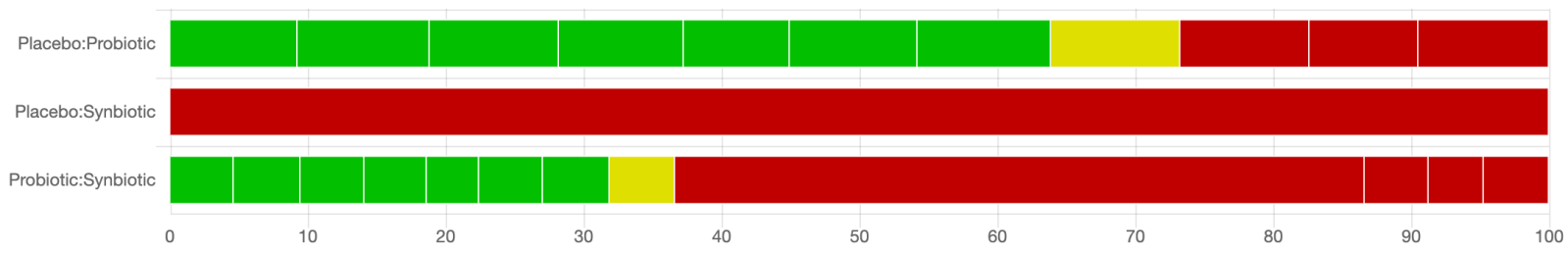
**

**Figure S5.15. Risk of bias contribution by intervention group (subgroup analysis)**

**
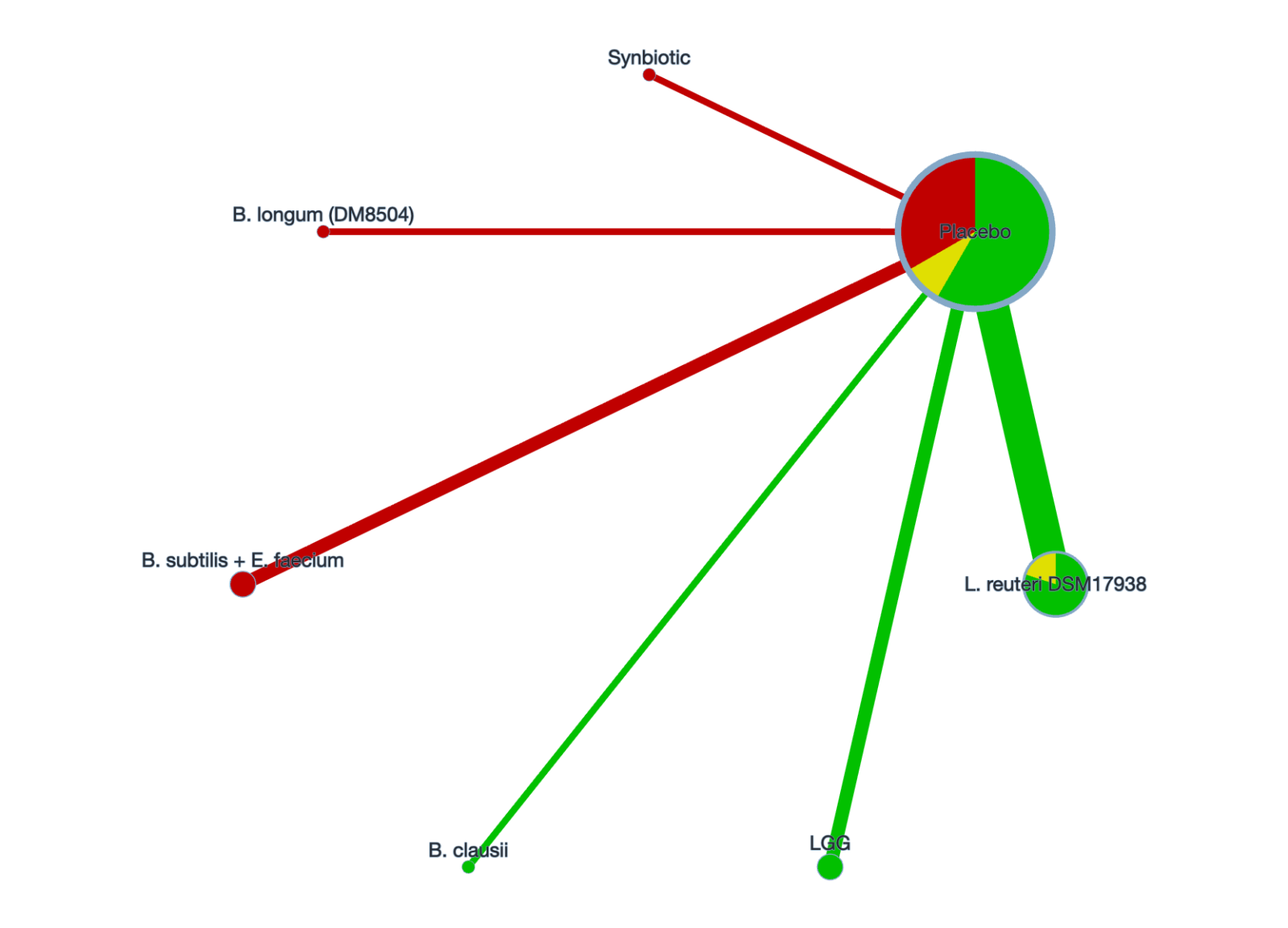
**

**Figure S5.16. Overall risk of bias by treatment comparison (subgroup analysis)**

**
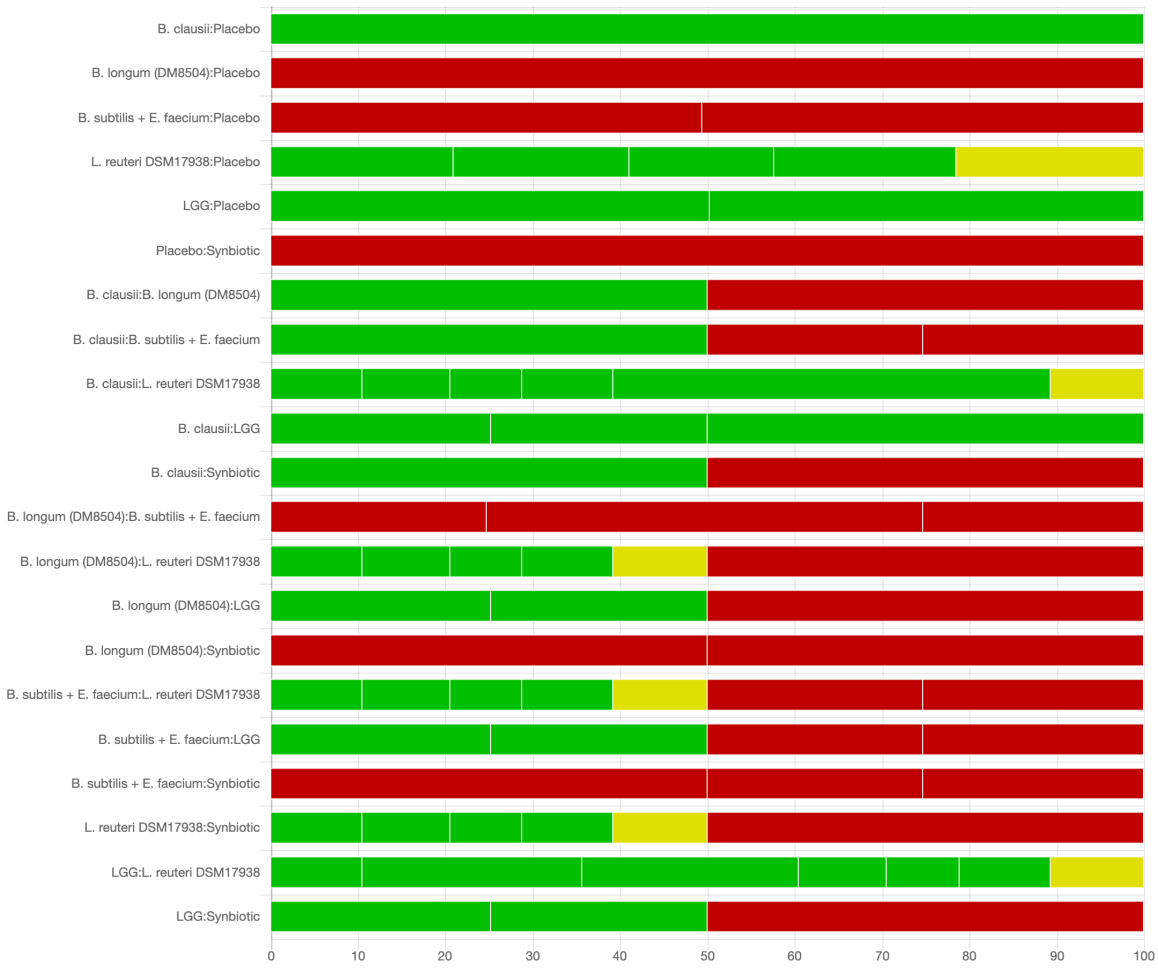
**

**B: Reporting bias:** We judged it visually by a funnel plot **(Appendix 4)**.

**C: Indirectness:** Indirectness: Transferability assumptions were evaluated based on the baseline characteristics of the included studies, as summarized in the study characteristics table.

**D: Imprecision:** We use the CINeMA website to grade the accuracy of each comparison.

**E: Heterogeneity:** We assessed the degree of worry by comparing clinical reasoning based on 95% confidence intervals (CIs) while applying the same clinical reasoning framework as for inaccuracy. In particular, we judged the consistency of our findings based on the confidence and prediction intervals associated with clinically important effect sizes. And we used the same thresholds of clinical significance as described above and followed the recommendations automatically provided by CINeMA (https://cinema.ispm.unibe.ch/).

**F: Inconsistency:** For inconsistency, we looked at the results for node splitting (Appendix 5) and we saw major problems when p<0.10, but otherwise no problems

**Table S5.1: CINeMA assessment results for Global improvement or treatment success (main analysis)**

| Comparison | Within-study bias | Reporting bias | Indirectness | Imprecision | Heterogeneity | Incoherence | Confidence rating |
| --- | --- | --- | --- | --- | --- | --- | --- |
| Placebo:Probiotic | No concerns | Low risk | No concerns | No concerns | No concerns | Major concerns | Moderate |
| Placebo:Synbiotic | No concerns | Low risk | No concerns | Major concerns | No concerns | Major concerns | Low |
| Prebiotic:Synbiotic | No concerns | Low risk | No concerns | Major concerns | No concerns | Major concerns | Low |
| Placebo:Prebiotic | No concerns | Low risk | No concerns | Major concerns | No concerns | Major concerns | Low |
| Prebiotic:Probiotic | No concerns | Low risk | No concerns | Major concerns | No concerns | Major concerns | Low |
| Probiotic:Synbiotic | No concerns | Low risk | No concerns | Major concerns | No concerns | Major concerns | Low |

**Table S5.2: CINeMA assessment results for Global improvement or treatment success (subgroup analysis)**

| Comparison | Within-study bias | Reporting bias | Indirectness | Imprecision | Heterogeneity | Incoherence | Confidence rating |
| --- | --- | --- | --- | --- | --- | --- | --- |
| B. clausii:Z | No concerns | Low risk | No concerns | Major concerns | No concerns | Major concerns | Low |
| B. longum (DM8504):Z | Major concerns | Low risk | No concerns | Major concerns | No concerns | Major concerns | Low |
| B. subtilis + E. faecium:Z | Major concerns | Low risk | No concerns | Major concerns | No concerns | Major concerns | Low |
| L. reuteri DSM17938:Z | No concerns | Low risk | No concerns | Major concerns | No concerns | Major concerns | Low |
| LGG:Z | No concerns | Low risk | No concerns | Major concerns | No concerns | Major concerns | Low |
| Prebiotic:Synbiotic | No concerns | Low risk | No concerns | Major concerns | No concerns | Major concerns | Low |
| Synbiotic:Z | No concerns | Low risk | No concerns | Major concerns | No concerns | Major concerns | Low |

**Table S5.3: CINeMA assessment results for Complete resolution of pain (main analysis)**

| Comparison | Within-study bias | Reporting bias | Indirectness | Imprecision | Heterogeneity | Incoherence | Confidence rating |
| --- | --- | --- | --- | --- | --- | --- | --- |
| Placebo:Probiotic | No concerns | Low risk | No concerns | No concerns | Major concerns | Major concerns | Low |
| Probiotic:Synbiotic | Some concerns | Low risk | No concerns | Major concerns | No concerns | Major concerns | Low |
| Placebo:Synbiotic | Some concerns | Low risk | No concerns | Major concerns | No concerns | Major concerns | Low |

**Table S5.4: CINeMA assessment results for Complete resolution of pain (subgroup analysis)**

| Comparison | Within-study bias | Reporting bias | Indirectness | Imprecision | Heterogeneity | Incoherence | Confidence rating |
| --- | --- | --- | --- | --- | --- | --- | --- |
| B. clausii:Placebo | No concerns | Low risk | No concerns | Major concerns | No concerns | No concerns | Moderate |
| B. lactis B94:Placebo | Some concerns | Low risk | No concerns | Major concerns | No concerns | No concerns | Moderate |
| B. lactis B94:Synbiotic | Some concerns | Low risk | No concerns | Major concerns | No concerns | No concerns | Moderate |
| B. subtilis + E. faecium:Placebo | Major concerns | Low risk | No concerns | No concerns | Major concerns | No concerns | Low |
| Bifidobacterium mix:Placebo | No concerns | Low risk | No concerns | No concerns | Major concerns | No concerns | Low |
| L. reuteri DSM17938:Placebo | No concerns | Low risk | No concerns | Major concerns | No concerns | No concerns | Moderate |
| LGG:Placebo | No concerns | Low risk | No concerns | No concerns | Major concerns | No concerns | Moderate |
| Placebo:Synbiotic | Some concerns | Low risk | No concerns | No concerns | Major concerns | No concerns | Moderate |
| B. clausii:B. lactis B94 | Some concerns | Low risk | No concerns | Major concerns | No concerns | No concerns | Moderate |
| B. clausii:B. subtilis + E. faecium | No concerns | Low risk | No concerns | No concerns | Major concerns | No concerns | Moderate |
| B. clausii:Bifidobacterium mix | No concerns | Low risk | No concerns | No concerns | Major concerns | No concerns | Moderate |
| B. clausii:L. reuteri DSM17938 | No concerns | Low risk | No concerns | Major concerns | No concerns | No concerns | Moderate |
| B. clausii:LGG | No concerns | Low risk | No concerns | Major concerns | No concerns | No concerns | Moderate |
| B. clausii:Synbiotic | Some concerns | Low risk | No concerns | No concerns | Major concerns | No concerns | Moderate |
| B. lactis B94:B. subtilis + E. faecium | Some concerns | Low risk | No concerns | Major concerns | No concerns | No concerns | Moderate |
| Bifidobacterium mix:B. lactis B94 | Some concerns | Low risk | No concerns | Major concerns | No concerns | No concerns | Moderate |
| B. lactis B94:L. reuteri DSM17938 | Some concerns | Low risk | No concerns | Major concerns | No concerns | No concerns | Moderate |
| B. lactis B94:LGG | Some concerns | Low risk | No concerns | Major concerns | No concerns | No concerns | Moderate |
| Bifidobacterium mix:B. subtilis + E. faecium | No concerns | Low risk | No concerns | Major concerns | No concerns | No concerns | Moderate |
| B. subtilis + E. faecium:L. reuteri DSM17938 | No concerns | Low risk | No concerns | Major concerns | No concerns | No concerns | Moderate |
| B. subtilis + E. faecium:LGG | No concerns | Low risk | No concerns | Major concerns | No concerns | No concerns | Moderate |
| B. subtilis + E. faecium:Synbiotic | Some concerns | Low risk | No concerns | Major concerns | No concerns | No concerns | Moderate |
| Bifidobacterium mix:L. reuteri DSM17938 | No concerns | Low risk | No concerns | Major concerns | No concerns | No concerns | Moderate |
| Bifidobacterium mix:LGG | No concerns | Low risk | No concerns | Major concerns | No concerns | No concerns | Moderate |
| Bifidobacterium mix:Synbiotic | Some concerns | Low risk | No concerns | Major concerns | No concerns | No concerns | Moderate |
| LGG:L. reuteri DSM17938 | No concerns | Low risk | No concerns | Major concerns | No concerns | No concerns | Moderate |
| L. reuteri DSM17938:Synbiotic | Some concerns | Low risk | No concerns | Major concerns | No concerns | No concerns | Moderate |
| LGG:Synbiotic | Some concerns | Low risk | No concerns | Major concerns | No concerns | No concerns | Moderate |

**Table S5.5: CINeMA assessment results for Severity of pain (main analysis)**

| Comparison | Within-study bias | Reporting bias | Indirectness | Imprecision | Heterogeneity | Incoherence | Confidence rating |
| --- | --- | --- | --- | --- | --- | --- | --- |
| Placebo:Probiotic | No concerns | Low risk | No concerns | No concerns | Major concerns | No concerns | Moderate |
| Placebo:Synbiotic | No concerns | Low risk | No concerns | Major concerns | No concerns | No concerns | Moderate |
| Prebiotic:Probiotic | Some concerns | Low risk | No concerns | Major concerns | No concerns | No concerns | Moderate |
| Prebiotic:Synbiotic | No concerns | Low risk | No concerns | Major concerns | No concerns | No concerns | Moderate |
| Placebo:Prebiotic | No concerns | Low risk | No concerns | Major concerns | No concerns | No concerns | Moderate |
| Probiotic:Synbiotic | No concerns | Low risk | No concerns | Major concerns | No concerns | No concerns | Moderate |

**Table S5.6: CINeMA assessment results for Severity of pain (subgroup analysis)**

| Comparison | Within-study bias | Reporting bias | Indirectness | Imprecision | Heterogeneity | Incoherence | Confidence rating |
| --- | --- | --- | --- | --- | --- | --- | --- |
| B. clausii:Placebo | No concerns | Low risk | No concerns | Major concerns | No concerns | No concerns | Moderate |
| B. longum (DM8504):Placebo | Major concerns | Low risk | No concerns | Major concerns | No concerns | No concerns | Low |
| B. subtilis + E. faecium:Placebo | Major concerns | Low risk | No concerns | Major concerns | No concerns | No concerns | Low |
| L. reuteri DSM17938:Placebo | No concerns | Low risk | No concerns | No concerns | Major concerns | No concerns | Moderate |
| LGG:Placebo | No concerns | Low risk | No concerns | Major concerns | No concerns | No concerns | Moderate |
| LGG:Prebiotic | Some concerns | Low risk | No concerns | Major concerns | No concerns | No concerns | Moderate |
| Placebo:Synbiotic | No concerns | Low risk | No concerns | Major concerns | No concerns | No concerns | Moderate |

**Table S5.7: CINeMA assessment results for Frequency of pain (main analysis)**

| Comparison | Within-study bias | Reporting bias | Indirectness | Imprecision | Heterogeneity | Incoherence | Confidence rating |
| --- | --- | --- | --- | --- | --- | --- | --- |
| Placebo:Probiotic | No concerns | Low risk | No concerns | No concerns | Major concerns | Major concerns | Low |
| Placebo:Synbiotic | Major concerns | Low risk | No concerns | Major concerns | No concerns | Major concerns | Low |
| Probiotic:Synbiotic | Major concerns | Low risk | No concerns | Major concerns | No concerns | Major concerns | Low |

**Table S5.8: CINeMA assessment results for Frequency of pain (subgroup analysis)**

| Comparison | Within-study bias | Reporting bias | Indirectness | Imprecision | Heterogeneity | Incoherence | Confidence rating |
| --- | --- | --- | --- | --- | --- | --- | --- |
| B. clausii:Placebo | No concerns | Low risk | No concerns | Major concerns | No concerns | Major concerns | Low |
| B. longum (DM8504):Placebo | Major concerns | Low risk | No concerns | No concerns | No concerns | Major concerns | Low |
| B. subtilis + E. faecium:Placebo | Major concerns | Low risk | No concerns | Major concerns | No concerns | Major concerns | Low |
| L. reuteri DSM17938:Placebo | No concerns | Low risk | No concerns | No concerns | Major concerns | Major concerns | Low |
| LGG:Placebo | No concerns | Low risk | No concerns | Major concerns | No concerns | Major concerns | Low |
| Placebo:Synbiotic | Major concerns | Low risk | No concerns | Major concerns | No concerns | Major concerns | Low |
| B. clausii:B. longum (DM8504) | No concerns | Low risk | No concerns | No concerns | No concerns | Major concerns | Moderate |
| B. clausii:B. subtilis + E. faecium | No concerns | Low risk | No concerns | Major concerns | No concerns | Major concerns | Low |
| B. clausii:L. reuteri DSM17938 | No concerns | Low risk | No concerns | Major concerns | No concerns | Major concerns | Low |
| B. clausii:LGG | No concerns | Low risk | No concerns | Major concerns | No concerns | Major concerns | Low |
| B. clausii:Synbiotic | No concerns | Low risk | No concerns | Major concerns | No concerns | Major concerns | Low |
| B. longum (DM8504):B. subtilis + E. faecium | Major concerns | Low risk | No concerns | No concerns | No concerns | Major concerns | Low |
| B. longum (DM8504):L. reuteri DSM17938 | Major concerns | Low risk | No concerns | No concerns | No concerns | Major concerns | Low |
| B. longum (DM8504):LGG | No concerns | Low risk | No concerns | No concerns | No concerns | Major concerns | Low |
| B. longum (DM8504):Synbiotic | Major concerns | Low risk | No concerns | No concerns | Major concerns | Major concerns | Low |
| B. subtilis + E. faecium:L. reuteri DSM17938 | Major concerns | Low risk | No concerns | Major concerns | No concerns | Major concerns | Low |
| B. subtilis + E. faecium:LGG | No concerns | Low risk | No concerns | Major concerns | No concerns | Major concerns | Low |
| B. subtilis + E. faecium:Synbiotic | Major concerns | Low risk | No concerns | Major concerns | No concerns | Major concerns | Low |
| LGG:L. reuteri DSM17938 | No concerns | Low risk | No concerns | Major concerns | No concerns | Major concerns | Low |
| L. reuteri DSM17938:Synbiotic | Major concerns | Low risk | No concerns | Major concerns | No concerns | Major concerns | Low |
| LGG:Synbiotic | No concerns | Low risk | No concerns | Major concerns | No concerns | Major concerns | Low |

**Appendix 6: SUCRA and cumulative probability plots**

**Figure S6.1: SUCRA and cumulative probability plots for global improvement or treatment success (primary analysis)**


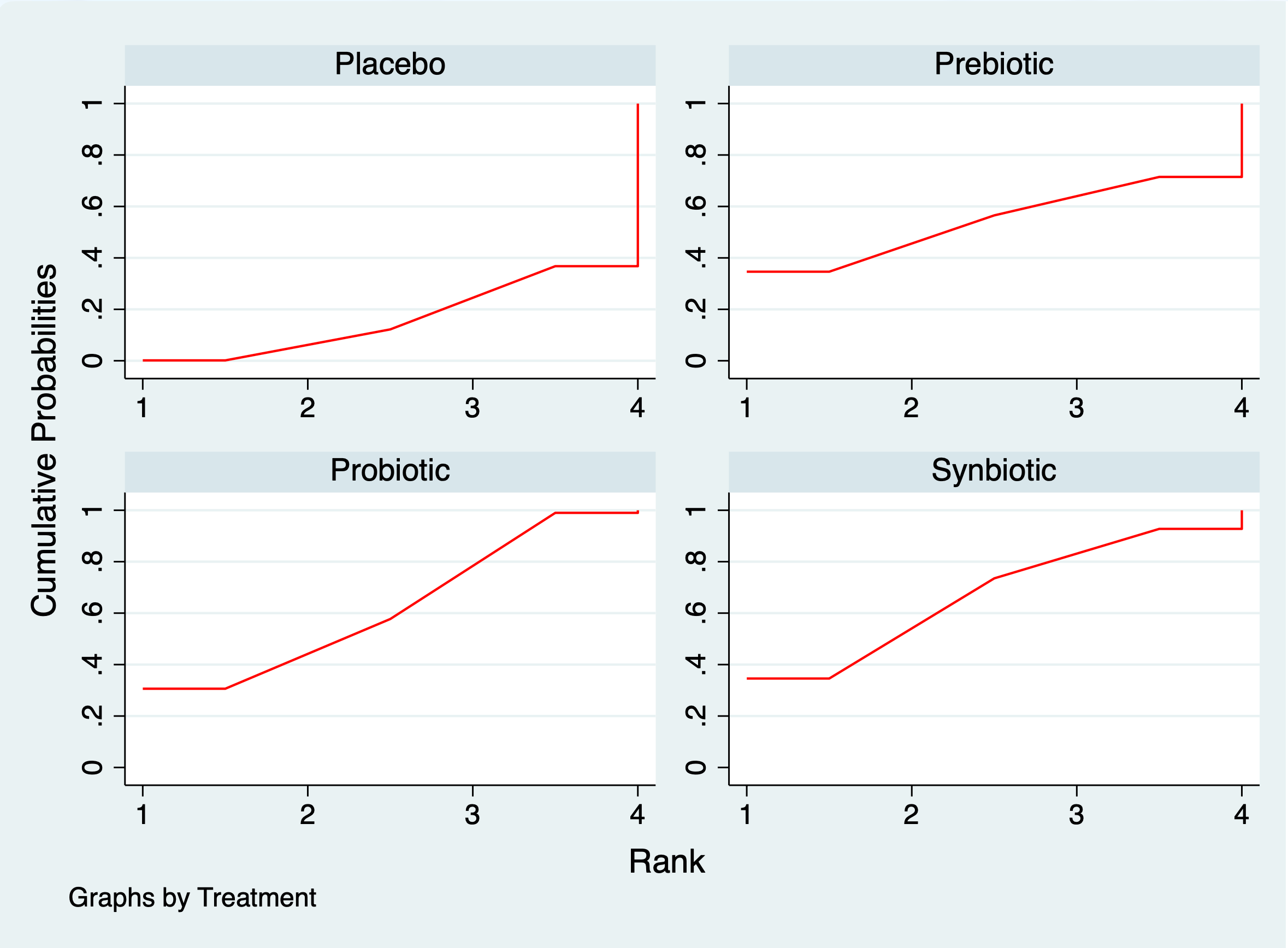


| **Treatmentt** | **SUCRA** | **PrBest** | **MeanRank** |
| --- | --- | --- | --- |
| **Placebo** | **16.4** | **0.2** | **3.5** |
| **Prebiotic** | **54.2** | **34.6** | **2.4** |
| **Probiotic** | **62.4** | **30.6** | **2.1** |
| **Synbiotic** | **67** | **34.6** | **2** |

**Figure S6.2: SUCRA and cumulative probability plots for complete resolution of pain (primary analysis)**


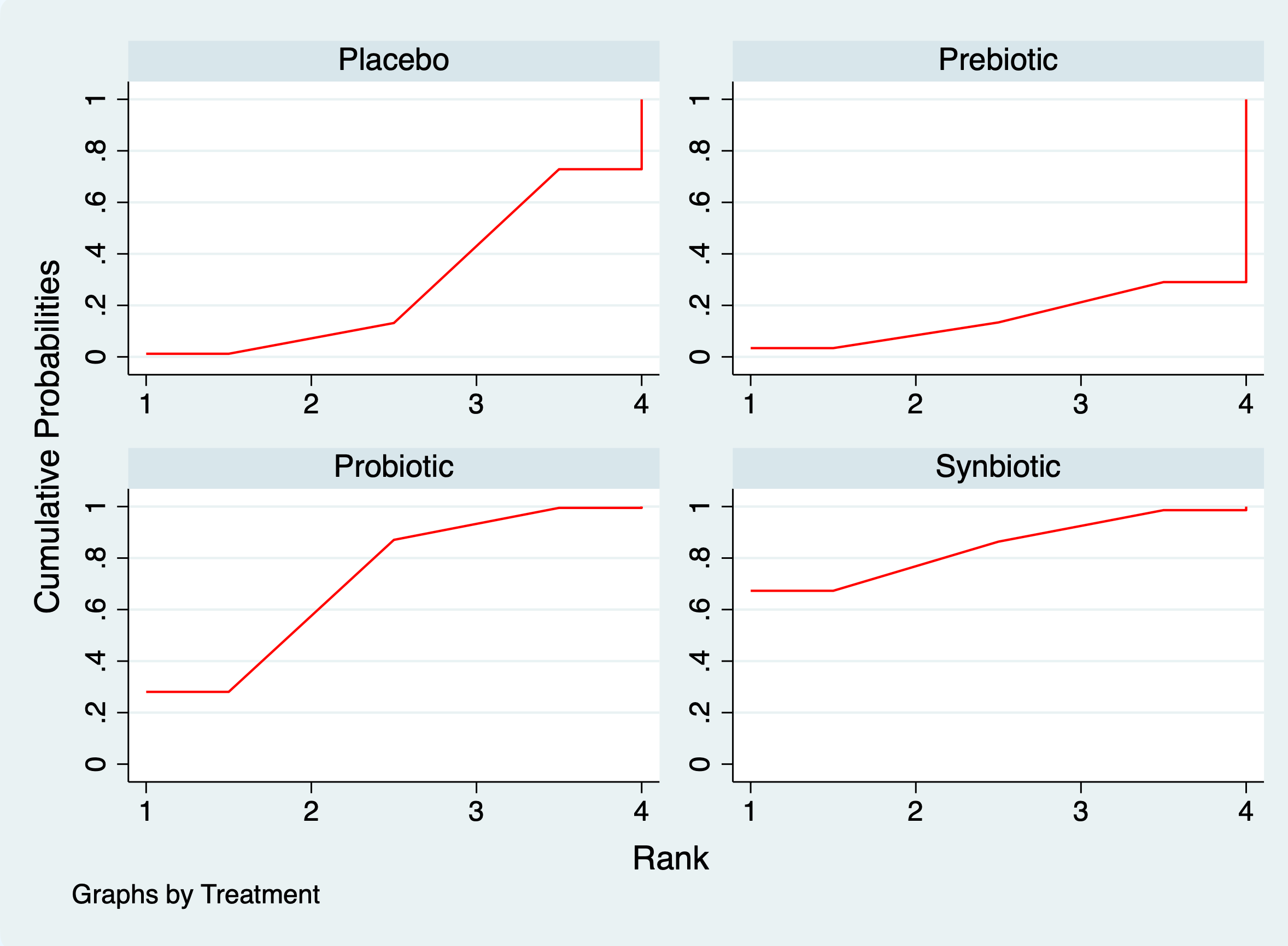


| **Treatmentt** | **SUCRA** | **PrBest** | **MeanRank** |
| --- | --- | --- | --- |
| **Placebo** | **29.1** | **1.2** | **3.1** |
| **Prebiotic** | **15.3** | **3.4** | **3.5** |
| **Probiotic** | **71.5** | **28.1** | **1.9** |
| **Synbiotic** | **84.1** | **67.3** | **1.5** |

**Figure S6.3: SUCRA and cumulative probability plots for severity of pain (primary analysis)**


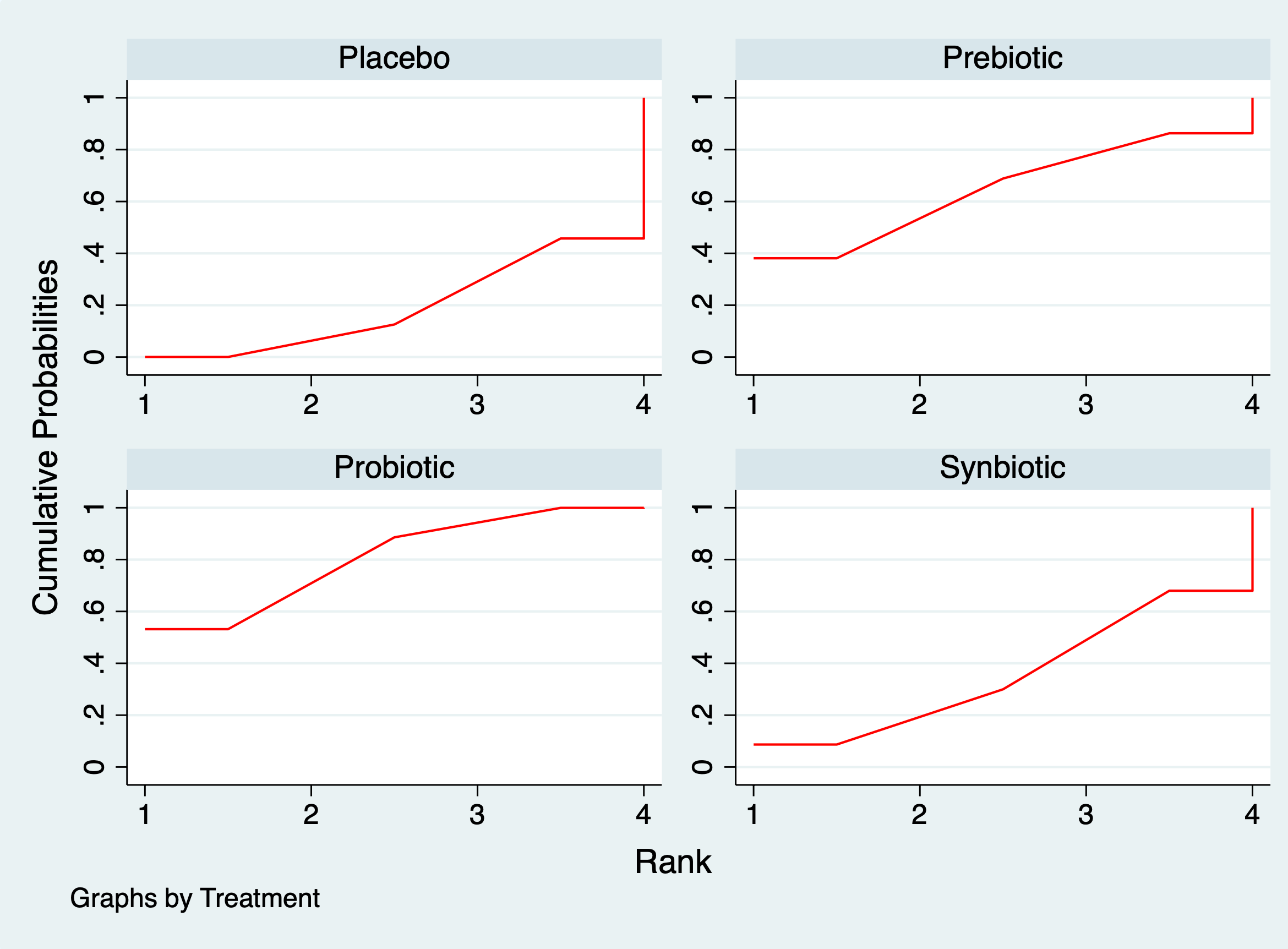


| **Treatmentt** | **SUCRA** | **PrBest** | **MeanRank** |
| --- | --- | --- | --- |
| **Placebo** | **19.5** | **0** | **3.4** |
| **Prebiotic** | **64.4** | **38.1** | **2.1** |
| **Probiotic** | **80.6** | **53.2** | **1.6** |
| **Synbiotic** | **35.5** | **8.7** | **2.9** |

**Figure S6.4: SUCRA and cumulative probability plots for frequency of pain (primary analysis)**


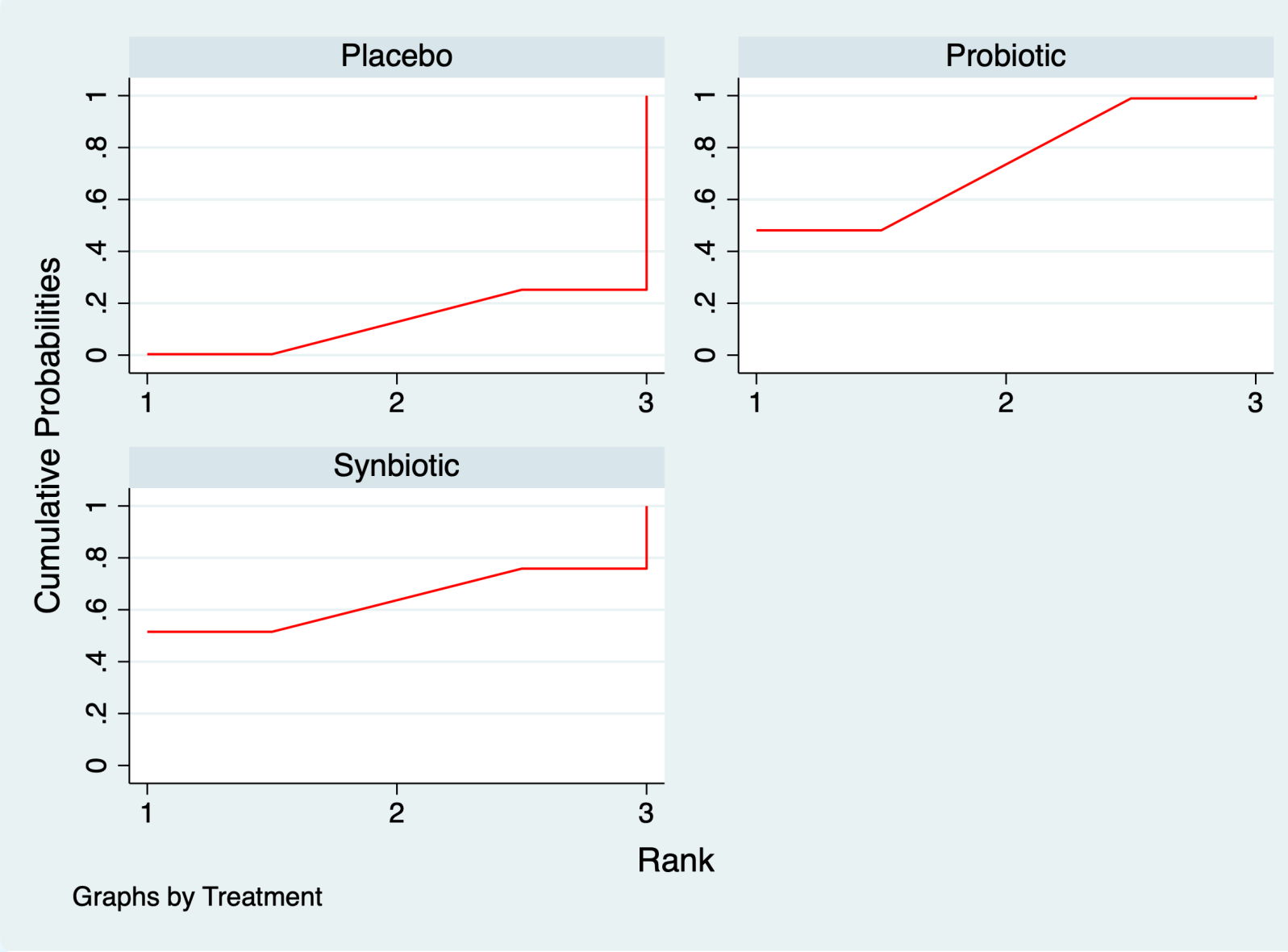


| **Treatmentt** | **SUCRA** | **PrBest** | **MeanRank** |
| --- | --- | --- | --- |
| **Placebo** | **12.8** | **0.4** | **2.7** |
| **Probiotic** | **73.5** | **48.1** | **1.5** |
| **Synbiotic** | **63.7** | **51.5** | **1.7** |

**Figure S6.5: SUCRA and cumulative probability plots for global improvement or treatment success (subgroup analysis)**


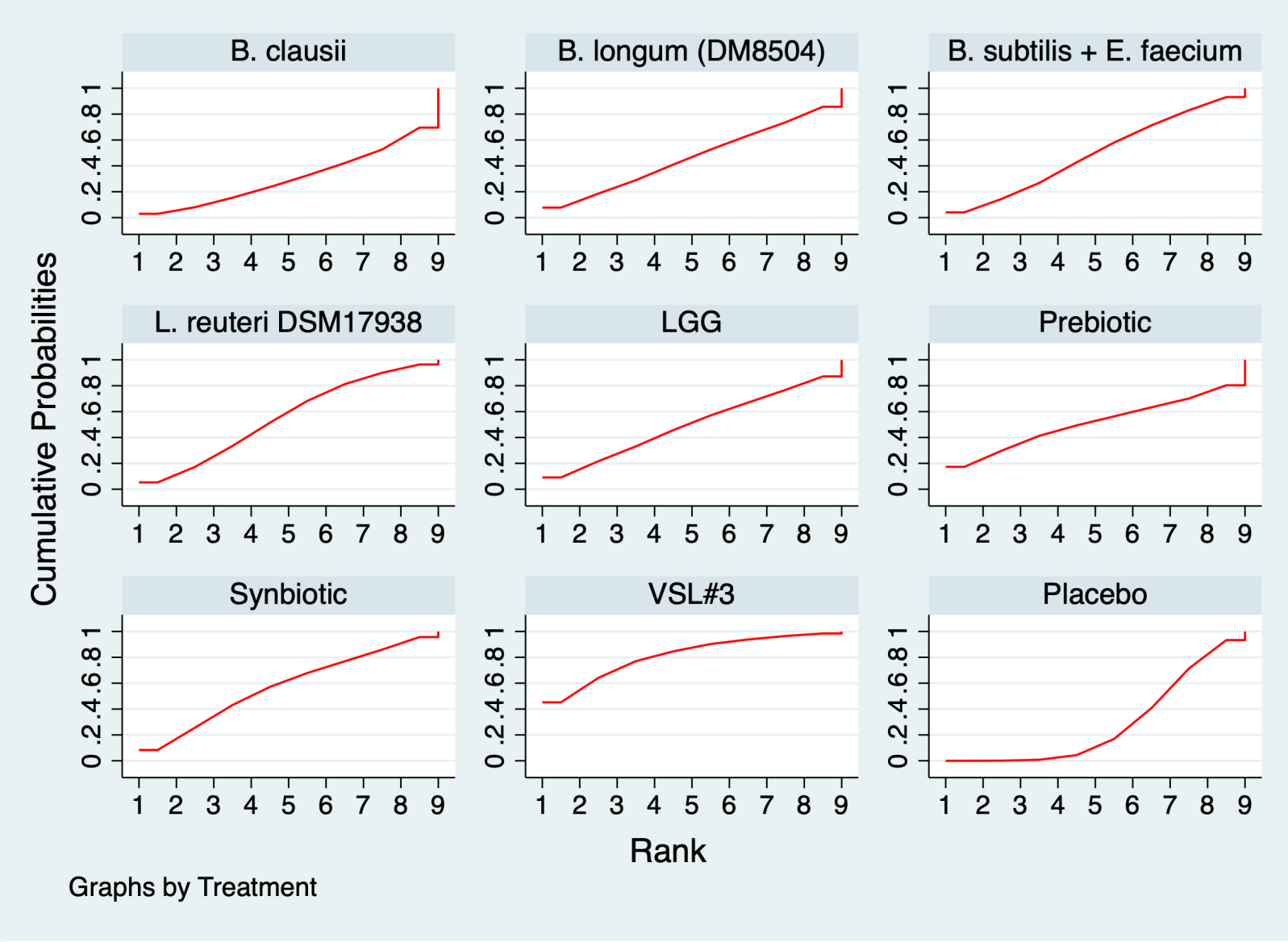


| **Treatmentt** | **SUCRA** | **PrBest** | **MeanRank** |
| --- | --- | --- | --- |
| **B. clausii** | **30.9** | **3** | **6.5** |
| **B. longum (DM8504)** | **46.5** | **7.7** | **5.3** |
| **B. subtilis + E. faecium** | **49.2** | **4.1** | **5.1** |
| **L. reuteri DSM17938** | **55.4** | **5.3** | **4.6** |
| **LGG** | **49.7** | **9.1** | **5** |
| **Prebiotic** | **51** | **17.3** | **4.9** |
| **Synbiotic** | **57.6** | **8.4** | **4.4** |
| **VSL#3** | **81.3** | **45.2** | **2.5** |
| **Placebo** | **28.5** | **0** | **6.7** |

**Figure S6.6: SUCRA and cumulative probability plots for complete resolution of pain (subgroup analysis)**


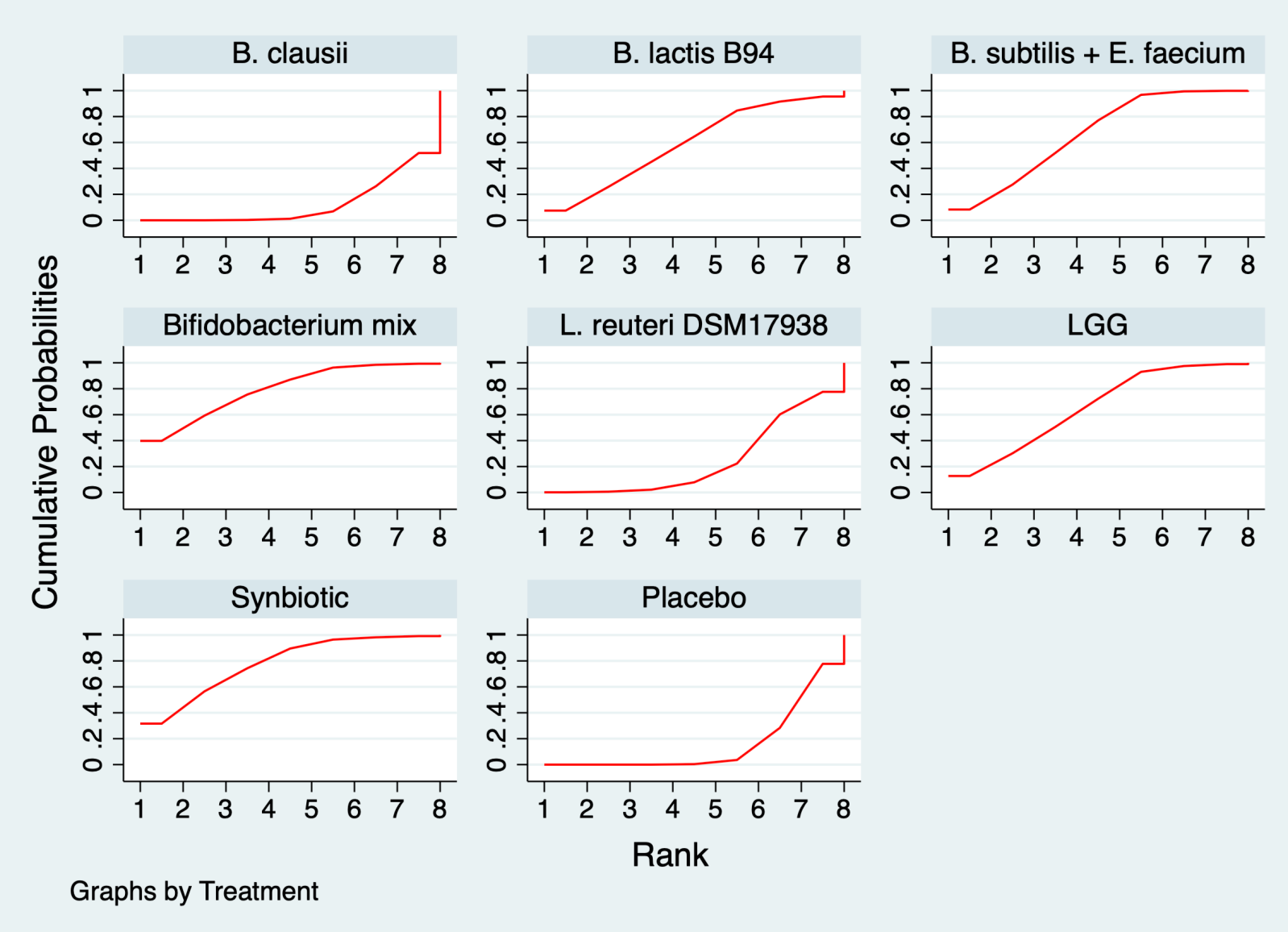


| **Treatmentt** | **SUCRA** | **PrBest** | **MeanRank** |
| --- | --- | --- | --- |
| **B. clausii** | **12.3** | **0** | **7.1** |
| **B. lactis B94** | **59.2** | **7.5** | **3.9** |
| **B. subtilis + E. faecium** | **65.9** | **8.3** | **3.4** |
| **Bifidobacterium mix** | **79.4** | **39.8** | **2.4** |
| **L. reuteri DSM17938** | **24.4** | **0.1** | **6.3** |
| **LGG** | **65.1** | **12.7** | **3.4** |
| **Synbiotic** | **78** | **31.6** | **2.5** |
| **Placebo** | **15.7** | **0** | **6.9** |

**Figure S6.7: SUCRA and cumulative probability plots for severity of pain (subgroup analysis)**


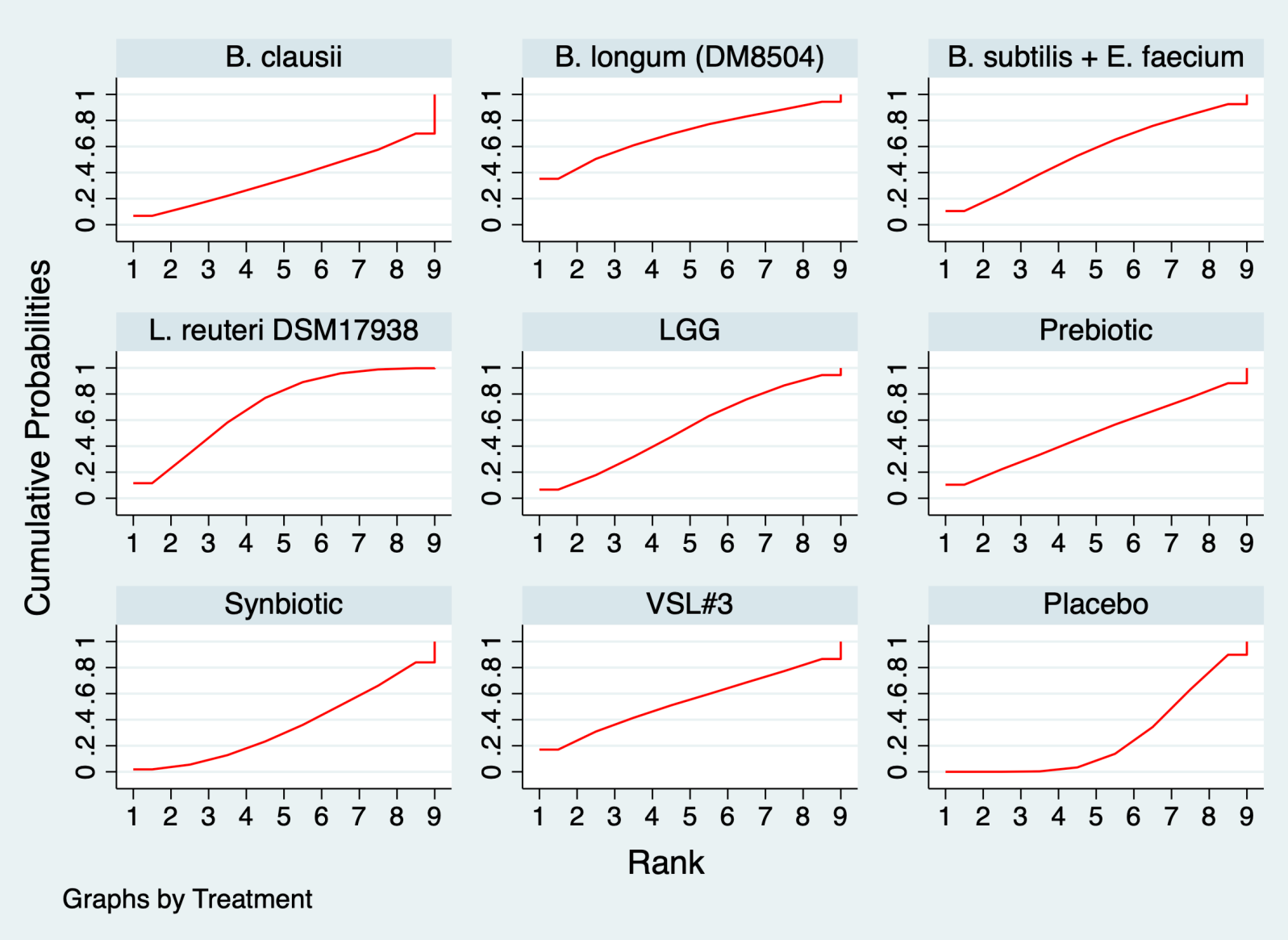


| **Treatmentt** | **SUCRA** | **PrBest** | **MeanRank** |
| --- | --- | --- | --- |
| **B. clausii** | **36.1** | **6.8** | **6.1** |
| **B. longum (DM8504)** | **69.9** | **35.2** | **3.4** |
| **B. subtilis + E. faecium** | **55.5** | **10.5** | **4.6** |
| **L. reuteri DSM17938** | **70.6** | **11.6** | **3.3** |
| **LGG** | **53** | **6.6** | **4.8** |
| **Prebiotic** | **50** | **10.4** | **5** |
| **Synbiotic** | **35.1** | **1.9** | **6.2** |
| **VSL#3** | **54.1** | **17** | **4.7** |
| **Placebo** | **25.6** | **0** | **6.9** |

**Figure S6.8: SUCRA and cumulative probability plots for frequency of pain (subgroup analysis)**


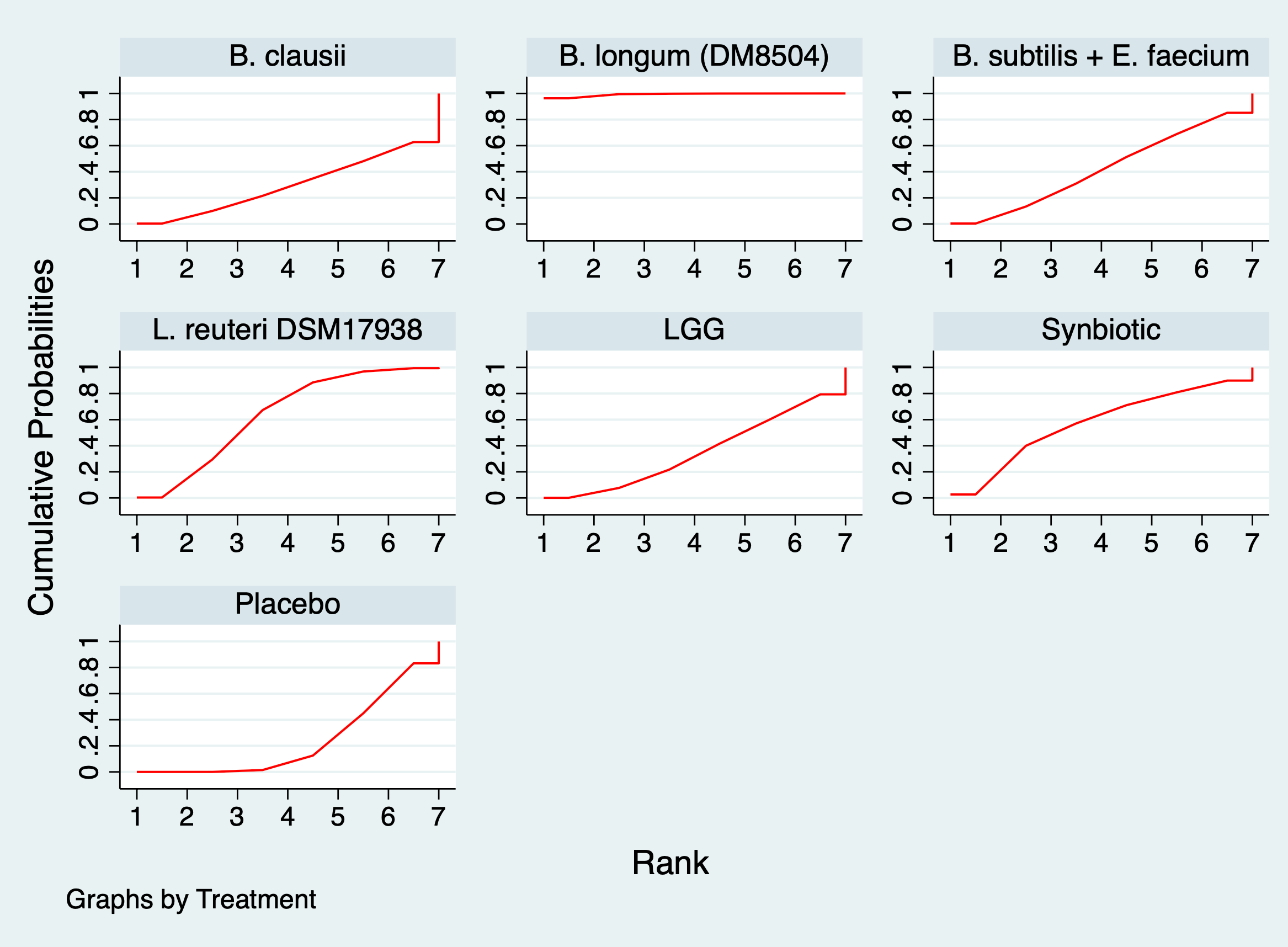


| **Treatmentt** | **SUCRA** | **PrBest** | **MeanRank** |
| --- | --- | --- | --- |
| **B. clausii** | **29.6** | **0.3** | **5.2** |
| **B. longum (DM8504)** | **99.3** | **96.3** | **1** |
| **B. subtilis + E. faecium** | **41.7** | **0.3** | **4.5** |
| **L. reuteri DSM17938** | **63.7** | **0.3** | **3.2** |
| **LGG** | **35.1** | **0.1** | **4.9** |
| **Synbiotic** | **57** | **2.7** | **3.6** |
| **Placebo** | **23.7** | **0** | **5.6** |

**Appendix 7: league table**

**Table S7.1: League table for global improvement or treatment success (primary analysis)**

| Prebiotic |  |  |  |
| --- | --- | --- | --- |
| 1.02 (0.29,3.56) | Probiotic |  |  |
| 0.91 (0.35,2.34) | 0.89 (0.39,2.03) | Synbiotic |  |
| 1.36 (0.40,4.63) | 1.33 (1.03,1.73) | 1.50 (0.69,3.27) | Placebo |

**Table S7.2: League table for complete resolution of pain (primary analysis)**

| Prebiotic |  |  |  |
| --- | --- | --- | --- |
| 0.35 (0.06,1.98) | Probiotic |  |  |
| 0.22 (0.04,1.24) | 0.64 (0.14,2.89) | Synbiotic |  |
| 0.64 (0.10,3.99) | 1.85 (1.07,3.21) | 2.89 (0.58,14.38) | Placebo |

**Table S7.3: League table for severity of pain (primary analysis)**

| Prebiotic |  |  |  |
| --- | --- | --- | --- |
| 0.15 (-1.17,1.47) | Probiotic |  |  |
| -0.40 (-1.72,0.92) | -0.55 (-1.66,0.56) | Synbiotic |  |
| -0.57 (-1.90,0.76) | -0.72 (-1.16,-0.28) | -0.17 (-1.23,0.88) | Placebo |

**Table S7.4: League table for frequency of pain (primary analysis)**

| Probiotic |  |  |
| --- | --- | --- |
| 0.05 (-3.18,3.29) | Synbiotic |  |
| -1.04 (-1.98,-0.11) | -1.10 (-4.19,2.00) | Placebo |

**Table S7.5: League table for global improvement or treatment success (subgroup analysis)**

| **B. clausii** |  |  |  |  |  |  |  |  |
| --- | --- | --- | --- | --- | --- | --- | --- | --- |
| **0.77 (0.17,3.52)** | **B. longum (DM8504)** |  |  |  |  |  |  |  |
| **0.72 (0.19,2.71)** | **0.95 (0.25,3.56)** | **B. subtilis + E. faecium** |  |  |  |  |  |  |
| **0.67 (0.19,2.39)** | **0.87 (0.24,3.14)** | **0.92 (0.33,2.58)** | **L. reuteri DSM17938** |  |  |  |  |  |
| **0.70 (0.15,3.27)** | **0.92 (0.20,4.29)** | **0.97 (0.25,3.71)** | **1.05 (0.29,3.84)** | **LGG** |  |  |  |  |
| **0.69 (0.09,5.10)** | **0.90 (0.12,6.68)** | **0.95 (0.15,6.08)** | **1.03 (0.17,6.38)** | **0.98 (0.13,7.35)** | **Prebiotic** |  |  |  |
| **0.63 (0.13,2.99)** | **0.82 (0.17,3.92)** | **0.87 (0.22,3.40)** | **0.94 (0.25,3.53)** | **0.89 (0.18,4.33)** | **0.91 (0.26,3.17)** | **Synbiotic** |  |  |
| **0.36 (0.08,1.75)** | **0.47 (0.10,2.29)** | **0.50 (0.13,1.99)** | **0.54 (0.14,2.07)** | **0.52 (0.11,2.53)** | **0.53 (0.07,4.04)** | **0.58 (0.12,2.90)** | **VSL#3** |  |
| **0.94 (0.32,2.75)** | **1.23 (0.42,3.61)** | **1.30 (0.60,2.79)** | **1.40 (0.71,2.79)** | **1.34 (0.45,4.01)** | **1.36 (0.25,7.34)** | **1.50 (0.48,4.64)** | **2.59 (0.82,8.16)** | **Placebo** |

**Table S7.6: League table for complete resolution of pain (subgroup analysis)**

| **B. clausii** |  |  |  |  |  |  |  |
| --- | --- | --- | --- | --- | --- | --- | --- |
| **0.32 (0.06,1.60)** | **B. lactis B94** |  |  |  |  |  |  |
| **0.29 (0.10,0.81)** | **0.92 (0.17,5.07)** | **B. subtilis + E. faecium** |  |  |  |  |  |
| **0.18 (0.04,0.89)** | **0.58 (0.07,4.65)** | **0.63 (0.12,3.35)** | **Bifidobacterium mix** |  |  |  |  |
| **0.78 (0.29,2.13)** | **2.46 (0.45,13.40)** | **2.69 (0.85,8.45)** | **4.27 (0.81,22.46)** | **L. reuteri DSM17938** |  |  |  |
| **0.29 (0.08,1.04)** | **0.92 (0.14,5.95)** | **1.00 (0.25,4.02)** | **1.60 (0.25,9.99)** | **0.37 (0.09,1.47)** | **LGG** |  |  |
| **0.20 (0.04,1.00)** | **0.64 (0.19,2.16)** | **0.70 (0.13,3.77)** | **1.11 (0.14,8.81)** | **0.26 (0.05,1.38)** | **0.70 (0.11,4.41)** | **Synbiotic** |  |
| **0.92 (0.50,1.69)** | **2.88 (0.65,12.87)** | **3.14 (1.37,7.18)** | **5.00 (1.17,21.46)** | **1.17 (0.53,2.59)** | **3.13 (1.03,9.56)** | **4.50 (1.03,19.60)** | **Placebo** |

**Table S7.7: League table for severity of pain (subgroup analysis)**

| **B. clausii** |  |  |  |  |  |  |  |  |
| --- | --- | --- | --- | --- | --- | --- | --- | --- |
| **1.02 (-1.83,3.87)** | **B. longum (DM8504)** |  |  |  |  |  |  |  |
| **0.53 (-1.93,2.98)** | **-0.50 (-2.98,1.99)** | **B. subtilis + E. faecium** |  |  |  |  |  |  |
| **0.83 (-1.31,2.98)** | **-0.19 (-2.37,1.99)** | **0.31 (-1.32,1.94)** | **L. reuteri DSM17938** |  |  |  |  |  |
| **0.47 (-1.92,2.85)** | **-0.55 (-2.97,1.87)** | **-0.06 (-1.99,1.88)** | **-0.36 (-1.88,1.16)** | **LGG** |  |  |  |  |
| **0.39 (-2.28,3.06)** | **-0.63 (-3.33,2.07)** | **-0.13 (-2.41,2.14)** | **-0.44 (-2.38,1.50)** | **-0.08 (-1.75,1.60)** | **Prebiotic** |  |  |  |
| **0.07 (-2.33,2.46)** | **-0.96 (-3.38,1.47)** | **-0.46 (-2.40,1.48)** | **-0.77 (-2.30,0.76)** | **-0.40 (-2.07,1.26)** | **-0.33 (-2.01,1.35)** | **Synbiotic** |  |  |
| **0.53 (-2.30,3.37)** | **-0.49 (-3.35,2.38)** | **0.01 (-2.46,2.48)** | **-0.30 (-2.46,1.86)** | **0.06 (-2.34,2.47)** | **0.14 (-2.54,2.83)** | **0.47 (-1.94,2.88)** | **VSL#3** |  |
| **-0.07 (-2.07,1.92)** | **-1.09 (-3.13,0.94)** | **-0.60 (-2.03,0.83)** | **-0.91 (-1.69,-0.13)** | **-0.54 (-1.85,0.76)** | **-0.46 (-2.24,1.31)** | **-0.14 (-1.45,1.18)** | **-0.61 (-2.62,1.41)** | **Placebo** |

**Table S7.8: League table for frequency of pain (subgroup analysis)**

| **B. clausii** |  |  |  |  |  |  |
| --- | --- | --- | --- | --- | --- | --- |
| **4.59 (1.04,8.14)** | **B. longum (DM8504)** |  |  |  |  |  |
| **0.47 (-2.53,3.47)** | **-4.12 (-7.23,-1.01)** | **B. subtilis + E. faecium** |  |  |  |  |
| **1.13 (-1.55,3.82)** | **-3.46 (-6.27,-0.65)** | **0.67 (-1.40,2.73)** | **L. reuteri DSM17938** |  |  |  |
| **0.22 (-2.78,3.21)** | **-4.37 (-7.48,-1.26)** | **-0.25 (-2.71,2.21)** | **-0.91 (-2.98,1.15)** | **LGG** |  |  |
| **1.10 (-2.40,4.59)** | **-3.49 (-7.08,0.10)** | **0.63 (-2.41,3.68)** | **-0.03 (-2.77,2.70)** | **0.88 (-2.16,3.92)** | **Synbiotic** |  |
| **-0.00 (-2.44,2.44)** | **-4.59 (-7.16,-2.01)** | **-0.47 (-2.21,1.27)** | **-1.13 (-2.25,-0.01)** | **-0.22 (-1.95,1.52)** | **-1.10 (-3.60,1.40)** | **Placebo** |

**Appendix 8: Sensitivity analyses**

| Intervention | **Global improvement or treatment success** | | | Complete resolution of pain | | | Severity of pain | | | Frequency of pain | | |
| --- | --- | --- | --- | --- | --- | --- | --- | --- | --- | --- | --- | --- |
| Primary NMA | **Original** | Excluding high-risk trials | Excluding non-Rome criteria | **Original** | Excluding high-risk trials | Excluding non-Rome criteria | **Original** | Excluding high-risk trials | Excluding non-Rome criteria | **Original** | Excluding high-risk trials | Excluding non-Rome criteria |
| Prebiotic | 1.36 (0.40,4.63) | 1.34 (0.39, 4.57) | 1.34 (0.39, 4.57) | 0.64 (0.10,3.99) | 0.63 (0.09, 3.93) | 0.63 (0.09, 3.93) | -0.57 (-1.90,0.76) | -0.56 (-1.88, 0.77) | -0.56 (-1.88, 0.77) | NA | NA | NA |
| Probiotic | 1.33 (1.03,1.73) | 1.31 (1.02, 1.70) | 1.31 (1.02, 1.70) | 1.85 (1.07,3.21) | 1.83 (1.06, 3.17) | 1.83 (1.06, 3.17) | -0.72 (-1.16,-0.28) | -0.70 (-1.14, -0.27) | -0.70 (-1.14, -0.27) | -1.04 (-1.98,-0.11) | -1.04 (-1.98, -0.11) | -1.04 (-1.98, -0.11) |
| Synbiotic | 1.50 (0.69,3.27) | 1.48 (0.68, 3.22) | 1.48 (0.68, 3.22) | 2.89 (0.58,14.38) | 2.83 (0.57, 14.21) | 2.83 (0.57, 14.21) | -0.17 (-1.23,0.88) | -0.16 (-1.22, 0.87) | -0.16 (-1.22, 0.87) | -1.10 (-4.19,2.00) | -1.10 (-4.19, 2.00) | -1.10 (-4.19, 2.00) |
| Subgroup NMA | **Original** | Excluding high-risk trials | Excluding non-Rome criteria | **Original** | Excluding high-risk trials | Excluding non-Rome criteria | **Original** | Excluding high-risk trials | Excluding non-Rome criteria | **Original** | Excluding high-risk trials | Excluding non-Rome criteria |
| B. clausii | 0.94 (0.32,2.75) | 0.93 (0.31, 2.78) | 0.93 (0.31, 2.78) | 0.92 (0.50,1.69) | 0.90 (0.48, 1.71) | 0.90 (0.48, 1.71) | -0.07 (-2.07,1.92) | -0.06 (-2.05, 1.94) | -0.06 (-2.05, 1.94) | -0.00 (-2.44,2.44) | -0.02 (-2.43, 2.40) | -0.02 (-2.43, 2.40) |
| B. longum (DM8504) | 1.23 (0.42,3.61) | NA | NA | NA | NA | NA | -1.09 (-3.13,0.94) | NA | NA | -4.59 (-7.16,-2.01) | NA | NA |
| B. lactis B94 | NA | NA | NA | 2.88 (0.65,12.87) | 2.85 (0.63, 12.80) | 2.85 (0.63, 12.80) | NA | NA | NA | NA | NA | NA |
| B. subtilis + E. faecium | 1.30 (0.60,2.79) | NA | NA | 3.14 (1.37,7.18) | NA | NA | -0.60 (-2.03,0.83) | NA | NA | -0.47 (-2.21,1.27) | NA | NA |
| Bifidobacterium mix | NA | NA | NA | 5.00 (1.17,21.46) | 4.97 (1.15, 21.30) | 4.97 (1.15, 21.30) | NA | NA | NA | NA | NA | NA |
| L. reuteri DSM17938 | 1.40 (0.71,2.79) | 1.38 (0.69, 2.76) | 1.38 (0.69, 2.76) | 1.17 (0.53,2.59) | 1.15 (0.51, 2.55) | 1.15 (0.51, 2.55) | -0.91 (-1.69,-0.13) | -0.88 (-1.65, -0.11) | -0.88 (-1.65, -0.11) | -1.13 (-2.25,-0.01) | -1.09 (-2.22, -0.03) | -1.09 (-2.22, -0.03) |
| LGG | 1.34 (0.45,4.01) | 1.32 (0.43, 3.97) | 1.32 (0.43, 3.97) | 3.13 (1.03,9.56) | 3.11 (1.01, 9.52) | 3.11 (1.01, 9.52) | -0.54 (-1.85,0.76) | -0.53 (-1.83, 0.77) | -0.53 (-1.83, 0.77) | -0.22 (-1.95,1.52) | -0.20 (-1.92, 1.49) | -0.20 (-1.92, 1.49) |
| Prebiotic | 1.36 (0.25,7.34) | 1.34 (0.24, 7.22) | 1.34 (0.24, 7.22) | NA | NA | NA | -0.46 (-2.24,1.31) | -0.45 (-2.23, 1.30) | -0.45 (-2.23, 1.30) | NA | NA | NA |
| Synbiotic | 1.50 (0.48,4.64) | 1.48 (0.47, 4.59) | 1.48 (0.47, 4.59) | 4.50 (1.03,19.60) | 4.45 (1.01, 19.41) | 4.45 (1.01, 19.41) | -0.14 (-1.45,1.18) | -0.12 (-1.43, 1.16) | -0.12 (-1.43, 1.16) | -1.10 (-3.60,1.40) | -1.08 (-3.58, 1.36) | -1.08 (-3.58, 1.36) |
| VSL#3 | 2.59 (0.82,8.16) | 2.55 (0.81, 8.04) | 2.55 (0.81, 8.04) | NA | NA | NA | -0.61 (-2.62,1.41) | -0.60 (-2.60, 1.39) | -0.60 (-2.60, 1.39) | NA | NA | NA |

**Appendix 9: The meta-regression of the factors that may lead to differences to the main outcome indicators**

| **Factors** | **Global improvement or treatment success** | | | **Complete resolution of pain** | | | **Severity of pain** | | | **Frequency of pain** | | |
| --- | --- | --- | --- | --- | --- | --- | --- | --- | --- | --- | --- | --- |
|  | Coefficient | 95% CI | P value | Coefficient | 95% CI | P value | Coefficient | 95% CI | P value | Coefficient | 95% CI | P value |
| Dosage | 0.04 | -0.05, 0.12 | 0.41 | 0.03 | -0.08, 0.14 | 0.55 | -0.06 | -0.21, 0.09 | 0.43 | -0.05 | -0.22, 0.12 | 0.49 |
| Duration | 0.02 | -0.07, 0.10 | 0.62 | 0.05 | -0.09, 0.18 | 0.48 | -0.03 | -0.16, 0.10 | 0.63 | -0.07 | -0.20, 0.06 | 0.28 |
| Single vs. multi | 0.06 | -0.11, 0.23 | 0.49 | 0.09 | -0.15, 0.32 | 0.46 | -0.04 | -0.21, 0.14 | 0.67 | -0.02 | -0.19, 0.15 | 0.81 |
| Country | -0.03 | -0.18, 0.12 | 0.69 | 0.07 | -0.10, 0.24 | 0.42 | 0.05 | -0.09, 0.19 | 0.52 | -0.01 | -0.17, 0.15 | 0.91 |
| Age | 0.02 | -0.10, 0.13 | 0.73 | 0.04 | -0.08, 0.16 | 0.51 | -0.06 | -0.19, 0.08 | 0.38 | -0.03 | -0.18, 0.12 | 0.67 |
